# A new quinoline-based chemical probe inhibits the autophagy-related cysteine protease ATG4B

**DOI:** 10.1038/s41598-018-29900-x

**Published:** 2018-08-03

**Authors:** D. Bosc, L. Vezenkov, S. Bortnik, J. An, J. Xu, C. Choutka, A. M. Hannigan, S. Kovacic, S. Loo, P. G. K. Clark, G. Chen, R. N. Guay-Ross, K. Yang, W. H. Dragowska, F. Zhang, N. E. Go, A. Leung, N. S. Honson, T. A. Pfeifer, M. Gleave, M. Bally, S. J. Jones, S. M. Gorski, R. N. Young

**Affiliations:** 1 Department of Chemistry, Simon Fraser University, Burnaby, BC V5A 1S6 Canada; 2 Canada’s Michael Smith Genome Sciences Centre, BC Cancer Agency, Vancouver, BC V5Z 4E6 Canada; 3 Interdisciplinary Oncology Program, University of British Columbia, Vancouver, Canada; 4 Department of Molecular Biology and Biochemistry, Simon Fraser University, Burnaby, BC V5A 1S6 Canada; 5 Experimental Therapeutics, BC Cancer Agency, Vancouver, BC V5Z 4E6 Canada; 6 Department of Urologic Sciences and Vancouver Prostate Centre, University of British Columbia, Vancouver, BC V6H 3Z6 Canada; 7 Centre for Drug Research and Development, 2405 Wesbrook Mall – 4th Floor, Vancouver, BC V6T 1Z3 Canada; 8 Present Address: Inserm, Institut Pasteur de Lille, U1177 Drugs & Molecules for Living Systems, Université de Lille, F-59000 Lille, France; 9 Present Address: Institut des Biomolécules Max Mousseron (IBMM), UMR 5247 CNRS, Université de Montpellier, ENSCM, Faculté de Pharmacie, 15 avenue Charles Flahault, 34093 Montpellier, France

## Abstract

The cysteine protease ATG4B is a key component of the autophagy machinery, acting to proteolytically prime and recycle its substrate MAP1LC3B. The roles of ATG4B in cancer and other diseases appear to be context dependent but are still not well understood. To help further explore ATG4B functions and potential therapeutic applications, we employed a chemical biology approach to identify ATG4B inhibitors. Here, we describe the discovery of **4–28**, a styrylquinoline identified by a combined computational modeling, *in silico* screening, high content cell-based screening and biochemical assay approach. A structure-activity relationship study led to the development of a more stable and potent compound **LV-320**. We demonstrated that **LV-320** inhibits ATG4B enzymatic activity, blocks autophagic flux in cells, and is stable, non-toxic and active *in vivo*. These findings suggest that **LV-320** will serve as a relevant chemical tool to study the various roles of ATG4B in cancer and other contexts.

## Introduction

Autophagy has been associated with numerous disorders including neurodegenerative diseases, metabolic diseases, cardiovascular diseases, infectious diseases and cancer^1–3^, resulting in subsequent efforts to discover and develop target-selective and potent modulators of the autophagy process^3–9^. Although several autophagy modulators, both activators and inhibitors, have been described in the literature, few of them possess good pharmacokinetic properties, specificity and potency (reviewed in Triola and Vakifahmetoglu-Norberg)^10,11^. Chloroquine and other lysosomal inhibitors^12^ have shown utility in many disease models and spurred the initiation of numerous cancer clinical trials^13^. While lysosomal inhibitors may prove useful in some contexts, their effects are at least partially autophagy-independent^14^, leaving autophagy-related (ATG) protein inhibitors still largely unexplored.

ATG4B is an autophagy protein of interest as a potential therapeutic target due to its roles in autophagosome formation and maturation^15–17^. Proteolytic cleavage by ATG4B of the cytoplasmic pro-microtubule-associated-protein 1 light chain 3B (pro-MAP1LC3B or pro-LC3B) generates LC3B-I that displays a C-terminal glycine. With mediation by ATG7, ATG3 and the ATG5-ATG12 complex, this glycine is then lipidated with phosphatidylethanolamine generating LC3B-II. The conjugation enables the lipidated form to anchor to the isolation membrane during the formation of the autophagosome^18^. After the elongation of the membrane, a subsequent deconjugation of LC3B-II by ATG4B on the cytosolic face of the autophagosome releases LC3B-I^19^, which was shown to implement the fusion of the autophagosome with lysosomes and promote autophagic flux in the analogous yeast system^20^. Thus, ATG4B is important for autophagosome biogenesis by processing and recycling LC3 forms^21,22^ and a reduction in ATG4B protein and/or activity was shown to lead to an accumulation of LC3B-II due to blocked recycling^22–24^.

In humans, there are four ATG4 family members (ATG4A, ATG4B, ATG4C and ATG4D) but their individual functions are not yet well characterized. ATG4B was shown to have the highest affinity and broadest spectrum of cleavage activity against autophagy-related substrates, LC3B, ATG8L, GATE16 and GABARAP^25^. ATG4B knockout mice show a reduction in basal and starvation-induced autophagy, but are viable and fertile^26,27^. Several studies have shown that dysregulation of ATG4B is implicated in cancer^24^, inflammatory bowel diseases^28^, lung fibrosis^29^ or hepatitis C virus infection^30^, but the role of ATG4B in these and other diseases is still unclear. For example, Liu *et al*.^31^ reported that ATG4B promotes proliferation in colorectal cancer cells via an autophagy-independent pathway. In prostate cancer cells, inhibition of ATG4B with a dominant negative form of the protease resulted in a cell line-specific sensitivity to chemotherapy and modulation of autophagy^32^. Similarly, in cervical cancer, decreased levels of ATG4B enhanced cell sensitivity to the anticancer drug pirarubicin^33^. In breast cancer, one study reported that ATG4B inhibited growth of triple-negative breast cancer cell lines and a candidate agonist of ATG4B, Flubendazole, displayed anti-proliferative effects^34^. Another study identified an association between HER2 and ATG4B and showed that HER2 positive breast cancer cells are sensitive to ATG4B inhibition under stress conditions^35^. Collectively, these studies indicate context- and tissue-dependent roles of ATG4B.

Some active site directed irreversible inhibitors of ATG4B are known^36–39^ but such inhibitors generally have issues of selectivity and metabolic stability that limit their use, especially *in vivo*. Recently, a small molecule, NCS185058, was reported to be an inhibitor of ATG4B and to inhibit LC3 lipidation and autophagy. This inhibitor was reported to suppress autophagy and curb the *in vivo* growth of osteosarcoma tumors^40^ and glioblastoma tumors^41^. While NCS185058 may have potential in therapies for bone cancer and other tumor types, the pharmacokinetic properties, selectivity, and *in vivo* enzyme inhibitory potency of this compound have not yet been reported.

Identification of further molecular probes with improved potency, cell-permeability, pharmacokinetic properties and selectivity will be beneficial to explore in depth the pathological roles of ATG4B and its potential as a drug target. Moreover, it is important to have several structurally unrelated molecular probes available to reliably define the role of intervention with a macromolecular target in biology^42^.

In this context, we set out to develop new small molecule inhibitors of ATG4B. Herein is described a compound, **4–28**, discovered from an *in silico* and function-based screening effort. Its structure-based optimization led to **LV-320**, a more potent inhibitor of ATG4B, with an excellent pharmacokinetic profile that we report here along with its initial characterization *in vitro* and *in vivo*.

## Results

### Computational Modeling Predicts 4 Druggable Binding Pockets in ATG4B

**Figure 1:**
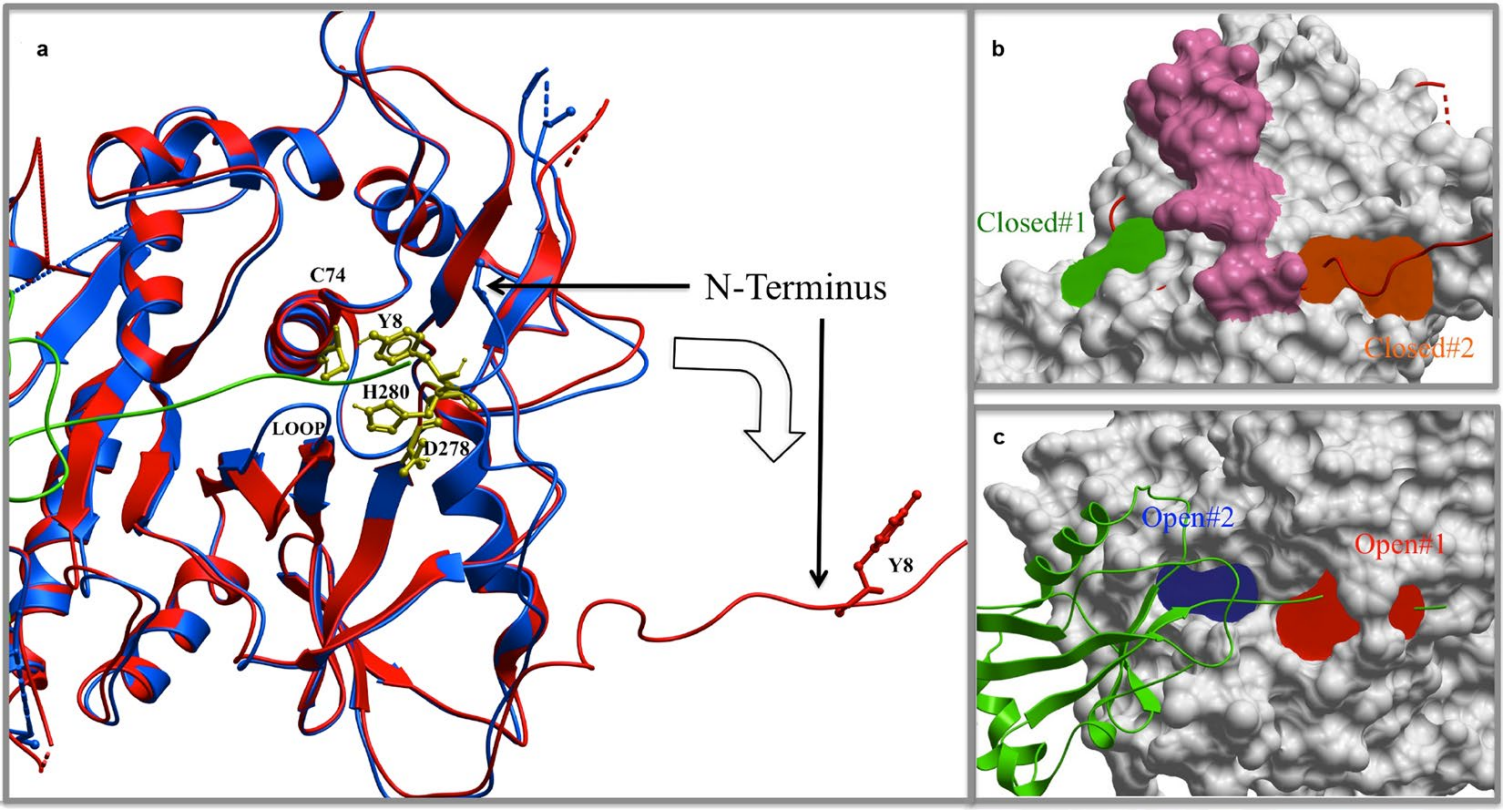
Binding pocket prediction in ATG4B. (**a**) Ribbon model to show the conformational changes from a free, inactive form (blue) to an active, substrate-binding form (red) of ATG4B. Key catalytic residues and the N-terminal Tyr8 are displayed and labelled. LC3B is in the green ribbon model. Two significant conformational changes occurred at the regulatory loop and the N-terminus. (**b**) Two pockets (green and orange) identified on the inactive conformation (grey skin model). The active conformation is displayed in red ribbon. The skin formed by the N-terminal of the inactive conformation is colored pink. (**c**) Two pockets (red and blue) identified on the surface of the active conformation (grey skin model). The LC3 is shown in green ribbon.

Several crystal structures of ATG4B are publicly available, including crystal structures of the free, closed, inactive conformation^43,44^ and LC3B complexes with catalytically inert ATG4B co-crystallized as open, “active” conformations^19,43^. To bind the LC3B substrate, both the N-terminus and the regulatory loop (amino acids 259:262) of the free form of ATG4B have to undergo conformational changes^19^ (Fig. 1a). To identify candidate inhibitors of ATG4B, we used these structural data and the PocketFinder^45^ program. We identified two pockets at the surface of the inactive structure that could be used for *in silico* screening of candidate ATG4B inhibitors. One pocket is located at the back of the regulatory loop and another at the hinge of the N-terminus (Fig. 1b). Our hypothesis was that small molecules bound to those sites could obstruct the conformational changes necessary for inactive ATG4B to become active. Using the PocketFinder program we also identified two pockets for the active conformation (Fig. 1c). One is located at the catalytic center and another at the substrate-binding interface close to the center. Any compound bound to those pockets would directly interfere with the LC3B-ATG4B interaction.

### Large-scale *In Silico* Screening and High Content Screening Identify Candidate Small Molecule Inhibitors of ATG4B

To identify candidate small molecule inhibitors of ATG4B, a computational screen was carried out using ICM^46^. Small molecule databases of National Cancer Institute (NCI, 230,000 compounds) and Chembridge (500,000 compounds) were screened. Each compound from the databases was docked to the four pockets with the “flexible ligand – rigid receptor” protocol^47^. Following database screening, the best scoring compounds were inspected visually and evaluated according to their chemical and drug-like properties, as well as three-dimensional conformations of the docked ligand-receptor complex. To help identify ATG4B-specific compounds for biological validation, all selected candidates were docked to a pocket database of all human protease and ubiquitin-like proteins for which crystal structures are available. Compounds that docked to those proteins better than to ATG4B were removed (for detailed description of the methodology see Supplemental Information).

**Figure 2:**
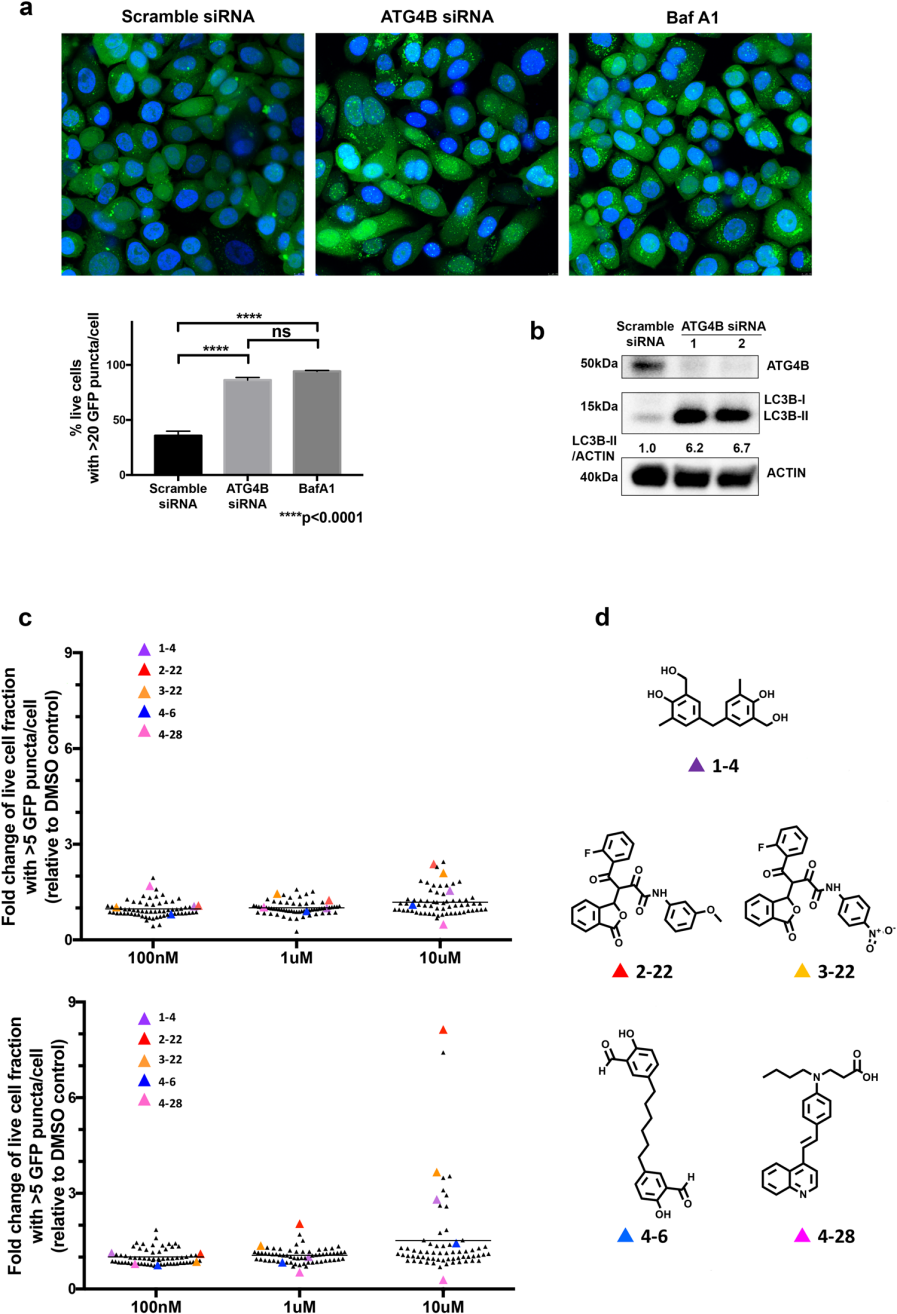
Screening of candidate ATG4B inhibitors. (**a**) Representative images of SKBR3-hrGFP-LC3B cells treated with control scramble-siRNA, ATG4B-siRNA, or bafilomycin A1 with quantitation of GFP-LC3B puncta shown in the bar graph below. Five randomly selected fields for each condition were analyzed in 2 independent experiments; error bars, SEM. (**b**) Representative western blot (n = 3) shows elevated LC3B-II levels in SKBR3 cells treated with ATG4B-siRNA compared to the control scramble-siRNA. Full-length blots are presented in Fig. S1. (**c**) Effect of small molecule compounds on the fraction of live cells with greater than five GFP-LC3B puncta in SKBR3 hrGFP-LC3 cells. Compounds were tested at three concentrations (100 nM, 1 μM and 10 μM) and two time-points, 6 h (top graph) and 24 h (bottom graph). The average values (normalized to DMSO vehicle control) from 3 independent experiments are shown. Colored triangles indicate 5 compounds that showed a statistically significant difference (p < 0.05) compared to vehicle control at one or more concentrations and timepoints and also inhibited ATG4B enzyme activity in a dose dependent manner (Table S1). Of these 5 compounds, only **2–22** and **3–22** showed a statistically significant difference at the 6 h time-point, while all 5 compounds showed a significant difference at one or more concentrations at the 24 h time-point. (**d**) Structure of 5 inhibitors of ATG4B. Compounds are named by screening codes and relate to NCI codes as depicted in Table S1. Titration curves are presented in Table S1.

One hundred of the predicted best binding ATG4B inhibitors were obtained to test for effects on GFP-LC3B puncta levels in SKBR3-hrGFP-LC3B breast cancer cells cultured in standard fed conditions. Based on our observations using ATG4B-siRNAs (Figs 2a,b and S1) and several reports in the literature^22–24^, we expected that reduced ATG4B function would primarily affect LC3B-II recycling and lead to an increase in GFP-LC3B puncta, whereas complete loss of ATG4B would also affect pro-LC3B processing and lead to a decrease in GFP-LC3B puncta formation^27^. Compounds were initially tested at three concentrations (100 nM, 1 µM and 10 µM) and two treatment periods (6 hour and 24 hour). In parallel, all compounds were tested at a single concentration (8 µM) in a FRET-LC3 ATG4B enzymatic assay developed in our group^36^. From this set of compounds, five were found to both significantly affect LC3B puncta levels at one or more concentrations and timepoints (Fig. 2c,d; compounds are depicted with their screening codes and relate to NCI codes shown in Table S1) *and* to inhibit ATG4B enzyme activity in a dose dependent manner when subsequently titrated (Table S1). Calculations predicted that compound **1–4** bound to closed binding site#1 (Fig. S2) while **2–22** and **3–22** bound to open site#2 (Fig. S3) and **4**–**6** and **4**–**28** bound to closed site#2 (Figs S4 and S5). Of these, compounds **1–4** and **4–6** are biphenols which are potentially metabolically unstable by oxidation, sulfation and glucuronidation pathways^48^. Compound **2–22** and **3–22** have electrophilic sites, which are drawbacks for drug development as they are prone to metabolic transformation and could lead to covalent bond formation with biological nucleophiles^49^. Compound **4–28** initially gave 43% inhibition at 8 µM in the screening FRET assay but was inconsistent in subsequent titrations. It was nonetheless selected for further follow-up based on its favorable structural characteristics and resemblance to the known drugs and lysosomal-related autophagy inhibitors, chloroquine and hydroxychloroquine, and to the styrylquinoline, MK-571, a selective antagonist of leukotriene D4 known to have an excellent pharmacokinetic profile in terms of oral bioavailability and long duration of action^50^. Notably however, chloroquine and hydroxychloroquine did not show significant inhibition of activity of purified ATG4B up to 175 and 200 µM respectively in our fluorescent peptide assay for ATG4B activity.

### Small Molecule 4–28 Inhibits Cleavage of LC3B in Cell-free Assays and reduces autophagy in cultured cells

**Figure 3:**
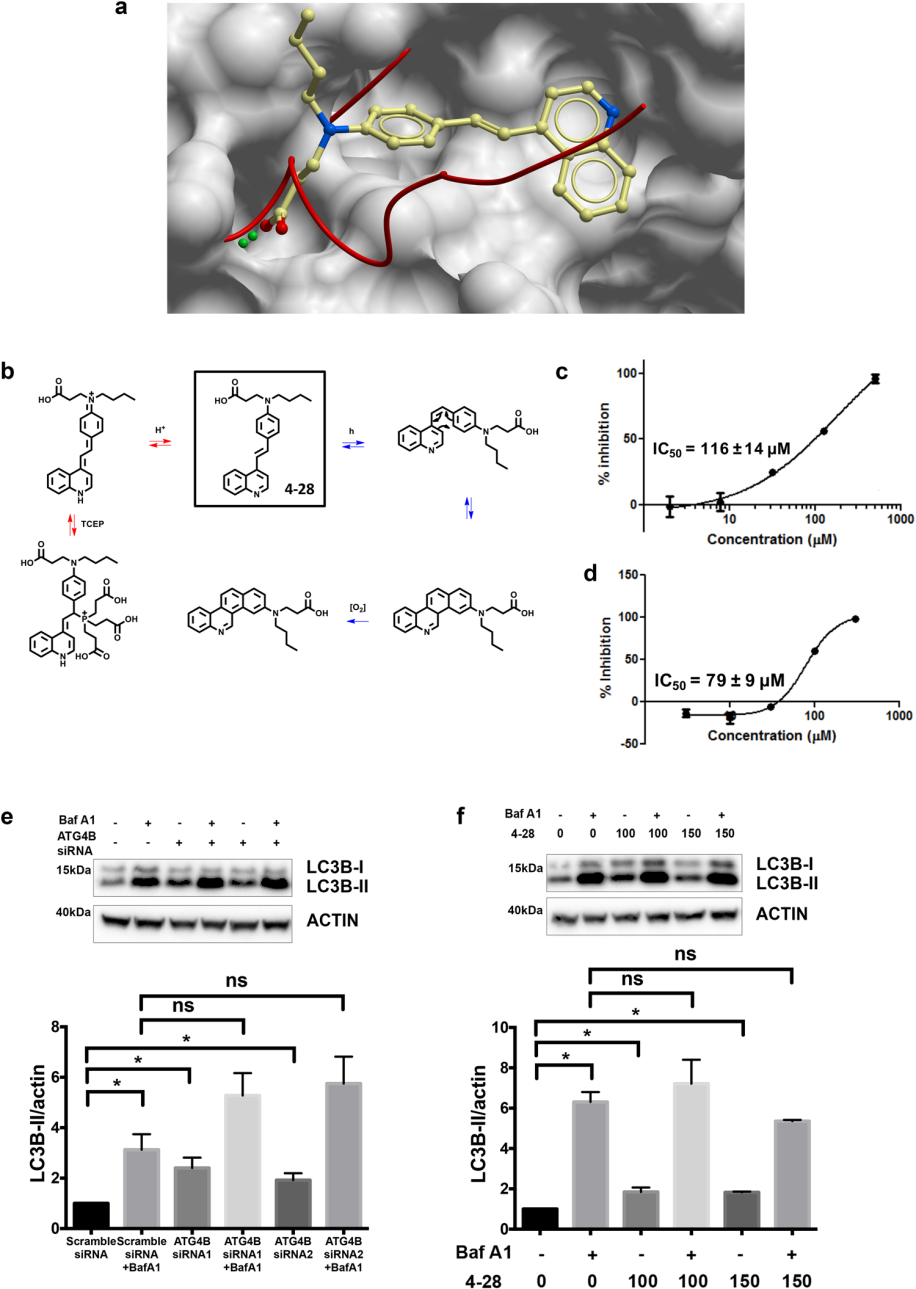
**4**–**28** inhibits ATG4B and blocks autophagic flux in cells. (**a**) Predicted binding model of compound **4–28** bound to ATG4B pocket closed#2. (**b**) Reactivity of **4–28** under UV irradiation affording the *cis*-isomer and hypothetically the cyclized form (blue pathway) and under TCEP treatment (red pathway). (**c**) Dose response curve of **4–28** obtained by mass spectrometry assay using GFP-LC3-YFP assay. n = 3; error bars, SEM (**d**) Dose response curve of **4–28** with fluorimetric assay. n = 3; error bars represent SEM (**e**) LC3B-based autophagy flux assay for ATG4B-siRNA. MCF7 cells treated with control scramble-siRNA, ATG4B-siRNA1, or ATG4B-siRNA2, in the absence or presence of bafilomycin A1 (Baf A1). n = 3; error bars, SEM; ^*^p < 0.05. Full-length blots are presented in Fig. S1. (**f**) LC3B-based autophagy flux assay for **4–28**. MCF7 cells were treated with vehicle control or the indicated amounts of **4–28**, in the absence or presence of bafilomycin A1 (Baf A1). n = 3; error bars, SEM; *p < 0.05. Full-length blots are presented in Fig. S1.

The initial inconsistent results obtained with **4–28** in our screening enzyme assay suggested that it may not be stable under the assay conditions. **4–28** is a 4-aminostyrylquinoline, a structural class known to be photo-reactive^51^. In preliminary assays under standard conditions with the reducing agent TCEP present, we noted the formation of isomers and adducts between **4–28** and TCEP (Fig. 3b). Thus, to confirm the ability of **4–28** to inhibit ATG4B activity, we used a mass spectrometry assay using a GFP-LC3-YFP substrate^36^ and by using activation of the enzyme with TCEP on solid support, which can be easily removed after the activation and prior to incubation with the inhibitor. Using this approach, we obtained a dose-response curve where **4–28** displayed an IC_50_ of 116 ± 14 µM (Fig. 3c). A similar IC_50_ (79 ± 9 µM) was obtained using a fluorometric assay optimized with minimized irradiations and with TCEP on solid support, using a small fluorogenic peptide substrate developed in our laboratory^52^ (Fig. 3d). HPLC analysis (Figs S6 and S7) showed that only 4% of the *cis*-isomer of **4–28** was formed under the assay conditions (1 irradiation at 350 nm each 15 min for 2 h) indicating that the integrity of **4–28** was preserved during the assay. By these two assays, we confirmed the ability of **4–28** to inhibit ATG4B catalytic activity.

To confirm the effects of **4–28** on autophagy, we assayed endogenous LC3B in MCF7 cells, in the presence and absence of bafilomycin A1. Similar to the effects observed with ATG4B-siRNAs (Figs 2a,b and 3e), **4**–**28** resulted in an accumulation of LC3B-II that was not additive to bafilomycin A1 (Fig. 3f). While the effects observed were modest, this observation is consistent with reduced LC3B-II recycling and reduced autophagic flux.

Despite its promising activity, **4–28** showed instability under UV irradiation as well as a propensity for addition of nucleophiles (TCEP) to the styryl double bond (as noted above). In our case, with incubation of **4–28** for 1 h under UV-irradiation, the formation of the *cis* compound was observed by HPLC and ^1^H NMR along with other compounds consistent with further electrocyclization (Figs 3b and S6 and S7). Given these observed instabilities and reactivity, a structure-activity relationship study was initiated with an aim to identify an analog with improved stability and enhanced potency against ATG4B and with a pharmacokinetic profile suitable to support *in vivo* testing.

### A Structure-Activity Relationship (SAR) Study identified Compound LV-320 with Improved Stability and ATG4B Potency

**Figure 4:**
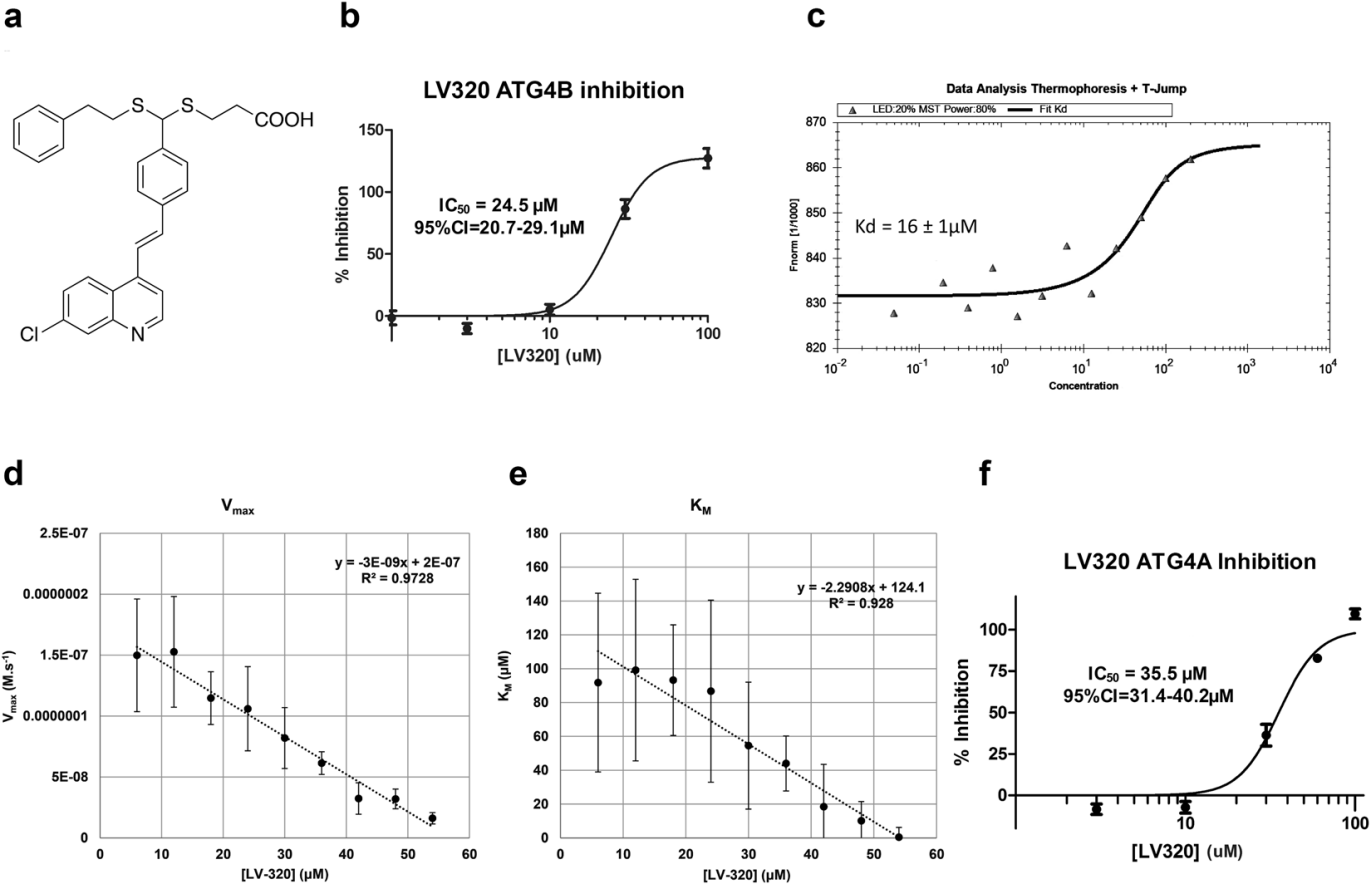
**LV-320** arose from the SAR study and binds ATG4B. (**a**) Structure of **LV-320**. (**b**) IC_50_ of LV-320 in ATG4B cleavage assay using the fluorescent peptide pim-FG-PABA-AMC as substrate; n = 3; error bars, SEM (**c**) Binding of **LV-320** with ATG4B determined by MST. (n = 3) (**d**) and (**e**). **LV-320** depresses both the V_max_ (**d**) and K_M_ (**e**) in an ATG4B enzymatic assay with a fluorogenic peptide substrate, concordant with an uncompetitive mode of inhibition. Error bars, SEM. Experiments were performed with technical quadruplicates and representative biological duplication. (**f**) IC_50_ of **LV-320** in ATG4A cleavage assay using pim-FG-PABA-AMC as substrate. n = 4; error bars, SEM.

In our binding models compound **4–28** was predicted to bind to pocket closed#2 (Figs 1b, 3a, and S5). The volume of **4–28** is 390.7 Å^3^ and its binding pocket is 486.8 Å^3^. There are two crystal structures of ATG4B with closed conformation in PDB from two independent labs (PDB entries 2CY7 and 2D1I). This pocket is conserved in both closed conformational structures, but not in any of the three available active conformation structures (2ZZP, 2Z0D and 2Z0E) due to the large conformational changes of the N-terminal tails and a segment of another loop (Cys301:Pro305) which formed part of the pocket. The major interactions are formed by the hydrogen bond between the acid moiety to Phe19 of the N-terminal residue and the hydrophobic interactions between the rest of the compound with the pocket, especially the aromatic ring of the compound which positioned in a hydrophobic “hole” formed by hydrophobic residues Leu43, Val46, Cys301 and Pro305. (See Fig. S5a,b). The quinoline ring of **4–28** lies in a hydrophobic pocket and the acid moiety binds to the Phe19 residue. Modeling suggested modifications to the quinolone moiety, alkyl and acidic chains could be tolerated within the putative binding pocket and thus an exploratory SAR study was undertaken. A small library of analogs of **4–28** was synthesized (Supplemental Information, Table S2) and assessed for inhibitory activity against ATG4B. Compound **4–28** presents a structural motif with a flat 4-styrylquinoline core to which is appended a lipophilic chain and a carboxylic acid bearing chain arrayed on the 4′-amino group. As the conjugation of the amino function to the styryl unit was considered to be largely responsible for the reactivity towards nucleophiles and the photo-instability, we sought to replace or displace the amino group and also to optimize the heterocyclic (quinoline) moiety as well as the two chains. In summary, these studies (Table S2) indicated that the aniline nitrogen was not critical for activity and could be displaced or replaced by a carbon atom and that the two chains could be appended via a dithioacetal or as a mono-thioether. One carboxylic acid function was necessary for activity, with the thiopropionic chain being optimal, and the optimal lipophilic chain was found to be the thioethylphenyl group. The dithioacetal could be placed in the 4′ or 3′ position with similar potency. The quinolone ring was found to be optimal but could be replaced with other bicyclic aromatic or heteroaromatic groups. However, monocyclic analogs (e.g. pyridyl) lost activity. Given that unsubstituted quinolines are known to be potentially toxic through metabolic activation via oxidation^53^, and that such metabolism could impair pharmacokinetics, introduction of quinolone substituents was evaluated and a 7-chloro group was found to have the best profile of those tested. Thus the compound **LV-320** was identified (Fig. 4a) and selected for further profiling. Using the fluorescent peptide substrate assay for ATG4B inhibition^52^, **LV-320** gave an IC_50_ of 24.5 µM (95% CI 20.7 to 29.1 µM) (Fig. 4b).

### LV-320 binds to ATG4B and is an uncompetitive inhibitor of ATG4B

To confirm binding of **LV-320** to ATG4B, a microscale thermophoresis (MST) experiment was conducted and showed that **LV-320** binds to ATG4B with a K_D_ of 16 ± 1 µM (Fig. 4c). Based on our binding pocket model, we predicted that **LV-320** acted as an allosteric inhibitor of ATG4B. To test this, kinetic analyses were carried out for inhibition of ATG4B with **LV-320** using a varying concentration of enzyme and substrate (fluorescent peptide assay). Analysis of data indicated that increasing concentrations of **LV-320** led to suppression of V_max_ while also lowering K_M_, consistent with uncompetitive inhibition (Fig. 4d,e).

### Selectivity on ATG4 isoforms and relevant cysteine proteases

To evaluate selectivity of inhibition, **LV-320** was tested for activity as an inhibitor of the homologous enzyme ATG4A and also the cysteine proteases caspase-3 and cathepsin B. Notably **LV-320** was shown to inhibit ATG4A with an IC_50_ of 35.5 µM (95% CI = 31.4 to 40.2) (Fig. 4f) but did not show meaningful inhibition for caspase-3 (6% inhibition at 60 µM) (Fig. S8a) or cathepsin B (32% inhibition at concentrations up to 60 µM) (Fig. S8b).

### LV-320 reduces autophagic flux *in vitro* and is bioavailable and active *in vivo*

**Figure 5:**
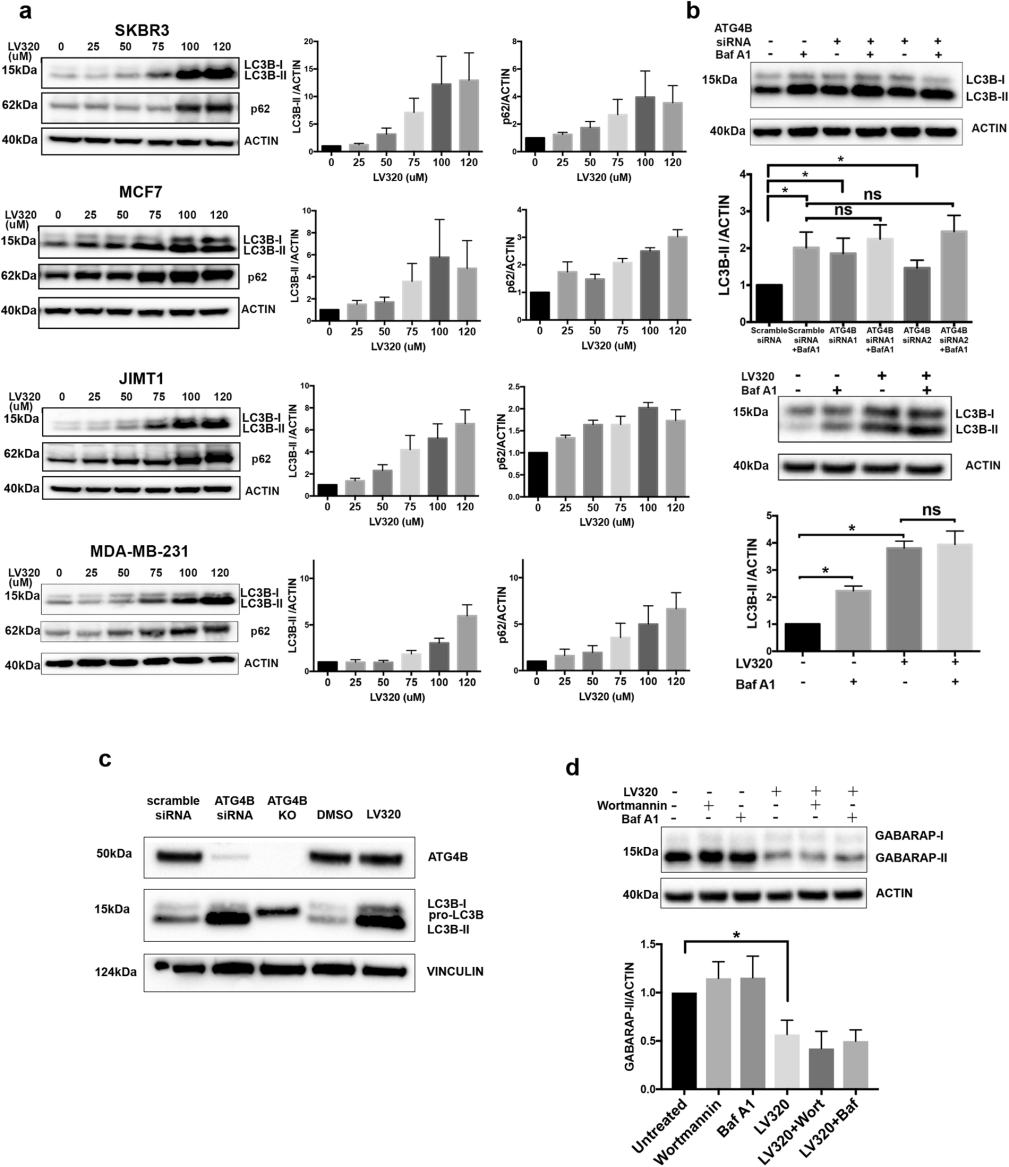
**LV-320** blocks starvation-induced autophagic flux *in vitro*. (**a**) **LV-320** treatment of SKBR3, MCF7, JIMT1, and MDA-MB-231 cells results in accumulation of LC3B-II and p62 in a dose dependent manner. Corresponding mean LC3B-II/actin values were determined using densitometry analysis; error bars, SEM (n = 3). Full-length blots are presented in Fig. S1. (**b**) **LV-320** treatment, similar to ATG4B knockdown, results in inhibition of autophagic flux in MDA-MB-231 cells. Autophagic flux assay using saturating (40 nM) concentrations of bafilomycin A1 (Baf A1) was applied for the assessment of LC3B-II accumulation. Top: The representative western blot shows higher accumulation of LC3B-II following ATG4B siRNA treatment compared to scramble siRNA control; LC3B-II accumulation resulting from ATG4B knockdown is comparable to that from treatment with bafilomycin A1. Bottom: western blot shows higher accumulation of LC3B-II in cells treated with **LV-320** (120 µM for 48 hours) compared to DMSO control; addition of Baf A1 to **LV-320** did not result in further accumulation of LC3B-II. Corresponding mean LC3B-II/actin values were determined using densitometry analysis; n = 3; error bars, SEM; *p < 0.05. Full-length blots are presented in Fig. S1. (**c**) **LV-320** results in accumulation of LC3B-II and not pro-LC3B. Representative western blot shows parental JIMT-1 cells treated with scramble control siRNA (lane 1), ATG4B-siRNA (lane 2), vehicle control (lane 4), or **LV-320** (75 µM; 24 h; lane 5). The control ATG4B-KO JIMT-1 cells showing the location of pro-LC3B are in lane 3. Samples were run on a 4–12% Bis-tris gradient gel. The banding pattern shown is representative of 3 independent experiments. Full-length blots are presented in Fig. S1. (**d**) Representative western blot shows treatment of MDA-MB-231 cells with **LV-320** (120 µM for 24 h) resulted in reduced levels of GABARAP-II. Addition of bafilomycin A1 (Baf A1; 40 nM) or Wortmannin (1 µM) during the final 4 h of treatment had no effect. The GABARAP-II/actin values were determined using densitometry analysis; n = 3; error bars, SEM; *p < 0.05, Student’s t-test. Full-length blots are presented in Fig. S1.

To determine whether **LV-320** alters autophagy, we first investigated its effects on endogenous LC3B in multiple breast cancer cell lines, SKBR3, MCF7, JIMT1 and MDA-MB-231. Treatment with **LV-320** resulted in a dose-dependent increase in endogenous LC3B-II levels in all four cell lines, with changes first detected at 50 to 75 µM **LV-320** (Fig. 5a). Consistent with a block in autophagy, we also detected a dose-dependent increase in the autophagy cargo adaptor protein p62 (Fig. 5a). To further confirm that the increase in LC3B-II represented a block in autophagy flux, the levels of endogenous LC3B-II were quantitated in the presence and absence of bafilomycin A1 (Baf A1). Similar to ATG4B-siRNA, treatment of cells with **LV-320** led to an increase in LC3B-II, that was not further increased in the presence of Baf A1 (Fig. 5b), indicating that **LV-320** blocks autophagic flux. Since uncleaved pro-LC3B migrates at a similar position to LC3B-II in SDS-PAGE^54^, we used gene editing to create an ATG4B-knockout (KO) JIMT-1 cell line as a positive control for pro-LC3B. Side-by-side comparisons, using a 4–12% gradient gel and all in the JIMT-1 cell line background, showed that the pro-LC3B band detected in ATG4B-KO cells migrated at a position intermediate to the LC3B-I and LC3B-II bands detected in both the **LV-320** and ATG4B-siRNA treated cells (Fig. 5c). This observation confirms that **LV-320**, like ATG4B-siRNA, leads to an accumulation of LC3B-II but not pro-LC3B. Reduced levels of GABARAP, an alternate ATG4B substrate, were observed in ATG4B knockout cells^27,55^. We tested effects of **LV-320** on levels of endogenous GABARAP, and found that **LV-320** resulted in a significant decrease in GABARAP levels (Fig. 5d). These observations further support the on-target effects of **LV-320** in cells.

**Figure 6:**
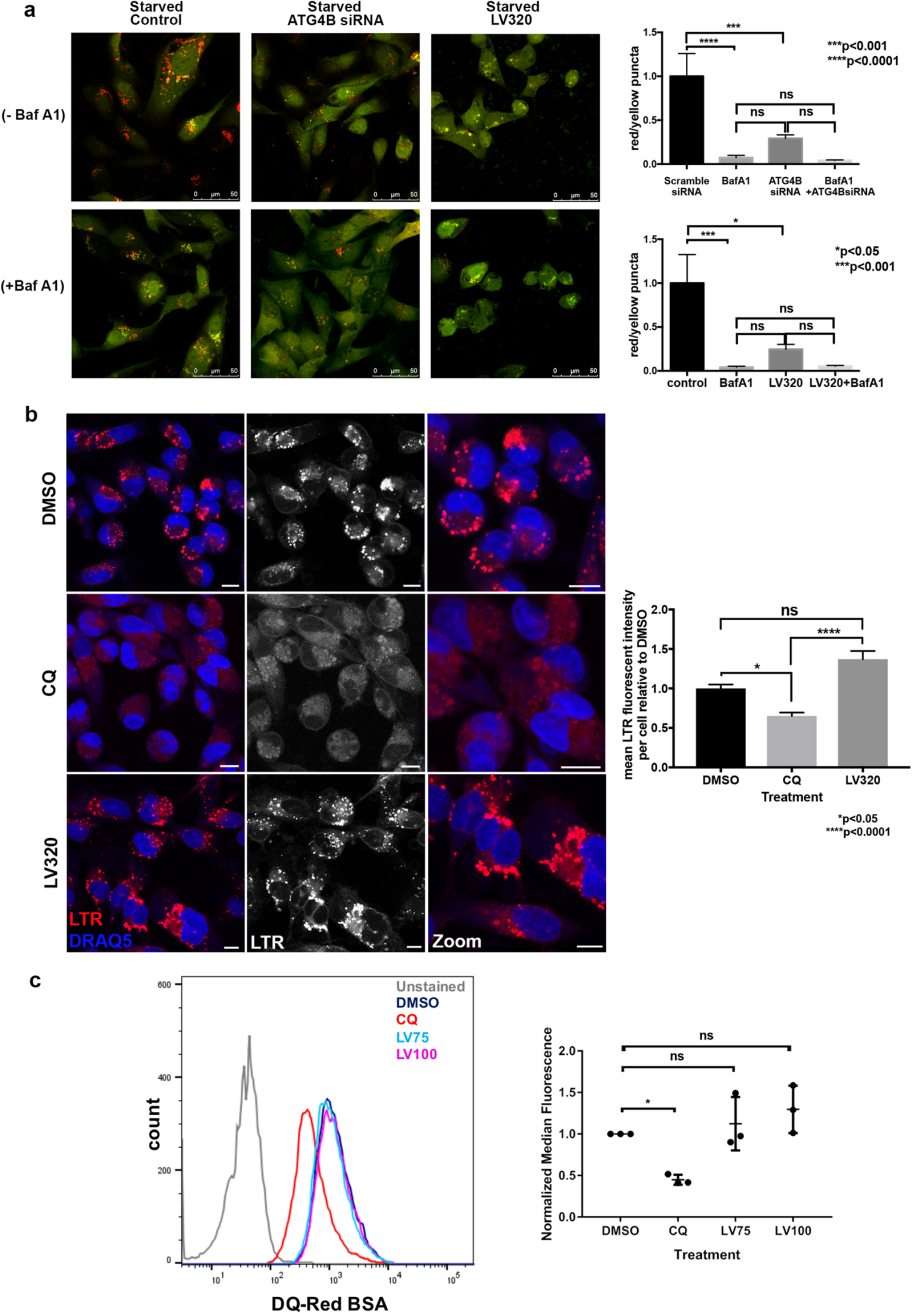
**LV-320** blocks starvation-induced autophagic flux *in vitro* and does not act like CQ. (**a**) **LV-320** treatment, similar to ATG4B knockdown, inhibits autophagic flux. MDA-MB-231 cells stably expressing mRFP-EGFP-LC3B protein were treated with either ATG4B-siRNA or scramble-siRNA, as well as either DMSO or LV-320 (120 µM for 48 hours), with and without bafilomycin A1, under starved conditions. Decrease in red puncta (autolysosomes) relative to yellow puncta (autophagosomes) indicates decreased autophagic flux in response to treatment. Bar graphs show average ratio (mean ± SEM) of red to yellow puncta per cell, n = 3; *P* values are based on the one-way ANOVA with Dunnett post-test. Scale bar, 50 µm. (**b**) MDA-MB-231 cells were treated with DMSO (vehicle control), CQ (40 μm) or **LV-320** (120 μM) for 24 hours before being stained with Lysotracker Red® (LTR) and DRAQ5. Mean levels of gray intensity were measured from the LTR channel per image, made relative to number of cells and normalized to the DMSO control. Bar graph shows mean LTR intensity per cell per treatment (mean ± SEM). At least 200 cells were analyzed per treatment in each of 2 independent experiments; P values are based on the Kruskal-Wallis test with Dunnetts post-test, *p < 0.05, ****p < 0.0001. Scale bar, 10 μm. (**c**) **LV-320** does not inhibit lysosomal degradation. Fluorescence intensity of SKBR3 cells treated with DQ-Red BSA (10 µg/ml) in combination with either DMSO (vehicle control; dark blue), CQ (20 µM; red), **LV-320** (75 µM; light blue) or LV-320 (100 µM; pink). Unstained control cells are shown in gray. Histogram is representative of 3 independent biological replicates, which are shown in the scatter plot; *p < 0.05, *P* values are based on one-way ANOVA with Dunnett post-test.

In an alternate assay to investigate the effect of **LV-320** on starvation-induced autophagic flux, we used stably transfected RFP-GFP-LC3B cells. Since GFP fluorescence is quenched in acid environments, but red fluorescence is preferentially retained, autolysosomes are indicated by red puncta whereas autophagosomes are indicated by yellow puncta, or the overlap of both red and green fluorescence^56^. In starved control scramble-siRNA treated cells, abundant red puncta were detected as expected, indicating high levels of autophagic flux (Fig. 6a). Similar to the positive controls ATG4B-siRNA and the lysosomal inhibitor Baf A1, the formation of red puncta was significantly blocked in **LV-320**-treated cells (Fig. 6a). Together, these results indicate that **LV-320** suppresses autophagic flux *in vitro*. To test whether **LV-320** was acting like a lysosomal inhibitor, we conducted a fluorescence assay using the acidotropic dye Lysotracker Red (LTR). Relative to the vehicle control (DMSO) treated cells, the lysosomal inhibitor CQ resulted in a statistically significant decrease in LTR fluorescence intensity, but **LV-320** had no significant effect (Fig. 6b). This result indicates that, unlike CQ, **LV-320** does not alter lysosomal pH. To determine if **LV-320** affects lysosomal degradation, we performed a DQ-BSA assay. DQ-Red BSA is a self-quenched fluorescent substrate that enters the cell through endocytosis and fluoresces upon lysosomal degradation^57^. Treatment of cells with CQ significantly reduced DQ-Red BSA fluorescence, but DMSO or **LV-320** had no significant effect (Fig. 6c). Together, these results indicate that **LV-320** does not act similarly to the lysosomal compound CQ.

**Figure 7:**
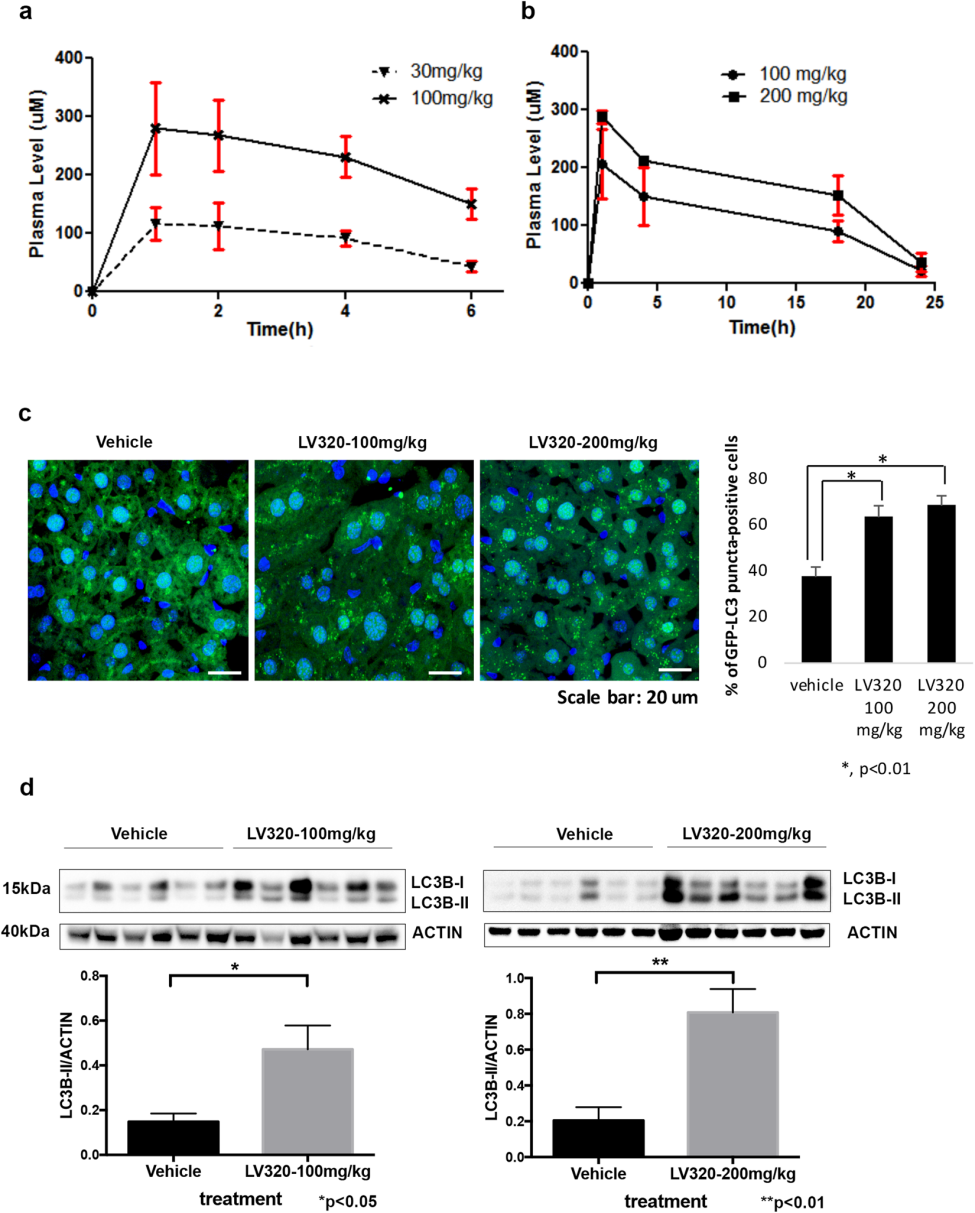
**LV-320** is bioavailable and affects LC3B levels *in vivo*. (**a**) Plasma levels of **LV-320** in BL/6 mice (n = 3, serial samples) after oral doses of 30 or 100 mg/kg; error bars, SD (**b**) Plasma levels of **LV-320** in BL/6 mice (n = 3, serial samples) after oral doses of 100 or 200 mg/kg; error bars, SD (**c**) **LV-320** treatment results in accumulation of GFP-LC3 puncta in mouse liver tissues. Representative images from each treatment condition are shown in the left panel. Scale bar, 20 µm. Quantitative data are shown in the right panel. The percentage of GFP-LC3 puncta positive cells from 5 fields were calculated for each sample; and 6 samples from each treatment condition were grouped; *P < 0.01 (Student’s two-tailed *t*-test). (**d**) **LV-320** treatment results in accumulation of LC3B-II in a dose-dependent manner. Corresponding mean LC3B-II/actin values (per treatment group) were determined using densitometry analysis; error bars, SEM. *p < 0.05, **p < 0.01, Student’s t-test. Full-length blots are presented in Fig. S1.

The pharmacokinetic parameters and tolerability of **LV-320** were assessed in mice with an oral administration of 30, 100 and 200 mg/kg (Fig. 7a,b). **LV-320** displayed a favorable pharmacokinetic profile with a good oral absorption and blood levels were dose proportional up to 100 mg/kg but somewhat less so at 200 mg/kg. The maximum concentration of 210 ± 50 µM and 280 ± 20 µM was achieved after 1 h after 100 and 200 mg/kg doses respectively (Fig. 7b).

To investigate whether **LV-320** affects LC3B levels *in vivo*, we treated GFP-LC3 mice with vehicle solution or **LV-320** at 100 mg/kg or 200 mg/kg dosed three times over two days, and 4 hours after the final treatment we analyzed mouse plasma and livers for drug levels and for the expression of GFP-LC3 puncta using confocal microscopy, and for LC3B protein levels on western blot. Terminal blood levels of **LV-320** (4 h after last dose) were 169 ± 23 µM and liver levels were 104 ± 26 µM. We quantified the expression of GFP-LC3 puncta and found a significantly greater accumulation in **LV-320** treated animals compared to controls (Fig. 7c). LC3B-II protein was also increased in **LV-320**-treated animals (Fig. 7d) compared to vehicle controls, similar to the effects on LC3B-II observed following **LV-320** treatment of cells *in vitro*. The described treatment regimens did not cause significant toxicity in mice at either dose.

## Discussion

In this study, we used a combination of molecular modeling, *in silico* screening and both cell-free and cell-based activity assays to identify five candidate small molecule inhibitors of ATG4B. Four of the compounds (**1–4**, **2–22**, **3–22**, **4–6**) resulted in an increase in GFP-LC3 puncta, and one compound (**4–28**) resulted in a decrease in GFP-LC3 puncta in our primary screen. Of these, the styrylquinoline **4–28** was selected due to its “drug-like” structure. Because **4–28** has potential fluorescent quenching properties, which may have contributed to the observed GFP-LC3 puncta effects, we validated this hit with further non-fluorescence-based autophagy assays in addition to the enzymatic assays. These studies together supported the conclusion that **4–28** reduces ATG4B activity and LC3B-II recycling. However **4–28** is a member of a class of molecules (4-(4′-amino)styrylquinolines) known to be highly photochemically reactive^51^ and indeed we noted its propensity for photo-isomerization and also reactivity towards nucleophiles such as the reducing agent, TCEP, used in our assay protocol to activate ATG4B. SAR studies on **4–28** revealed that the 4′-amino function could be displaced or replaced with a carbon atom without affecting activity. These studies also revealed a rather flat structure-activity relationship but that for potent activity, the styryl double bond was required and two chains, one bearing a carboxylic acid group and another a lipophilic chain, terminated with a phenyl group were optimal.

Based on our SAR study to improve the stability and the potency of **4–28**, compound **LV-320** was identified as one of our most active ATG4B inhibitors, a compound that showed enhanced potency, excellent pharmacokinetics and was found to be well absorbed and tolerated in mice after oral dosing. **LV-320** shows enzyme inhibition kinetics consistent with uncompetitive inhibition of ATG4B, consistent with its predicted binding to the allosteric site closed#2 (Fig. S9) and also inhibits its closely related family member ATG4A with similar potency but does not (significantly) inhibit the cysteine proteases caspase-3 or cathepsin B. Microscale thermophoresis experiments indicated that **LV-320** binds to purified activated ATG4B in solution and molecular docking suggests it may bind at an ancillary site either near the regulatory loop or at the hinge of the N-terminal chain that covers the active site when closed and reveals the site when in the open conformation. The observation that **LV-320** inhibits ATG4A with similar potency to ATG4B would be in keeping with interaction with the putative binding site given that the amino-acid sequences at these sites are largely conserved in the two enzymes (Fig. S10).

Other putative inhibitors of ATG4B have been reported in the literature including some identified through design^36^ and high throughput screening^37–39,52,58^ but these compounds are generally either complex polyphenolic compounds^52,58^ or compounds that work through irreversible alkylation of the active site cysteine^36–39^. Such structures are prone to metabolic instability and not readily optimized to impart “drug-like” properties of stability and reliable pharmacokinetic properties. In addition polyphenolic inhibitors such as aurintricarboxylic acid^36^ are known to be promiscuous inhibitors that are found active in many assays and may be considered as Pan-Assay Interference **(**PAINS) compounds^59^. Akin has reported that NSC 185058 (*N*-pyridin-2-ylpyridine-2-carbothioamide), a compound initially identified through *in silico* screening, inhibited ATG4B activity *in vitro* (with an IC_50_ of 51 µM) as well as inhibiting activation and lipidation of LC3 in cells^40^. The compound also showed ability to inhibit tumor growth in a cancer osteosarcoma xenograft mouse model when dosed daily at 100 mg/kg. However, the authors did not report on the selectivity of NSC185058 nor its pharmacokinetic profile in these treated mice so it is difficult to fully ascribe the beneficial effects to inhibition of ATG4B. While the antitumor effects of NSC 185058 were encouraging, in our hands, we could not demonstrate significant inhibition of purified ATG4B catalytic activity in our assays with NSC 185058 at concentrations up to 200 µM (23% inhibition at 200 µM in the fluorescent peptide substrate assay^52^ and maximum of 16% inhibition at 200 µM in the assay using FRET-LC3 as substrate^36^ whether monitored by fluorescence or by mass spectrometry) (Fig. S11). This discrepancy may be attributed to different assay conditions, compound synthesis and/or reporter constructs used in the two studies. A recent report described several benzotropolone compounds that inhibited recombinant ATG4B and showed promising *in vivo* effects although the compounds were limited by metabolic stability and *in vitro* kinetics of inhibition and selectivity were not described^60^.

Nonetheless, it would be of interest to assess the combination effects of **LV-320**, an allosteric site inhibitor, with other compounds that target the active site of ATG4B. Kinetic analysis of the inhibition of ATG4B with **LV-320** was consistent with uncompetitive inhibition which suggests it may bind to the enzyme at an allosteric site and also may bind to the enzyme-substrate complex.

Several limitations and future directions for the characterization of **LV-320** remain. We have thus far been unable to obtain an ATG4B-**LV-320** co-crystal structure so the exact nature of **LV-320** binding to ATG4B is yet to be determined. The effects of **LV-320** on LC3B-II accumulation and reduced autophagic flux are consistent with studies in yeast, which showed that deconjugation of ATG8-II by ATG4 was required for the subsequent fusion step^20^, but this mechanism has yet to be determined in mammalian cells. It was reported recently that LC3/GABARAP subfamilies are crucial for autophagosome-lysosome fusion and autophagic flux in mammalian cells^61^. Given the effects of **LV-320** on LC3B-II and GABARAP, this is an alternate or additional mechanism consistent with the observed GFP-LC3 accumulation and suppressed autophagic flux following **LV-320** treatment. Moreover, it will be valuable to investigate the possible effects of **LV-320** on the levels and post-translational modifications of all six LC3/GABARAP family members. While we found that **LV-320** did not significantly inhibit the cysteine proteases caspase-3 and cathepsin B, we cannot rule out the possibility of effects on other proteins, and it will be beneficial to conduct more extensive off-target screening. Lastly, **LV-320** remains to be characterized in a variety of cell and tissue types, and in models of disease *in vivo*. Based on studies to date^31–35^, we expect that context dependency will be an important consideration.

*In vitro*, **LV-320** suppressed basal and starvation-induced autophagy flux, and *in vivo* **LV-320** demonstrated a good PK profile and modulation of the ATG4B substrate LC3B. The effects of **LV-320** on LC3B-II accumulation, but not pro-LC3B accumulation, are consistent with reduced LC3B-II delipidation and similar to other observations^22–24^ and our own using ATG4B-siRNAs. Previous studies showed that LC3B-II can be delipidated by ATG4B but not by ATG4A^62^ nor ATG4D^63^. ATG4C delipidation activity on LC3B-II is still unknown, but these data support a requirement for ATG4B in LC3B-II delipidation. The ATG4B knockout phenotype for LC3B is distinct, in that it results in failed conversion or “priming” of pro-LC3B, leading to pro-LC3B accumulation and the loss of detectable LC3B-II. Using a reconstituted cell-free system, Kauffman *et al*. showed that ATG4B priming activity is orders of magnitude faster than its delipidation activity^64^. These data are consistent with our interpretation that residual or low levels of ATG4B activity, in the case of **LV-320 treatment**, are still sufficient for priming or conversion of pro-LC3B to LC3-I, but insufficient or rate-limiting for efficient delipidation of LC3B-II; while unknown, this may be beneficial in a therapeutic context. It is also difficult to know if the added potency of **LV-320** at ATG4A would contribute to a beneficial *in vivo* profile or not. Only when we have a suite of pan- and highly subtype selective-inhibitors of ATG4s will this become clear. The combined results show that **LV-320** is an inhibitor of ATG4B and ATG4A that demonstrates *in vitro*, cellular and *in vivo* effects in keeping with its potency and exposure data. Thus **LV-320** is expected to serve a useful role as a molecular probe to better understand the roles and the therapeutic potential of ATG4 in health and disease.

## Methods

### ATG4B crystal structures and small molecule databases

Code and data availability: The crystal structures of ATG4B were downloaded from the Protein DataBank (PDB) (www.rcsb.org). The NCI small molecule database and the Chembridge database were downloaded from ZINC (zinc.docking.org). We used all free form structures available (PDB entries 2CY7 and 2D1I) as inactive conformations and the structure from one ATG4B-LC3 complex (entry 2ZZP) as the active conformation. The ATG4B in 2ZZP has a single mutation Cys74Ser. A counter mutation S74C was modeled using ICM (Version 3.6, licensed from Molsoft LLC) to generate the wild type structure.

### Pocket Identification, Molecular Docking and Computational Screening

A linux-based computer cluster of 1000 CPUs with ICM package installed was used to conduct the tasks. PocketFinder^45^ was applied to those three crystal structures for identifying small molecule-binding pockets. For molecular docking, each binding pocket is represented by a set of maps for van der Waals (carbon-based and hydrogen-based), electrostatic, hydrogen bonding, and hydrophobic interactions. Each compound (ligand) of the database was docked to the pockets sequentially, and a score reflecting the quality of the docked complex was assigned to each compound^47^. During the docking, the compound is fully flexible, and both the intramolecular ligand energy and the ligand-receptor (pocket) interaction energy were optimized. The conformational sampling was based on the biased probability Monte Carlo procedure in the internal coordinate space^46^. Due to the nature of Monte Carlo based sampling, we repeated the database screening process five times for each pocket and retained the best score for each compound.

### Fluorimetric ATG4B enzymatic assay

100 μL of immobilized TCEP in deionized water was diluted in 2.50 mL of buffer (50 mM Tris, 35 mM NaCl, pH = 8). The sample was centrifuged at 10,000 × g during 1 min. The supernatant was removed. This operation was done two more times. 50 µL of ATG4B (26.75 µM) frozen in buffer (50 mM Tris, 500 mM NaCl, pH 8) was flash thawed and diluted with 100 µL of washed immobilized TCEP. The solution was incubated on a carousel at 4 °C for 1 h. This solution was then diluted with 2.06 mL of buffer (50 mM Tris, 35 mM NaCl, 5 mM EDTA, pH 8), centrifuged at 10,000 × g during 1 min and the supernatant, containing the activated ATG4B (1 µM), was kept. A 96-well all-black plate was loaded where each well was charged with 55 µL of inhibitor at different concentrations (0 µM to 100 µM or 200 µM) in buffer/DMSO 41:3 (v/v) and 20 μL of enzyme or buffer were dispensed using a dispenser (Biotek). Plate was incubated for 30 min. A single read was done using excitation and emission wavelengths of 350/450 nm. 25 µL of peptide substrate (pim-FG-PABA-AMC)^52^ (final assay concentration 100 µM) was dispensed using a dispenser (Biotek). Experiments were performed in triplicate except when it is mentioned. Fluorescent reads were performed using the Synergy 4 (Biotek) at 25 °C using excitation and emission wavelengths of 350/450 nm every 15 min for 2 h.

### Enzyme kinetic analysis using fluorimetric ATG4B enzyme assay

20 µL of ATG4B (292 µM) frozen in buffer (50 mM Tris, 500 mM NaCl, pH 8) was flash thawed, diluted with 1 mL of reducing buffer (50 mM Tris buffer, 35 mM NaCl, 5 mM EDTA, 1.25 mM TCEP, pH 8) (final assay concentration of 5.73 µM) and incubated on ice for 1 h. A 96-well all-black, non-binding surface plate was loaded with 50 µL of ATG4B (final assay concentration 400 nM) and **LV-320** at various concentrations (to provide final assay concentrations of 6 µM to 54 µM) in reducing buffer/DMSO 95:5 (v/v), and incubated at ambient temperature for 30 min. A separate 96-well plate was charged with solutions of fluorescent peptide substrate^52^ at different concentrations (to provide final assay concentrations of 10 µM to 124.4 µM) in reducing buffer/DMSO 95:5 (v/v). A Thermo Scientific Matrix PlateMate 2 × 2 was used to transfer 50 µL from the substrate plate to the enzyme plate and mix the solutions. The plate was transferred to a Synergy 4 Fluorometer (BioTek) and fluorescence was scanned at 30 min, 120 min and 210 min, exciting at 350 nm and measuring emission at 450 nm. Experiments were performed as technical quadruplicates, with representative biological duplication. Raw fluorescence measurements were converted from RFU/s to M/s using a standard curve. Using GraphPad Prism 5 software, the mean and standard error of the replicates were analysed using a Michaelis-Menten model with Least Squares fitting to obtain V_max_ and K_M_ values for each experiment. V_max_ and K_M_ were plotted against [**LV-320**] to qualitatively observe any relationship between the variables.

### Mass spectrometry ATG4B enzymatic assay

20 µL of immobilized TCEP (ThermoFischer # 77712) in deionized water was diluted in 500 µL of buffer (50 mM Tris, 35 mM NaCl, pH = 8). The sample was centrifuged at 10000 × g during 1 min. The supernatant was removed. This operation was done two more times. ATG4B (45 µM) frozen in buffer (50 mM Tris, 500 mM NaCl, pH 8) was flash thawed and diluted to 15 µM with buffer (50 mM Tris, 35 mM NaCl, pH 8). 15 µL of this ATG4B solution were mixed with the 20 µL rinsed immobilized TCEP solution. The solution was incubated on a carousel at 4 °C for 1 h. This solution was then diluted with 1.84 mL of buffer (50 mM Tris, 35 mM NaCl, pH 8), centrifuged at 10000 × g during 1 min and the supernatant, containing the activated ATG4B (120 nM), was kept. Kinetic determination was performed in triplicate in 1 dram HPLC vials using 350 µL glass inserts. The vials were charged with 5 µL of inhibitor at different concentrations, 63.9 µL of buffer, 20 µL of 120 nM activated ATG4B and 11.1 µL of YFP-LC3-GFP (90 µM). Every 10 min during 40 min, 1 µL was injected into a TOF LC/MS (Agilent 2610). The analysis of the m/z distribution signal resulting from the protein substrate was performed by Agilent MassHunter Workstation software elucidating proteins 20000–80000 Da between 700 *m/z* and 1800 *m/z*. Peak intensities were recorded for the parent substrate (72904 Da) and both cleaved peptides (27885 Da and 45036 Da). The amount of cleavage was determined by calculating the value of the formula A/(A + B) where A was the peak height of the 27885 Da residue and B was the peak height of the 72904 Da residue. Each experiment was assessed with a concomitant parallel negative control.

### Microscale Thermophoresis

181 µL of buffer (50 mM Tris, 35 mM NaCl, 1 mM TCEP.HCl, 0.05% Tween 20, pH = 8) were added to the 10 µL-aliquot of ATG4B (191 µM). This solution was kept on fridge for 1 h at 4 °C. ATG4B was labeled using the Monolith NT.115^TM^ Protein Labeling Kit RED NHS (NanoTemper Technologies) according to the supplied protocol. The concentration of labeled ATG4B after preparation, purification, and dilution with buffer (50 mM Tris, 35 mM NaCl, 1 mM TCEP.HCl, 0.05% Tween 20, pH = 8) was at 0.17 µM. **LV-320** was dissolved in a DMSO/buffer (1/9) solution to 800 µM. Compound was titrated in 1:1 dilution with enzyme. Binding assays were performed by microscale thermophoresis with a Monolith NT.115 instrument (NanoTemper Technologies) using 16 standard capillaries with 0.17 µM protein (5% DMSO) at 20% LED power and 80% MST power. The results were analyzed with NanoTemper software to determine K_d_ values.

### Selectivity assays

#### Caspase 3 Assay

Inhibition of caspase 3 was assayed using the Abcam Caspase 3 Inhibitor Drug Detection Kit (ab102491) according to the manufacturer’s protocol.

#### Cathepsin B Assay

The assay was performed essentially as described by Mendieta *et al*.^60^ with Cathepsin B (human, recombinant, active - BioVision #7580-5), Substrate *Z*-Arg-Arg-AMC (Sigma C5429) and 1 µM *Z*-VAD-FMK (Adooq #A12373) as positive inhibition control.

The assay was maintained at 37 °C and monitored every 2 min for 16 min at 348 nm/440 nm (excitation/emission).

#### ATG4A Assay

ATG4A (10 µM final) was reduced for 1 hour at 4 °C in assay buffer (50 mM Tris pH 8, 35 mM NaCl, 5 mM EDTA, 1.25 mM TCEP). Enzyme and inhibitor were preincubated for 30 min at room temperature in assay buffer (5% DMSO final). Fluorescent peptide substrate^52^ (100 µM final) was added to initiate the reaction. The assay (100 µL total volume in 96 well plate, Corning 3650) was maintained at room temperature and monitored every 15 minutes for 2 h at 350 nm/450 nm (excitation/emission).

### Synthesis of analogs

Synthesis of analogs and compound characterization data are provided in the Supplemental Experimental Procedures.

### Reagents

Anti-LC3B (#ab48394, Abcam), anti-β-actin (#ab6276, Abcam), anti-ATG4B (#A2981, Sigma), anti-p62 (#P0067, Sigma), anti-GABARAP (#ab109364, Abcam), anti-vinculin (#ab129002, Abcam), goat anti-mouse IgG–horseradish peroxidase (HRP), and goat anti-rabbit IgG–HRP (Santa Cruz Biotechnology) antibodies were used in immunoblotting. Wortmannin (Sigma-Aldrich) was used to block autophagosome formation and bafilomycin A1 (Sigma-Aldrich) was used for autophagy flux assays.

### Cell lines and culture conditions

MDA-MB-231, MCF7, and SKBR3 cells (American Type Culture Collection, ATCC), authenticated by isoenzyme and short tandem repeat (STR) analyses), as well as JIMT1 cells (German Collection of Microorganisms and Cell Culture (Deutsche Sammlung von Mikroorganismen und Zellkulturen GmbH), DSMZ; authenticated by STR analyses) were maintained in Gibco DMEM (Life Technologies) supplemented with 10% fetal bovine serum (FBS) and confirmed to be mycoplasma negative. SKBR3 cells (ATCC) stably transfected with hrGFP-LC3B^65^ or MDA-MB-231 cells stably transfected with mRFP-eGFP-LC3B were grown in DMEM supplemented with 10% FBS, 20 mM HEPES, and 1X non-essential amino acids (Gibco) with Geneticin (G418). All cells were maintained at 37 °C with 5% CO_2_ and 95% humidity.

### siRNA transfection

For western blot analysis, cells were plated in 6-well plates. For the experiments involving fluorescence microscopy, cells were plated in 8-well chamber slides. The following day, cells were transfected with 75 pmol of either ATG4B siRNA (Fig. S12) or a scramble medium GC siRNA negative control (Invitrogen) using Lipofectamine RNAiMAX^TM^ (Invitrogen) as per manufacturer’s recommendations. Seventy-two hours after the transfection, cells were harvested. For the assessment of autophagic flux, bafilomycin A1 (40 nM) was added to the respective wells five hours before harvesting the cells. For the experiments involving starvation, five hours before harvesting, the medium in respective wells was replaced with Earl’s Balanced Salt Solution (EBSS).

### ATG4B KO cell line

Single-guide RNA (sgRNA) sequences targeting ATG4B were selected from the GeCKO V2 library (Addgene) (ATG4B-1: TCCTGTCGATGAATGCGTTG) and ligated into pSpCas9(BB)-2A-GFP (PX458) (Addgene). JIMT-1 cells were transfected with the resultant CRISPR plasmid. Monoclonal cell lines were established by FACS sorting GFP-positive cells into single-cell culture 48 hours after transfection and verified with western blotting.

### Confocal microscopy

SKBR3 cells stably transfected with hrGFP-LC3B or MDA-MB-231 cells stably transfected with mRFP-eGFP-LC3B were used for monitoring autophagy.

For each experiment, media was replenished every day after initial plating and before treatment. At the end of the experiment the cells were fixed in 4% PFA for 20 minutes and then rinsed with PBS. Cells were then mounted using SlowFade Gold Antifade with DAPI (Invitrogen). For Lysotracker Red^®^(LTR) experiments, parental MDA-MB-231 cells were incubated with 50 µM of LTR for 5 min in the dark CO_2_ incubator, rinsed with PBS and fixed with 4% PFA for 20 min. Fixed cells were incubated in DRAQ5 (1:500) in PBS and 100 µg/mL RNaseA for 10 minutes at room temperature before being mounted with SlowFade Gold Antifade (Invitrogen). Leica TCS SP8 inverted confocal microscope with a Leica HC PL APO 63x/1.40 oil objective and LAS AF software (Leica) were used for imaging the cells. For each treatment, the number of green, red and yellow (i.e. red/green overlap where applicable) puncta per cell were determined for at least 100 cells. Puncta counting was performed using the OpenCV Python package; a contours discovery algorithm^66^ was applied to images pre-processed by filtering colors and applying a Gaussian filter and adaptive threshold. LTR fluorescent intensity was measured by the mean gray value from the unadjusted images taken with the same microscope settings of the LTR channel and subtracting the mean background using Image J 1.45 s (https://imagej.nih.gov/ij/) with cells counted by DRAQ5 positive nuclei.

### DQ-BSA Assay for Lysosomal Activity

A DQ-BSA assay was used to determine lysosomal degradative activity as described^57^. SKBR3 cells were incubated overnight in 10 µg/ml DQ-Red BSA (Thermo Fisher D12051) along with either DMSO (0.5%), 20 µM CQ, 75 µM **LV-320** or 100 µM **LV-320**. All samples contained equal concentration of DMSO (0.5%) in regular growth media (Gibco DMEM + 10% FBS + 20 mM HEPES + 1X NEAA). Cells were analyzed 24 h later for DQ-Red BSA fluorescence using a Fortessa (BD Biosciences) flow cytometer, using Sytox Green (Thermo Fisher S7020) to exclude dead cells. Data were analyzed using FlowJo software.

### High Content Screening assay

High-throughput fluorescence microscopy was performed at room-temperature on the InCell Analyzer 1000 (GE Healthcare) using the 20X objective to view Hoescht 33342 nuclear stain, ethidium homodimer (EthD1) viability stain and GFP-LC3 puncta in SKBR3-hrGFP-LC3B cells. Thirty min prior to imaging, cells were incubated at 37 °C with 4 µM Hoechst 33342 (Invitrogen) and 0.5 µM EthD1 (Invitrogen). Image analysis was performed using InCell Developer Toolbox (Ver. 1.6, GE Healthcare). Individual cells were identified with segmentation of nuclei based on Hoechst 33342 staining. Cell borders were determined by defining a radius around the nucleus. Cells were classified as “dead” if the defined cell overlapped by at least 50% with the EthD1 signal. The number of GFP-LC3 puncta within each live cell was then counted, as well as the number of live cells with at least 5 puncta. Puncta were defined as GFP positive structures of at least 1 pixel with a form factor (estimate of circularity) of at least 0.4.

### Protein extraction and western blot analysis

Frozen cell pellets were lysed using RIPA lysis buffer (Santa-Cruz, ref: SC24948) plus complete protease inhibitor cocktail (Roche, ref: 11836153001). The amount of proteins in cell lysates was determined by BCA protein assay (Thermo Scientific, ref: 23225), and 10 µg of protein were loaded on 10% NuPAGE Bis-Tris gel (Invitrogen) for separation and transferred to PVDF membrane (BioRad). BOLT and NUPAGE Bis-Tris 4–12% gradient gels (Invitrogen) were used for separation of pro-LC3B and LC3B-II. Membranes were blocked in 2% milk and incubated in primary antibodies overnight at 4 °C. Primary antibodies were diluted in 2% milk and included: p62 (1:1000; Sigma, ref: P0067), LC3B (1:1000; Abcam, ref: ab48394), GABARAP (1:1000; Abcam, ref: ab109364), beta-actin (1:10000; Abcam, ref: ab6276), and vinculin (1:1000; Abcam, ref: ab129002). Membranes were then washed with 1X PBS-T (0.1%), incubated in HRP-conjugated secondary antibodies and detected using Bio-Rad Clarity Western ECL substrate and the electronic Bio-Rad ChemiDoc MP System. Densitometry was performed using Image Lab software (Bio-Rad), and the relative levels of LC3B-II were determined by first normalizing to the actin loading controls then to the DMSO controls.

### Animal studies

Animal protocols were reviewed and approved by the Institutional Animal Care Committee (IACC) at the University of British Columbia (Vancouver, BC, Canada) and at Simon Fraser University before conducting experiments. The care, housing, and use of animals were performed in accordance with the Canadian Council on Animal Care Guidelines.

### Pharmacokinetic assay

**LV-320** was dissolved in DMSO/Kolliphore/EtOH/methylcellulose/ammonia/water 6.67:1.33:5:1:0.09:85.91 to give a concentration of 10 mg/mL. Three BL/6 female mice (age 12 weeks) were administered **LV-320** at appropriate concentration in vehicle at 5 ml/kg per os. Blood samples (approximately 50 µL) were collected at predetermined intervals. Blood samples were placed into tubes containing sodium heparin and centrifuged at 10000 × g for 14 min to obtain the plasma samples. Concentrations of **LV-320** in plasma were determined (non-blinded) using HPLC analysis after a 1:1 dilution with CH_3_CN and centrifugation at 14000 × g for 5 min.

### *In vivo* autophagy assay

GFP–LC3 mice were kindly provided by Dr. Mizushima (Tokyo Medical and Dental University, Japan)^67^. The animals (females, 9–14 weeks) were allocated into three groups (n = 6 per group with equivalent average body weights) to receive a treatment with vehicle solution or **LV-320** at the dose of 100 mg/kg or 200 mg/kg twice a day, for a total of 3 doses. The treatment was given by oral gavage. Four hours after the last dose the mice were terminated, blood samples were collected and the livers were harvested for the analysis.

### Assessment of GFP–LC3 puncta *in vivo*

After treatment, mouse liver tissues were collected and fixed with 4% paraformaldehyde dissolved in 0.1 M Na-phosphate buffer (pH 7.4) for 4 h, incubated overnight with 15% sucrose/PBS, and 30% sucrose/PBS for 4 h. Tissues were then embedded in OCT compound (Tissue-Tek) and stored at −80  °C. Liver OCT blocks were sectioned into 6 μm slides and countered stained with DAPI-containing mounting medium. Five randomly selected fields from each sample were examined (scorer was blinded) under 40x oil lens of the confocal microscope. Z-stack images were taken from each field with fixed settings at the laser power and exposure time for all samples. Green for GFP-LC3 and blue for nucleus (DAPI). The percentage of GFP-LC3 puncta positive cells from 5 fields were calculated for each sample; and 6 samples from each treatment condition were grouped for statistical analysis. Six animals per treatment group were used based on results in previous studies validating this method as a way to quantify GFP-LC3 puncta^67^.

### Statistical analyses

For statistical analyses, a one-way ANOVA with a Dunnett’s post test was performed using GraphPad Prism version 7.0 for Windows (GraphPad Software, San Diego California USA, www.graphpad.com) unless stated otherwise. Differences between means were considered significant if the resulting p-value was less than 0.05.

### Data availability

The datasets generated during and/or analysed during the current study are available from the corresponding authors on reasonable request.

## Electronic supplementary material

Supplementary Information
