# Supplementary material for: A new quinoline-based chemical probe inhibits the autophagy-related cysteine protease ATG4B
Source: Sci Rep. 2018 Aug 3;8:11653. doi: 10.1038/s41598-018-29900-x (PMC6076261; doi:10.1038/s41598-018-29900-x)
Supplement: Supplementary file 1 — Supplementary Information [file 41598_2018_29900_MOESM1_ESM.pdf]

SUPPLEMENTAL INFORMATION

A new quinoline-based chemical probe inhibits the autophagy-related cysteine protease ATG4B.

AUTHORS/AFFILIATIONS

Bosc D [1,#], Vezenkov L [1,‡], Bortnik S [2,3], An J [2], Xu J [2,4], Choutka C [2,4], Hannigan AM [2], Kovacic S [1], Loo S [1], Clark, PGK [1], Chen G [1], Guay-Ross RN [1], Yang K [2,4], Dragowska WH [5], Zhang F [6], Go NE [2], Leung A [2], Honson NS [7], Pfeifer TA [7], Gleave M [6], Bally M [5], Jones SJ [2,3,4], Gorski SM [2,3,4,*], Young RN [1,*]

[1]Department of Chemistry, Simon Fraser University, Burnaby, BC, V5A 1S6, Canada

[2]Canada's Michael Smith Genome Sciences Centre, BC Cancer Agency, Vancouver, BC, V5Z 4E6, Canada

[3]Interdisciplinary Oncology Program, University of British Columbia

[4]Department of Molecular Biology and Biochemistry, Simon Fraser University, Burnaby, BC, V5A 1S6, Canada

[5]Experimental Therapeutics, BC Cancer Agency, Vancouver, BC, V5Z 4E6, Canada

[6]Department of Urologic Sciences and Vancouver Prostate Centre, University of British Columbia, Vancouver, BC, V6H 3Z6, Canada

[7]Centre for Drug Research and Development, 2405 Wesbrook Mall – 4th Floor, Vancouver, BC, V6T 1Z3, Canada

[#]Present address: INSERM, Institut Pasteur de Lille, U1177 Drugs & Molecules for Living Systems, Université de Lille, F-59000 Lille (France).

SUPPLEMENTAL INFORMATION

‡Present address: Institut des Biomolécules Max Mousseron (IBMM), UMR 5247 CNRS, Université de Montpellier, ENSCM, Faculté de Pharmacie, 15 avenue Charles Flahault, 34093, Montpellier (France).

*Correspondence: robert_young@sfu.ca and sgorski@bcgsc.ca

TABLE OF CONTENTS :

	Page
**1. Protein preparation pocket identification and in silico screening**	3
**2. Figure S1. Full-length western blots.**	4
**3. Figure S2.** Binding model of compound **1-4**	9
**4. Figure S3.** Binding model of compound **2-22** (blue stick model) and **3-22**	10
**5. Figure S4**. Binding model of compound **4-6**	11
**6. Figure S5.** ATG4B active conformation with compound **4-28** bound	12
**7. Figure S6**. Instability of compound **4-28** under UV irradiation	13
**8. Figure S7**. Integrity of compound **4-28** in the fluorimeter as analyzed by HPLC	13
**9. Figure S8.** Inhibition curve for **LV-320** with caspase 3 and cathepsin B	14
**10. Figure S9.** Representation of **LV-320** binding to the putative binding site on ATG4B	14
**11. Figure S10.** Sequence alignment of ATG4A and ATG4B	15
**12. Figure S11.** Inhibition of ATG4B activity observed for NSC185058	16
**13. Figure S12.** Sequences of siRNA duplexes	16
**14. Table S1.** Compound hits from NCI library in silico screen	17
**15. Table S2.** ATG4B Inhibitory activity of synthesized compounds	18
**16. Synthesis, characterization of compounds and spectra**	20

PROTEIN PREPARATION, POCKET IDENTIFICATION AND IN SILICO SCREENING:

The crystal structures of ATG4B were obtained from the RCSB Protein Data Bank (PDB, http://www.rcsb.org/pdb/). ICM package (version 3.6) was used for protein preparation, pocket identification and molecular docking-based screening of small molecule databases. The structure data (sdf files) of small molecules were downloaded from ZINC (http://zinc.docking.org).

The protein preparation tools implemented in ICM [1] were used to prepare the proteins for docking. Water molecules, ligands and ions were removed. Counter mutations were modeled for PDBs with mutated amino acids (catalytic residues). Hydrogens were added and global optimization was performed to find the best hydrogen bonding network. In addition, the orientations of His, Pro, Asn, Gln, Cys were optimized. The protonation states of His were also optimized.

The PocketFinder [2] was applied to those crystal structures for identifying small molecule-binding pockets. This method uses only the protein structure for the prediction of cavities and clefts. The position and size of the ligand-binding pocket are determined based on a transformation of the Lennard-Jones potential by convolution with a Gaussian kernel of a certain size, a grid map of a binding potential and construction of equipotential surfaces along the maps.

A set of grid maps was pre-calculated for each pocket. The maps represent hydrogen bonding potential, van der Waals potential, hydrophobic potential and electrostatic potential. The maps were generated in a rectangular box with 0.5 Å grid spacing centered at the predicted small molecule binding site.

The docking used ICM's flexible-ligand and rigid-receptor protocol. ICM ligand docking is based on biased probability Monte Carlo optimization of the ligand coordinates in the grid maps of the receptor [3]. Each small molecule is first converted to 3D and optimized using MMFF-94 force field. Then the molecule is submitted to a conformational analysis outside of

the protein pocket and a stack of low energy conformations is collected and used as starting geometries for the docking. In the docking, the ligand is placed in the binding pocket and Monte Carlo simulations and global energy optimization were conducted. The energy function includes the ligand internal strain and a weighted sum of the value from the grid maps. The top 4 best scoring poses for each ligand were kept for subsequent scoring. The ICM scoring function evaluates the full-atom complex of the selected pose and the receptor. It was derived from a benchmark dataset of experimentally determined ligand-receptor complexes. The binding score (ICM score) was calculated as a sum of weighted terms of van der Waals, electrostatic, hydrogen bonding, non-polar and polar atom solvation energy differences between bound and unbound states, ligand internal strain and its conformational entropy loss upon binding [4].

FIGURES

Figure S1. Full-length blots are shown next to corresponding cropped images.

SUPPLEMENTAL INFORMATION

Figure 2B

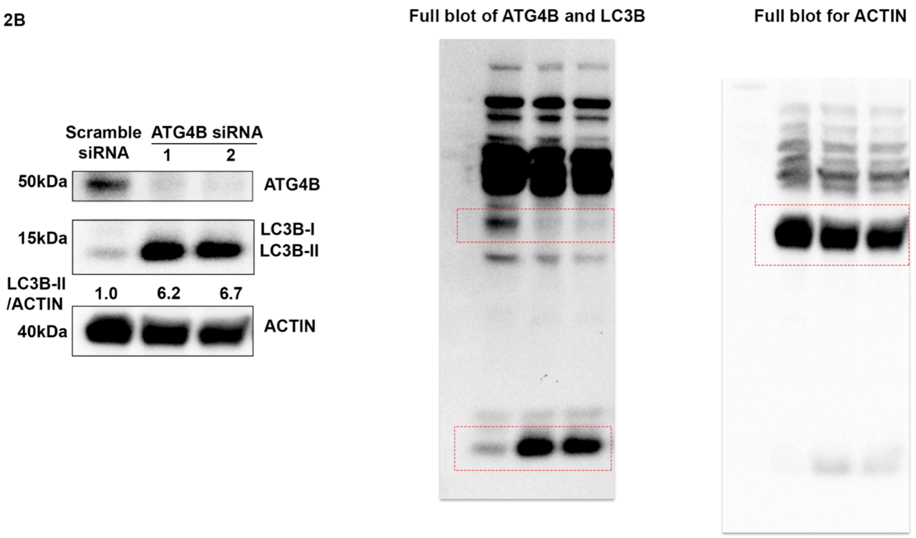

Figure 3 E

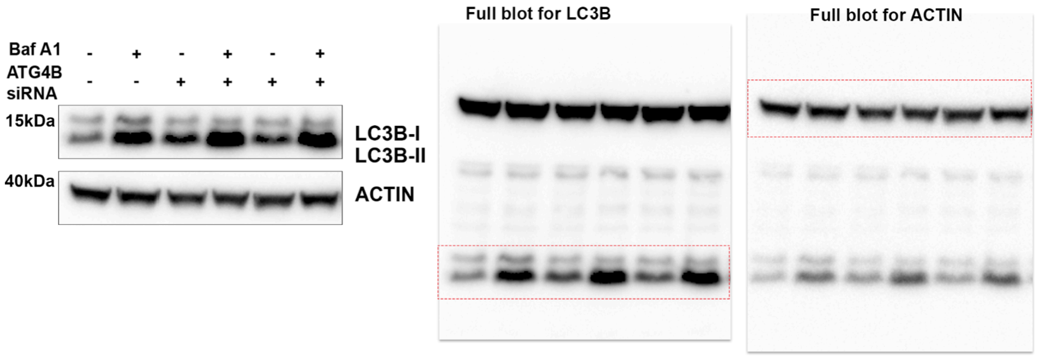

Figure 3 F

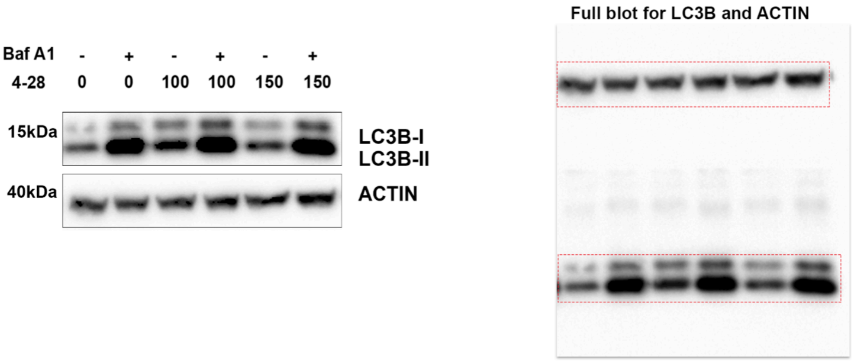

Figure 5a

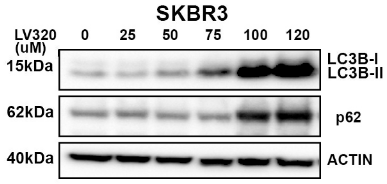

Full blot for LC3B and p62

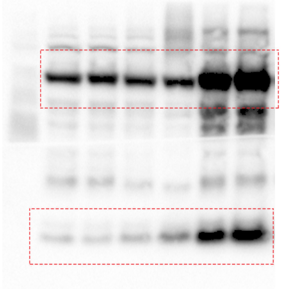

Full blot for ACTIN

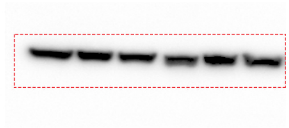

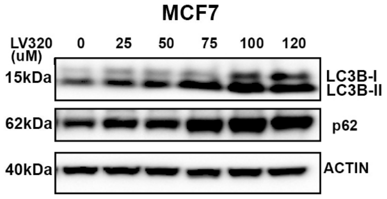

Full blot for LC3B and p62

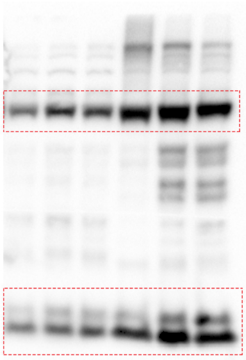

Full blot for ACTIN

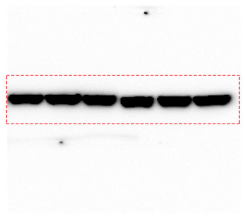

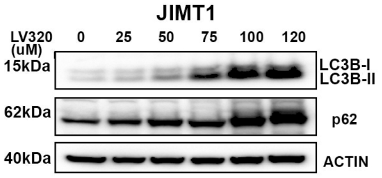

Full blot for LC3B and p62

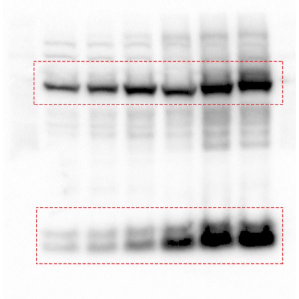

Full blot for ACTIN

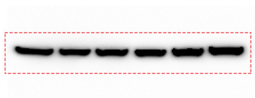

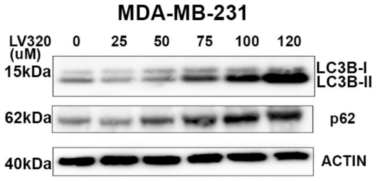

Full blot for LC3B and p62

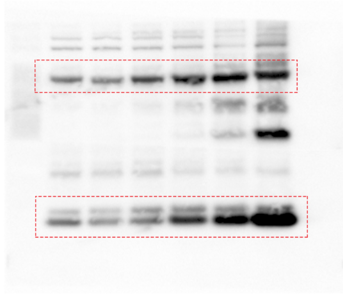

Full blot for ACTIN

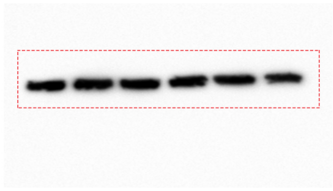

Figure 5b

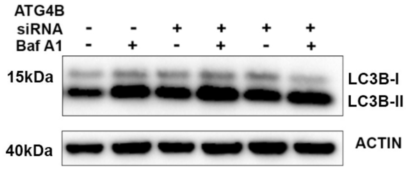

Full blot for ACTIN and LC3B

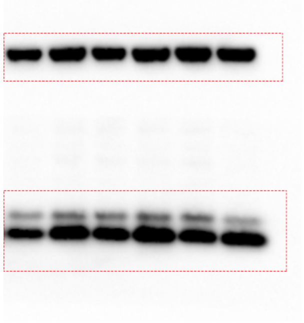

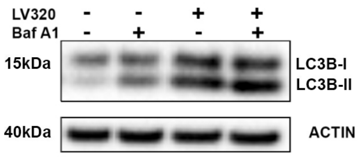

Full blot for LC3B

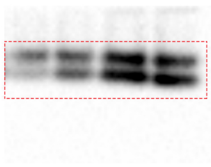

Full blot for ACTIN

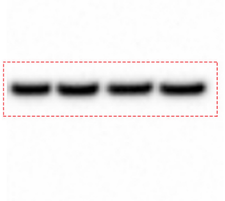

Figure 5c

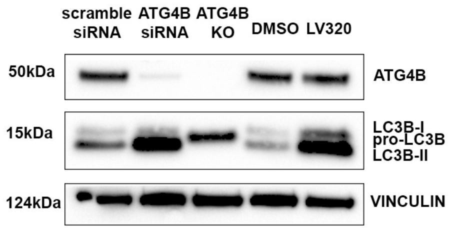

Full blot for VINCULIN, ATG4B, and LC3B

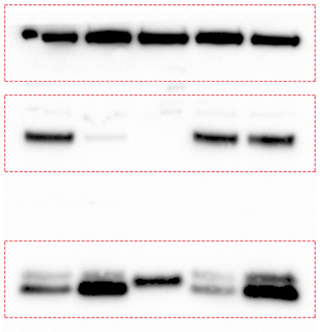

Figure 5d

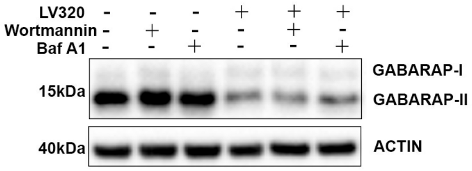

Full blot for GABARAP

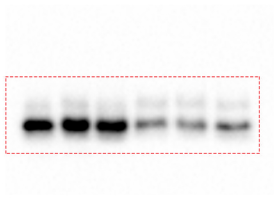

Full blot for ACTIN

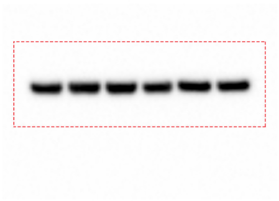

Figure 7D

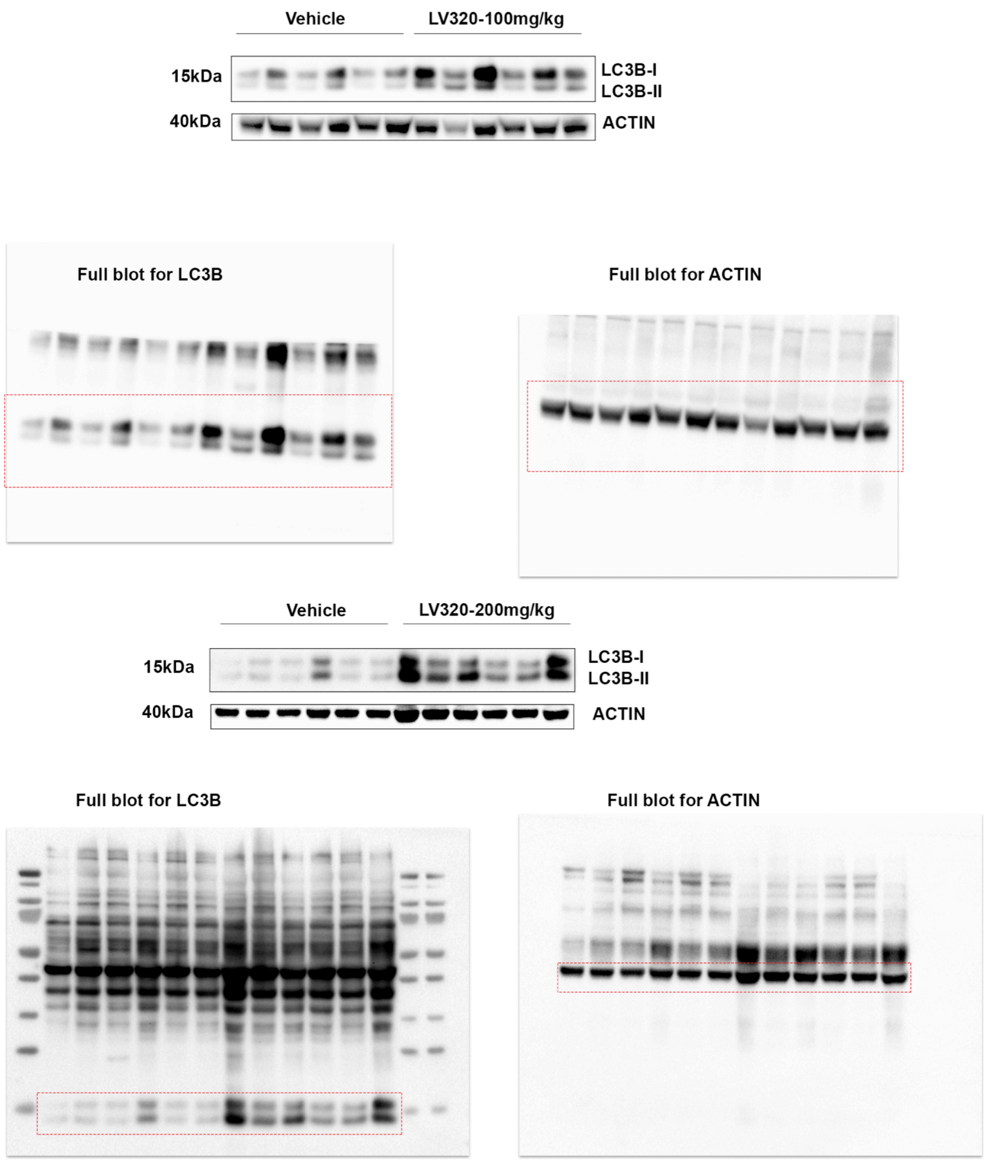
Vehicle
LV320-100mg/kg
15kDa
LC3B-I
LC3B-II
40kDa
ACTIN
Full blot for LC3B
Full blot for ACTIN
Vehicle
LV320-200mg/kg
15kDa
LC3B-I
LC3B-II
40kDa
ACTIN
Full blot for LC3B
Full blot for ACTIN

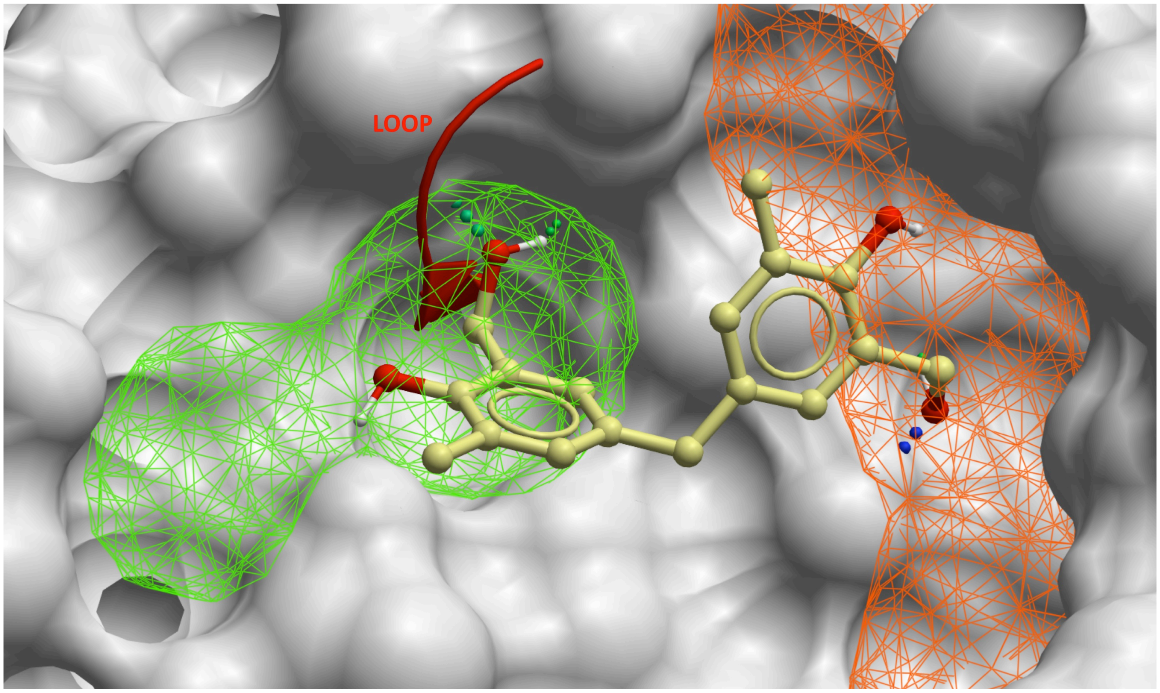

Figure S2. Binding model of compound **1-4** which binds to pocket closed#1. The predicted pocket (green) is on the back of the flexible loop (red ribbon). Another adjacent site indicated in orange. The compound binds to the ATG4B protein across the two pockets. The closed conformation of ATG4B is in grey skin model and the open (active) conformation is in red ribbon model.

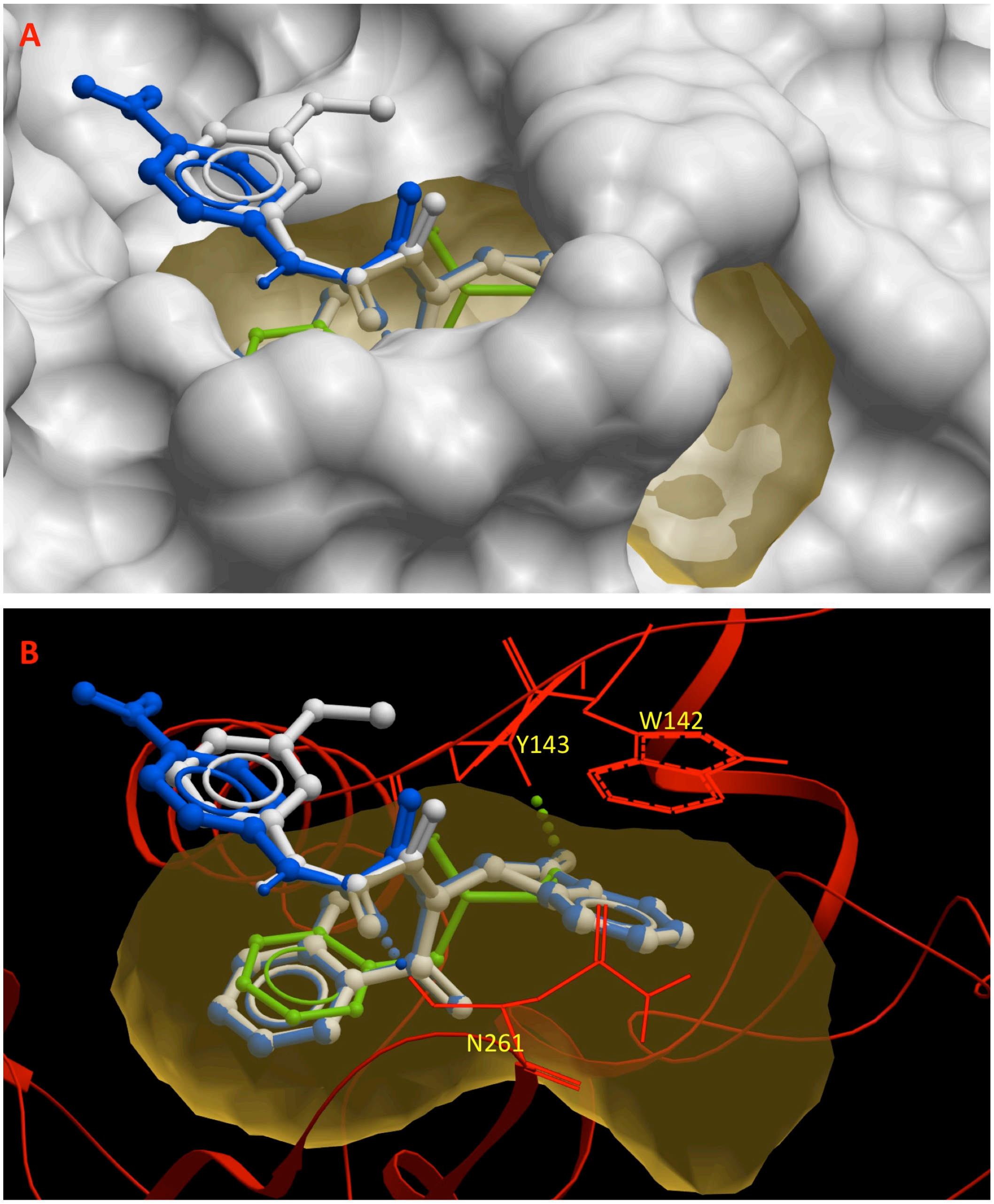

Figure S3. Binding model of compound **2-22** (blue stick model) and **3-22** (white) which are both predicted to bind to pocket open#1. These two compounds are very similar. They bind to the active site (yellow pocket) of the open conformation of ATG4B. A) Skin model shows the compounds bind into the cavity under the “bridge”. B) Ribbon model (red) of ATG4B and the key residues of interaction. Hydrogen bonds are formed with Y143 and N261. Pi-stacking is formed with W142. Part of the compounds mimics the Phe119 (green) of LC3 which is a key residue to lift the active site.

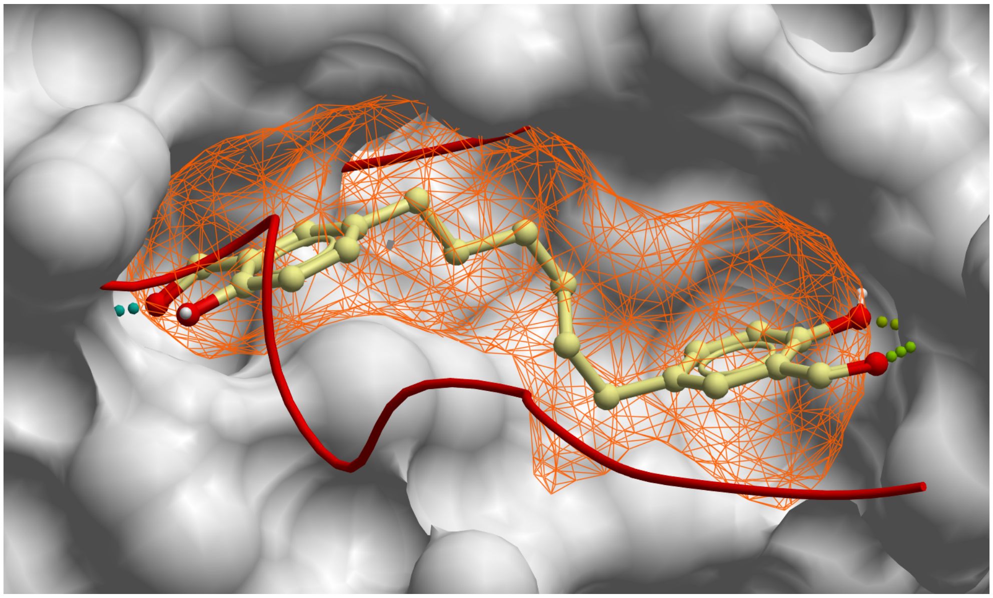

Figure S4. Binding model of compound **4-6** predicted to bind to pocket closed#2. This compound binds to the same pocket as **4-28**. Hydrogen bonds are formed at left side with F19 and the right side with Q100. The compound lies completely within the predicted pocket (orange chicken wire presentation). The ATG4B protein is in gray skin model.

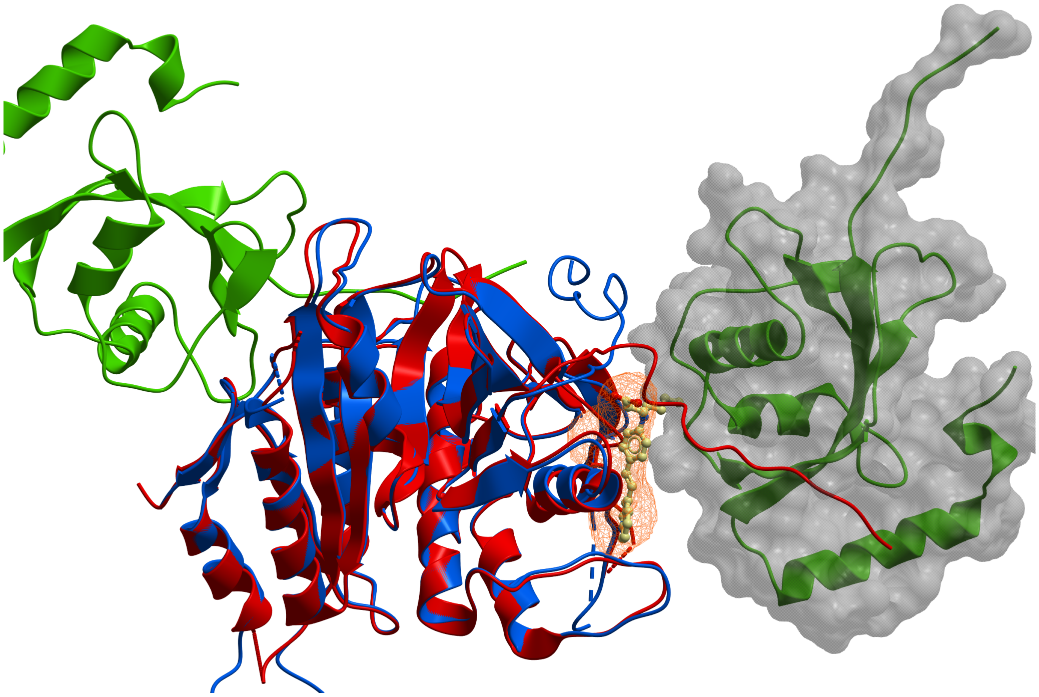

Figure S5A. ATG4B active conformation is in red color and inactive conformation is in blue. LC3 is in green. The non-substrate LC3 is depicted with a transparent skin. Compound **4-28** is shown in stick model and its pocket closed #2 in thin wire.

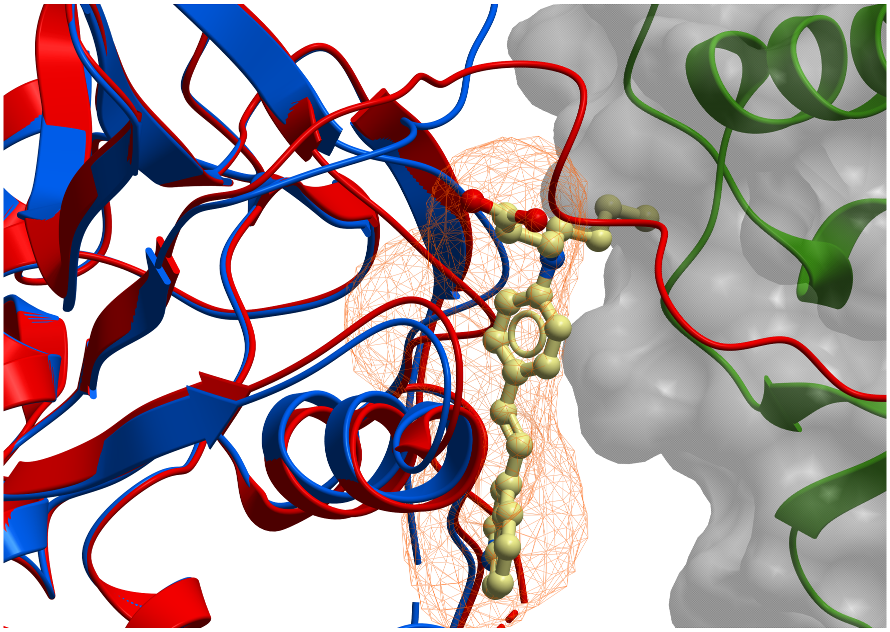

Figure S5B. Zoom in of compound **4-28** and its pocket.

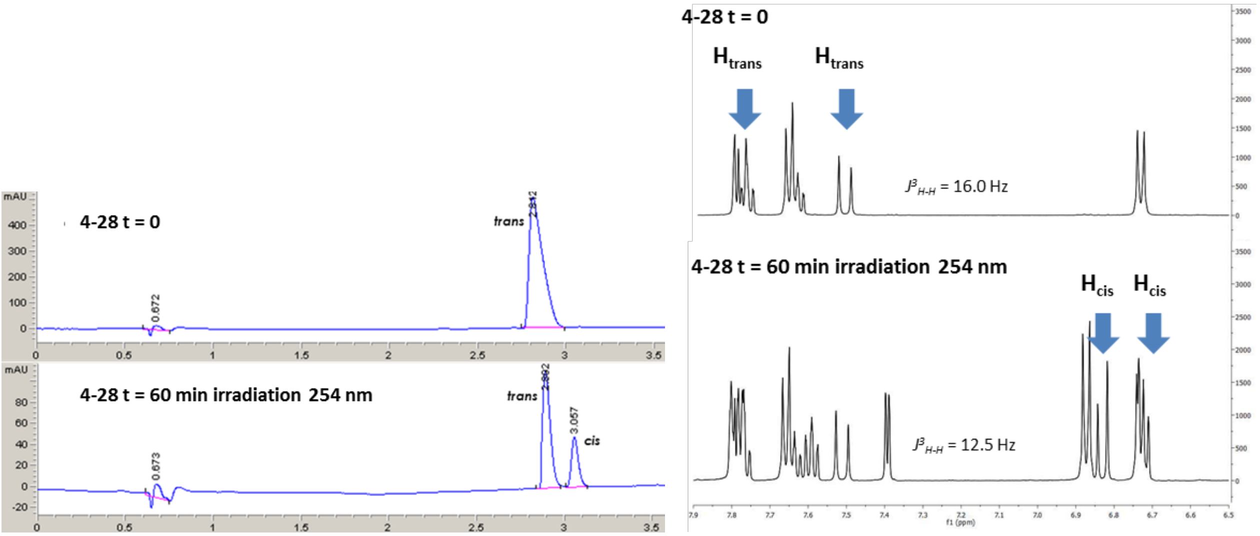

Figure S6. Instability of compound **4-28** under UV irradiation (254 nm) confirmed by HPLC (left) and NMR spectra (right)

4-28 experiments	1	2	3
4-28	+	+	+
1 irrad./15 min for 2h	-	+	-
Atg4B	-	+	-
irrad. 366 nm for 16h	-	-	+
trans (%)	100	94	43
cis (%)	0	4	56
other (%)	0	2	0

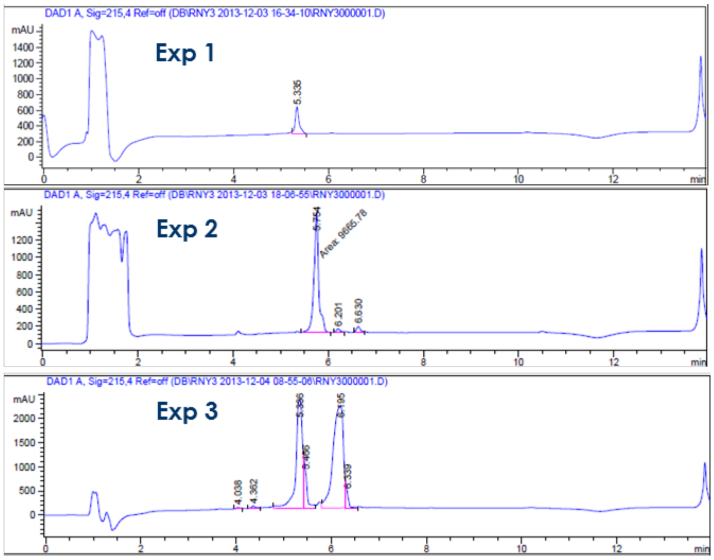

Figure S7. Integrity of compound **4-28** in the fluorimeter as analyzed by HPLC

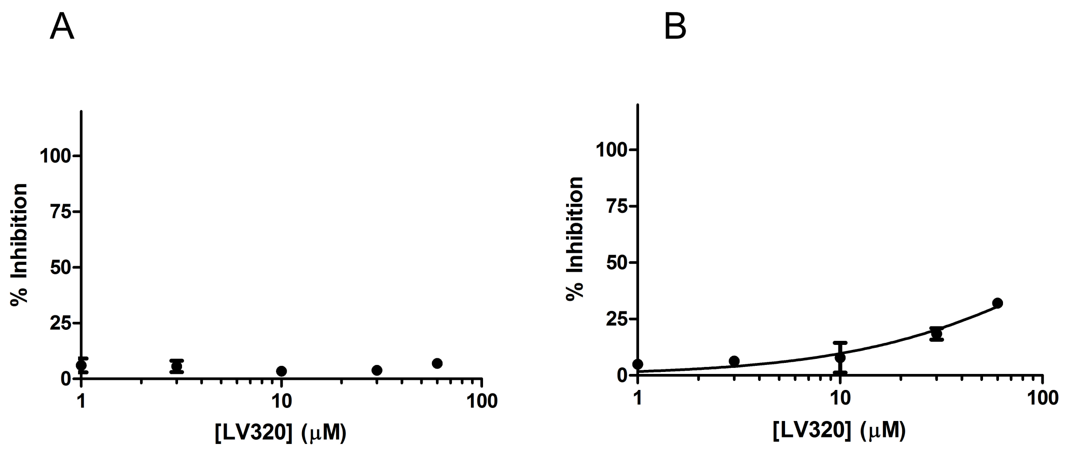

Figure S8: Inhibition curve for **LV-320** with caspase 3 (panel A) and cathepsin B (panel B)

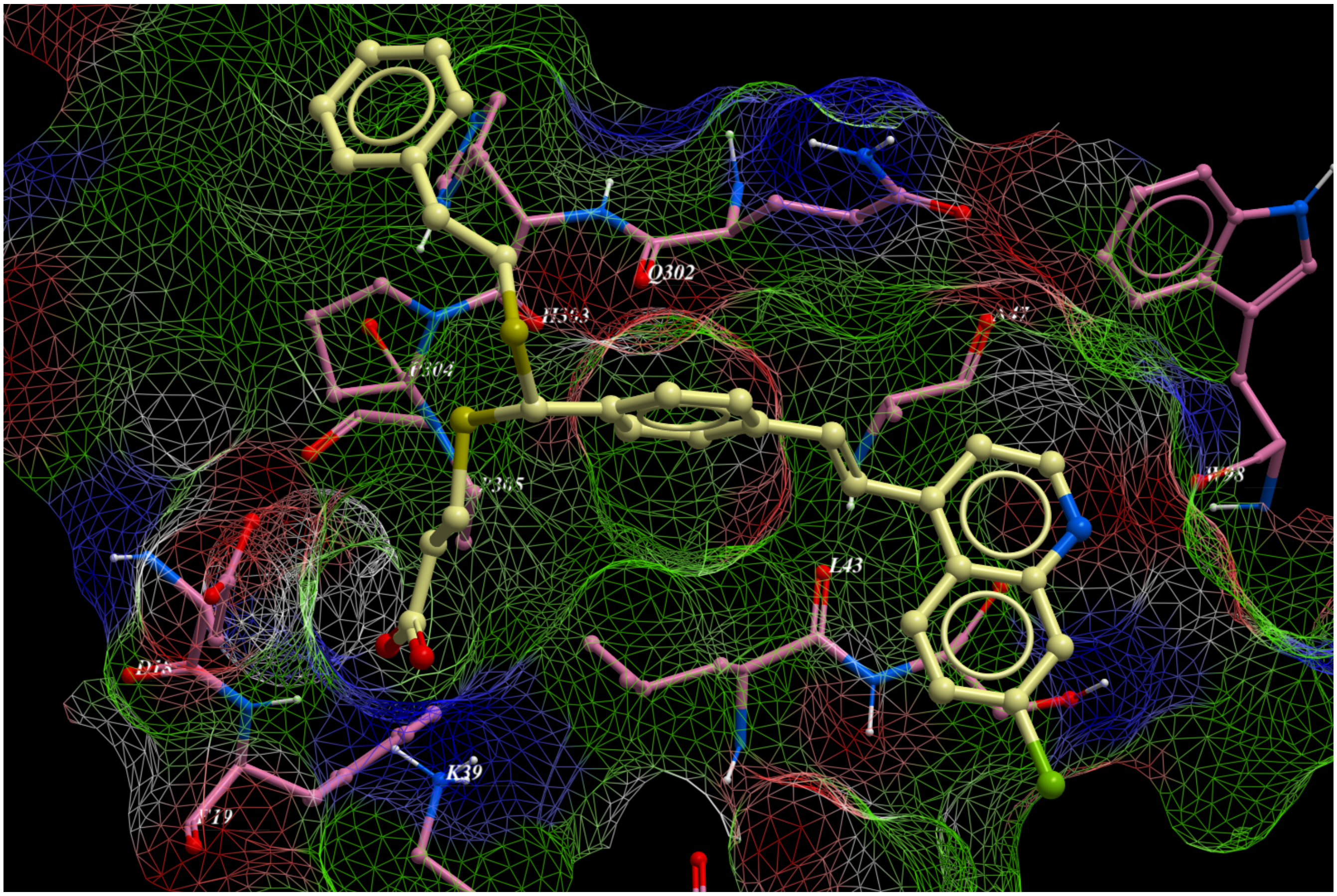

Figure S9. Representation of LV-320 binding to the putative binding site on ATG4B and the key interacting amino-acid residues

```
ATG4A        1   MESVLSKYEDQITIFTDYLEEYPDTDELVWILGKQHLLKTEKSKLLSDIS
ATG4B        5   ------TLTYDTLRFAEF-EDFPETSEPVWILGRKYSIFTEKDEILSDVA

ATG4A       51   ARLWFTYRRKFSPIGGTGPSSDAGWGCMLRCGQMMLAQALICRHLGRDWS
ATG4B       48   SRLWFTYRKNFPAIGGTGPTSDTGWGCMLRCGQMIFAQALVCRHLGRDWR

ATG4A      101   WEKQKEQPKEYQRILQCFLDRKDCCYSIHQMAQMGVGEGKSIGEWFGPNT
ATG4B       98   WTQRKRQPDSYFSVLNAFIDRKDSYYSIHQIAQMGVGEGKSIGQWYGPNT

ATG4A      151   VAQVLKKLALFDEWNSLAVYVSMDNTVVIEDIKKMCRVLPLSADTAGDRP
ATG4B      148   VAQVLKKLAVFDTWSSLAVHIAMDNTVVMEEIRRLCR-------------

ATG4A      201   PDSLTASNQSKGTSAYCSAWKPLLLIVPLRLGINQINPVYVDAFKECFKM
ATG4B      185   ------------TSVPCSPWRPLVLLIPLRLGLTDINEAYVETLKHCFMM

ATG4A      251   PQSLGALGGKPNNAYYFIGFLGDELIFLDPHTTQTFVDTEENGTVNDQTF
ATG4B      250   PQSLGVIGGKPNSAHYFIGYVGEELIYLDPHTTQPAVE---GCFIPDESF

ATG4A      301   HCLQSPQRMNILNLDPSVALGFFCKEEKDFDNWCSLVQKEILKENLRMFE
ATG4B      300   HCQHPPCRMSIAELDPSIAVGFFCKTEDDFNDWCQQVKKLSL---LPMFE

ATG4A      351   LVQKHPSHWPPFVPPAKPEVTTTGAEFIDSTEQLEEFDLEEDFEILSV
ATG4B      351   LVEQQPDVLNLSLDSSDVERL---------------------------
```

Figure S10. Sequence alignment of ATG4A and ATG4B with key amino-acid residues of the binding pocket indicated in green box

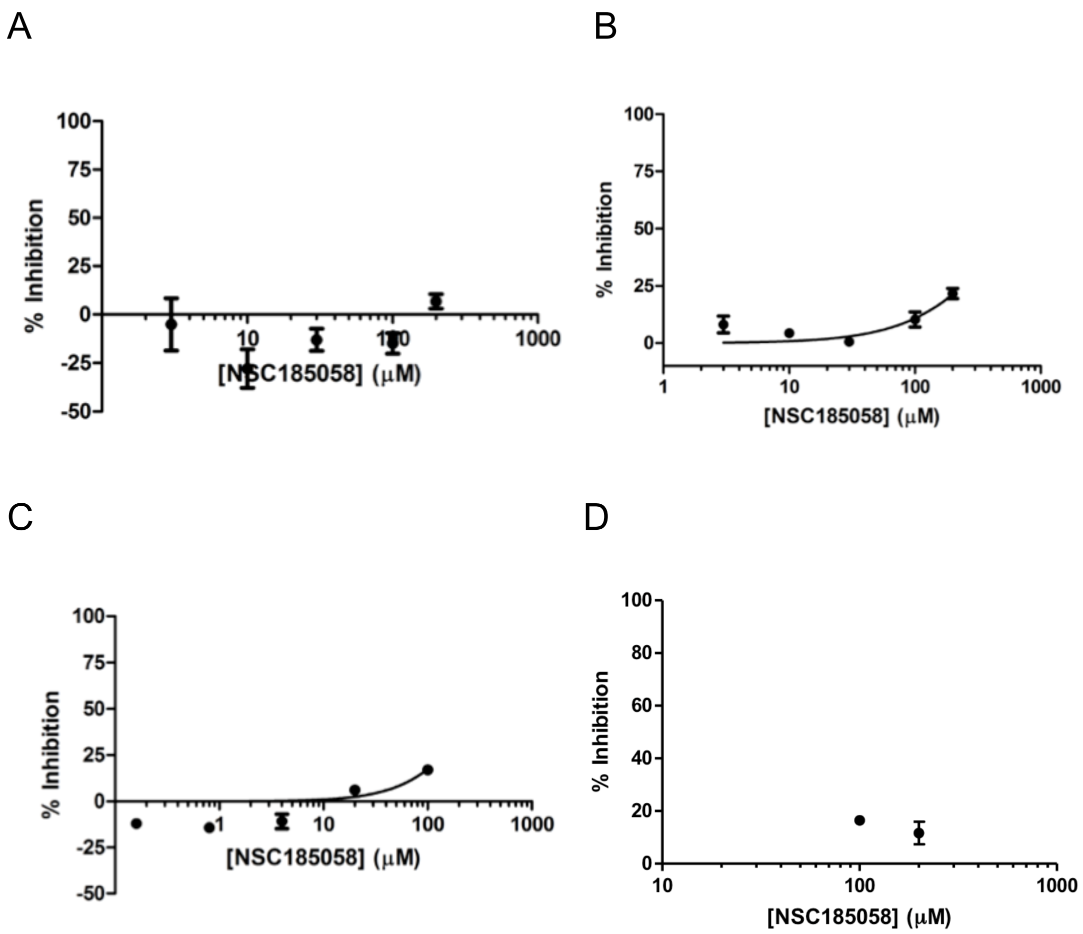

Figure S11. Inhibition of ATG4B activity observed for NSC185058 in A) the fluorescent peptide assay[6] with TCEP; B) the fluorescent peptide assay[6] using DTT as reducing agent; C) the FRET LC3 fluorescence assay[5] and D) the assay with FRET LC3 as substrate monitored by mass spectrometry[5]

.

Atg4B-1	Sense: 5' GCCCACUACUUCAUCGGCUACGUUG 3′<br>Antisense: 5'CAACGUAGCCGAUGAAGUAGUGGGC 3′
Atg4B-2	Sense: 5' UUCCAGAGUGUUCUCUCGACACUGC 3′<br>Antisense: 5'GCAGUGUCGAGAGAACACUCUGGAA 3′

Figure S12: Sequences of siRNA duplexes

SUPPLEMENTAL INFORMATION

Tables

Screening code	NCI Code	Structure	Titration curve (FRET LC3 assay)
**1-4**		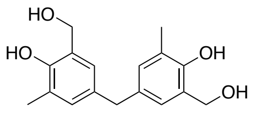	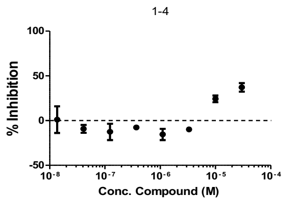
**2-22**		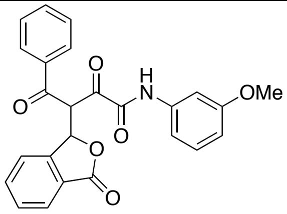	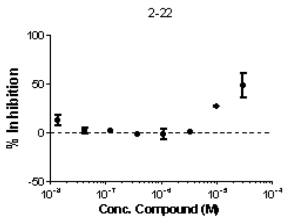
**3-22**		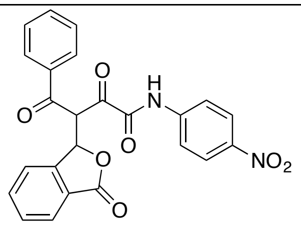	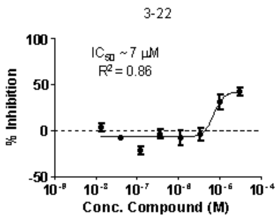
**4-6**		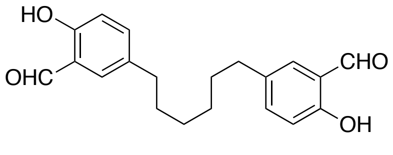	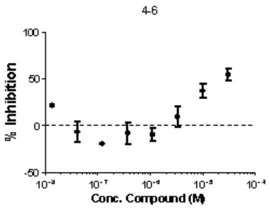
**4-28**		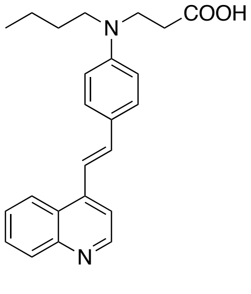	43% inhibition at 8 μM

SUPPLEMENTAL INFORMATION

Table S1 : Compound hits from NCI library in silico screen that showed significant cellular effects on GFP-LC3 puncta in SKBRF3-hrGFP-LC3B cells and inhibition of ATG4B activity listed according to screening codes, NCI codes and structures with titration curve (Assays were performed in triplicate according to the method in reference 5)

Compd	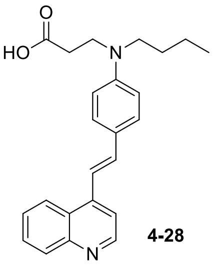 **4-28**	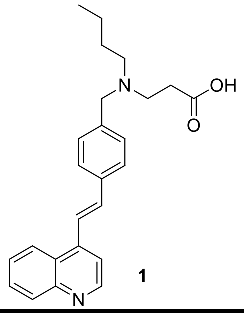 **1**	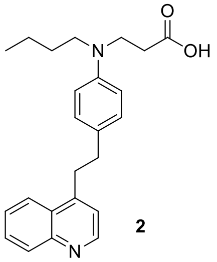 **2**	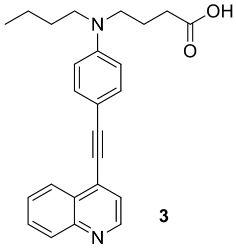 **3**
IC_{50} (μM)	79 ± 9	> 200	> 200	> 200
Compd	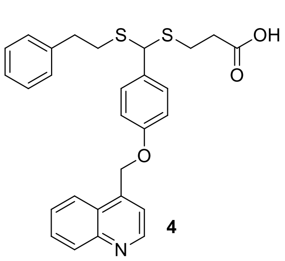 **4**	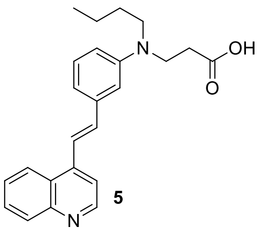 **5**	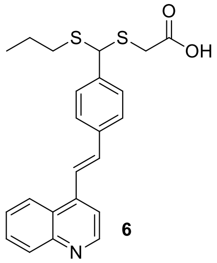 **6**	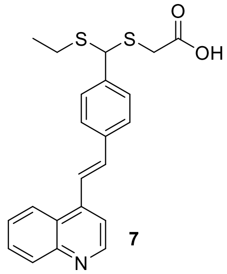 **7**
IC_{50} (μM)	> 200	110 ± 6	36 ± 7	> 200
Compd	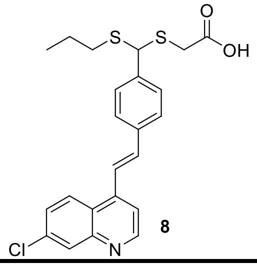 **8**	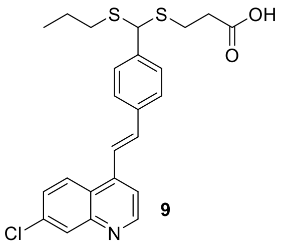 **9**	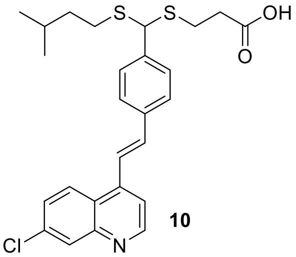 **10**	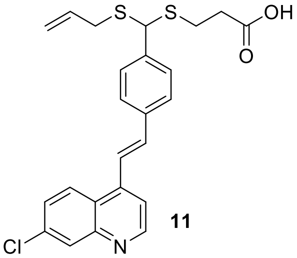 **11**
IC_{50} (μM)	47 ± 15	12 ± 3	29[a]	16[b]
Compd	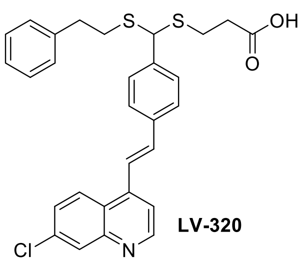 **LV-320**	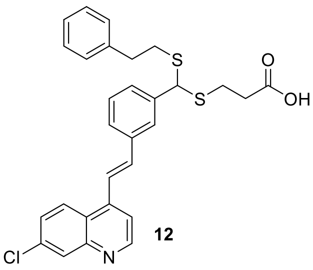 **12**	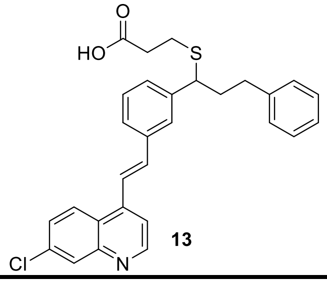 **13**	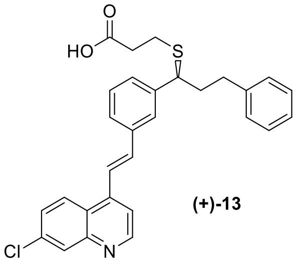 **(+)-13**
IC_{50} (μM)	24.5 (95% CL 20.7-29.1)	29[b]	13 ± 9	9.1 ± 1.0

Compd	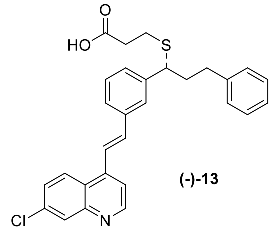 (-)-13	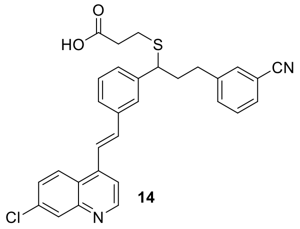 14	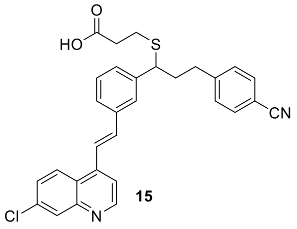 15	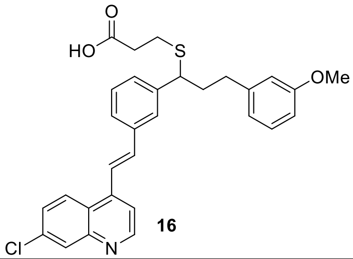 16
IC_{50} (µM)	9.4 ± 2	39 ± 9	11 ± 0.8	27[b]
Compd	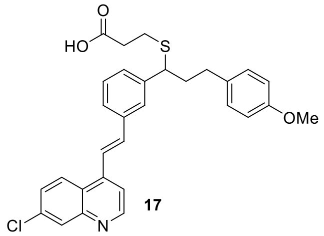 17	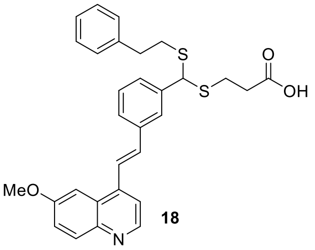 18	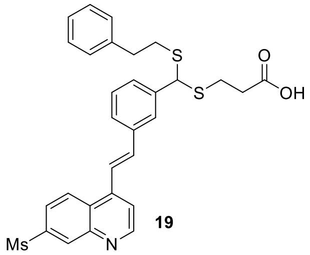 19	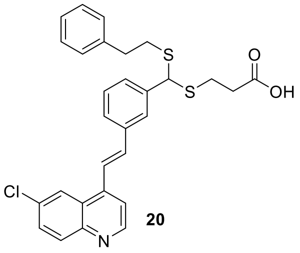 20
IC_{50} (µM)	30 ± 0.3	37 ± 21	15[a]	30[a]
Compd	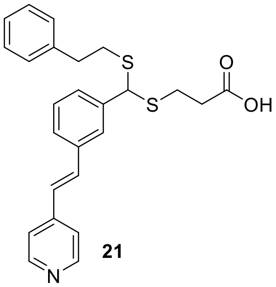 21	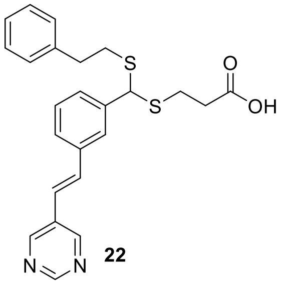 22	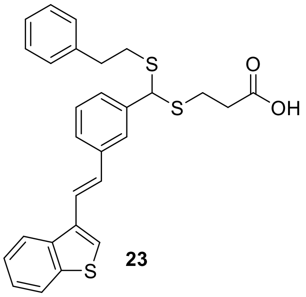 23	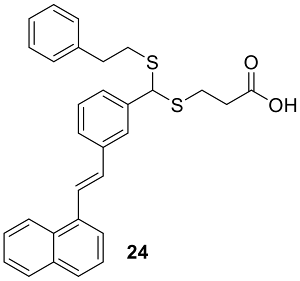 24
IC_{50} (µM)	> 100	> 100	22 ± 2	36[a]
Compd	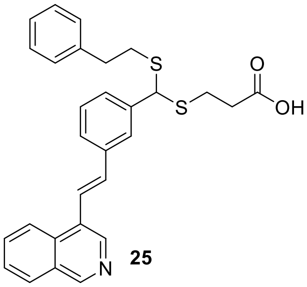 25	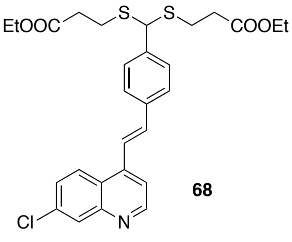 68	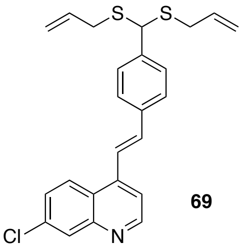 69	
IC_{50} (µM)	47 ± 12	> 100	> 100	

Table S2 ATG4B Inhibitory activity of **4-28** and synthesized compounds **1** to **69** and **LV-320**, related to Figure 4. Experiments are done in triplicates except where mentioned ([a] n=1; [b] n=2). IC_{50} are in µM and are represented as mean ± SD for triplicates or as mean for duplicates. Compound **13** is a racemate and **(+)-13** and **(-)-13** are depicted as ambiguous as to absolute stereochemistry as this was not determined.

IC_{50} are in μM and are represented as mean ± SD for triplicates or as mean for duplicates. Compound **13** is a racemate and **(+)-13** and **(-)-13** are depicted as ambiguous as to absolute stereochemistry as this was not determined.

SYNTHESIS, CHARACTERIZATION OF COMPOUNDS AND SPECTRA

Scheme S1. Homologation of 4-28

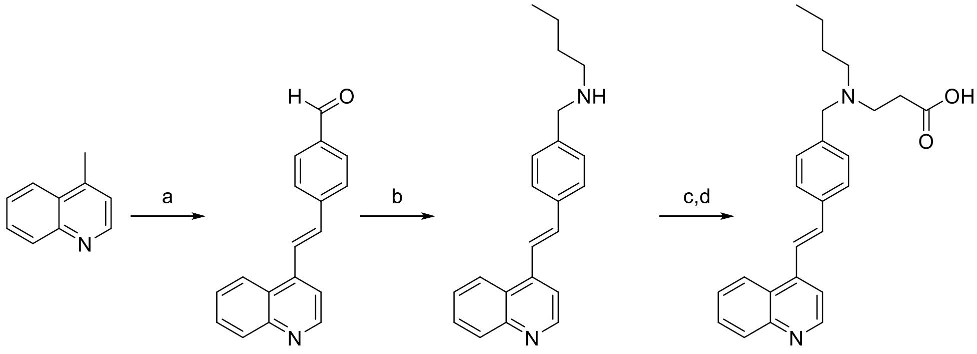

26 **27** **1**

Reagent and conditions: (a) terephthalaldehyde, $ZnCl_2$, MW, 150 °C, 10 min, 51%; (b) *n*-butylamine, $NaBH(OAc)_3$, DMF, MW, 30 min, 47%; (c) ethyl 3-bromopropionate, K_2CO_3, DMF, MW, 90 °C, 10 h ; (d) 2N NaOH, dioxane, H_2O, 50 °C, 50%.

Scheme S2. Replacement of the double bond by a single bond

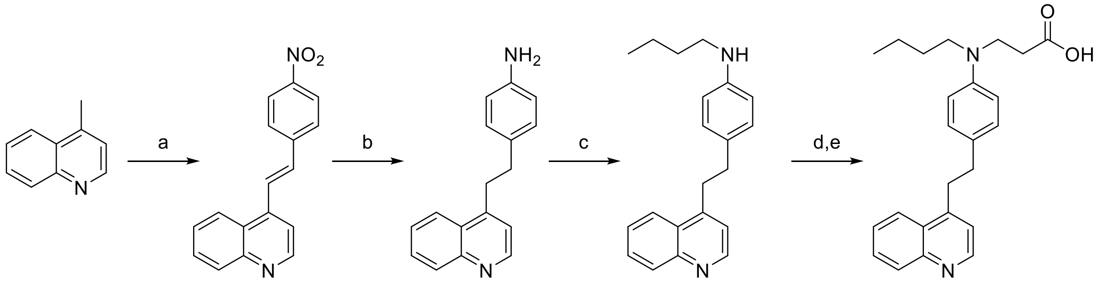

28 **29** **30** **2**

Reagent and conditions: (a) 4-nitrobenzaldehyde, $ZnCl_2$, MW, 150 °C, 10 min, quantitative yield; (b) 10% Pd/C, H_2, DMF, rt, 24 h, 57%; (c) butyraldehyde, $NaBH(OAc)_3$, THF, rt, 2 h, 30%; (d) ethyl 3-bromopropionate, DIEA, MW, 100 °C, 2 h; (e) 2N NaOH, dioxane, H_2O, 50 °C, 10% for two steps.

Scheme S3. Replacement of the double bond by a triple bond

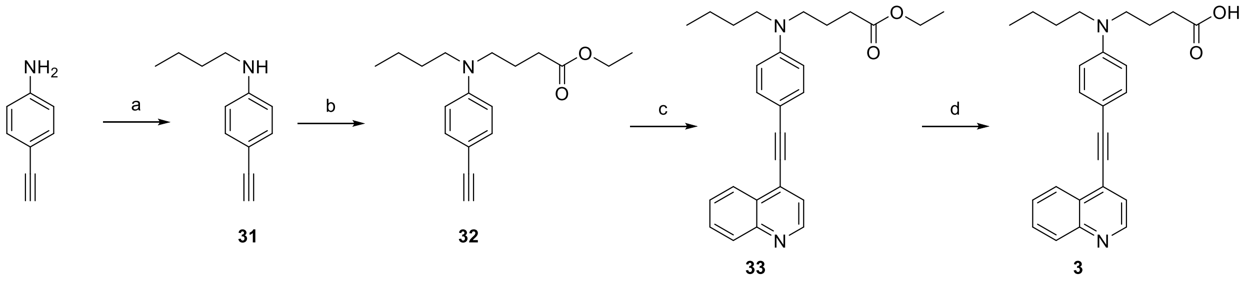

Reagent and conditions: (a) butyraldehyde, $NaBH(OAc)_3$, THF, rt, 4 h, 41%; (b) ethyl 4-bromobutyrate, K_2CO_3, DMF, MW, 90 °C, 8 h, 28%; (c) 4-bromoquinoline, Et_3N, CuI, $PdCl_2(PPh_3)_2$, DMF, MW, 60 °C, 90 min, 80%; (d) 10N NaOH, MeOH, H_2O, rt, 6 h, 59%.

Scheme S4: Synthesis of the ether analog 4

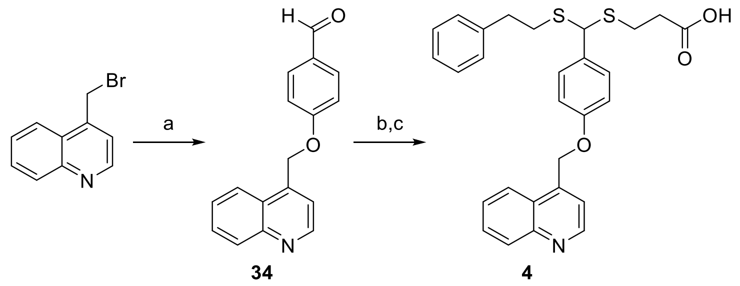

Reagent and conditions: (a) 4-hydroxybenzaldehyde, K_2CO_3, KI, THF, rt, 48 h, 40%; (b) Ethyl 3-mercaptopropionate, 2-phenylethanethiol, PTSA, toluene, reflux, 5 h; (c) 10N NaOH, MeOH, H_2O, -10 °C to rt, 53% over two steps.

Scheme S5. Synthesis of compound 5

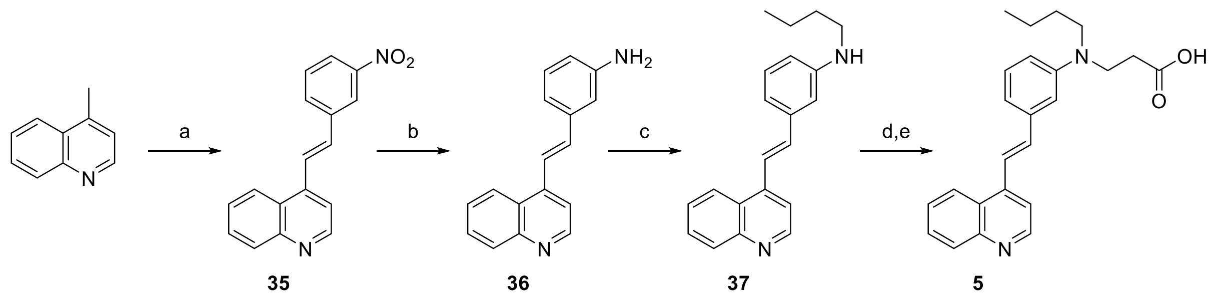

Reagent and conditions: (a) 3-nitrobenzaldehyde, $ZnCl_2$, NMP, MW, 150 °C, 34%; (b) $SnCl_2$, EtOH, reflux, 45%; (c) butyraldehyde, $NaBH(OAc)_3$, AcOH, CH_2Cl_2, rt, 38%; (d) ethyl 3-bromopropionate, K_2CO_3, KI, DMF, 70 ^{o}C (e) 4N NaOH, dioxane, H_2O, rt, 42%.

Scheme S6. Synthesis of the dithioacetal analogues

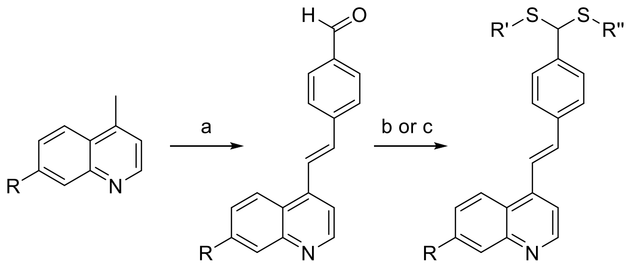

26, R = H
38, R = Cl

6-7, R = H
8-11, LV-320, R = Cl

Reagent and conditions: (a) terephthalaldehyde, $ZnCl_2$, MW, 150 °C, 20 min, yields ranging from 51% to 70%; (b) thiol 1, thiol 2, PTSA, toluene, reflux, 2 h, yields ranging from 6 to 42%; (c) (I) thiol 1, thiol 2, PTSA, toluene, reflux, 2 h (II) 2N NaOH, MeOH, rt, 2 h, 7 %.

Scheme S7. Synthesis of quinoline analogs

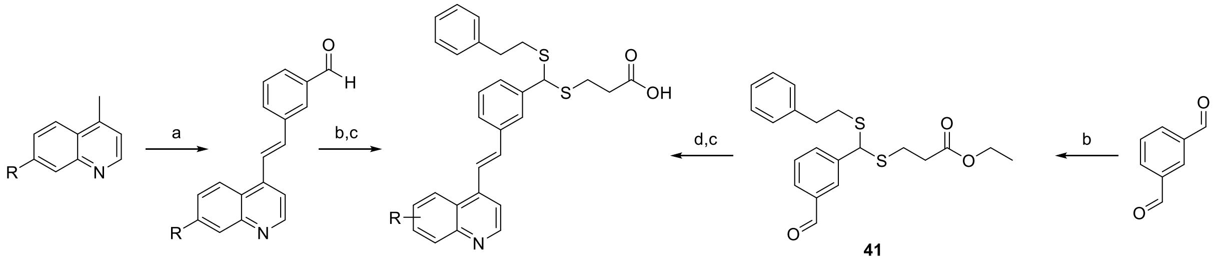

41

R = Cl, **39**
R = Ms, **40**

R = 7-Cl, **12**
R = 6-OMe, **18**
R = 7-Ms, **19**
R = 6-Cl, **20**

Reagent and conditions: (a) isophthalaldehyde, $ZnCl_2$, MW, 150 °C, 20 min, yields ranging from 38% to 81%; (b) ethyl 3-mercaptopropionate, 2-phenylethanethiol, PTSA, toluene, rt or reflux, 2.5 h - 3 h; (c) 2N NaOH, MeOH, H_2O, $^{-}15\ ^{o}C$ - rt, 2 h, yields ranging from 7 to 36%; (d) R-substituted quinoline, Ac_2O or $ZnCl_2$, MW, 150 °C - 190 °C, 20 min - 6 h, yields ranging from 4% to 10%.

Scheme S8. Synthesis of the thioether analogs

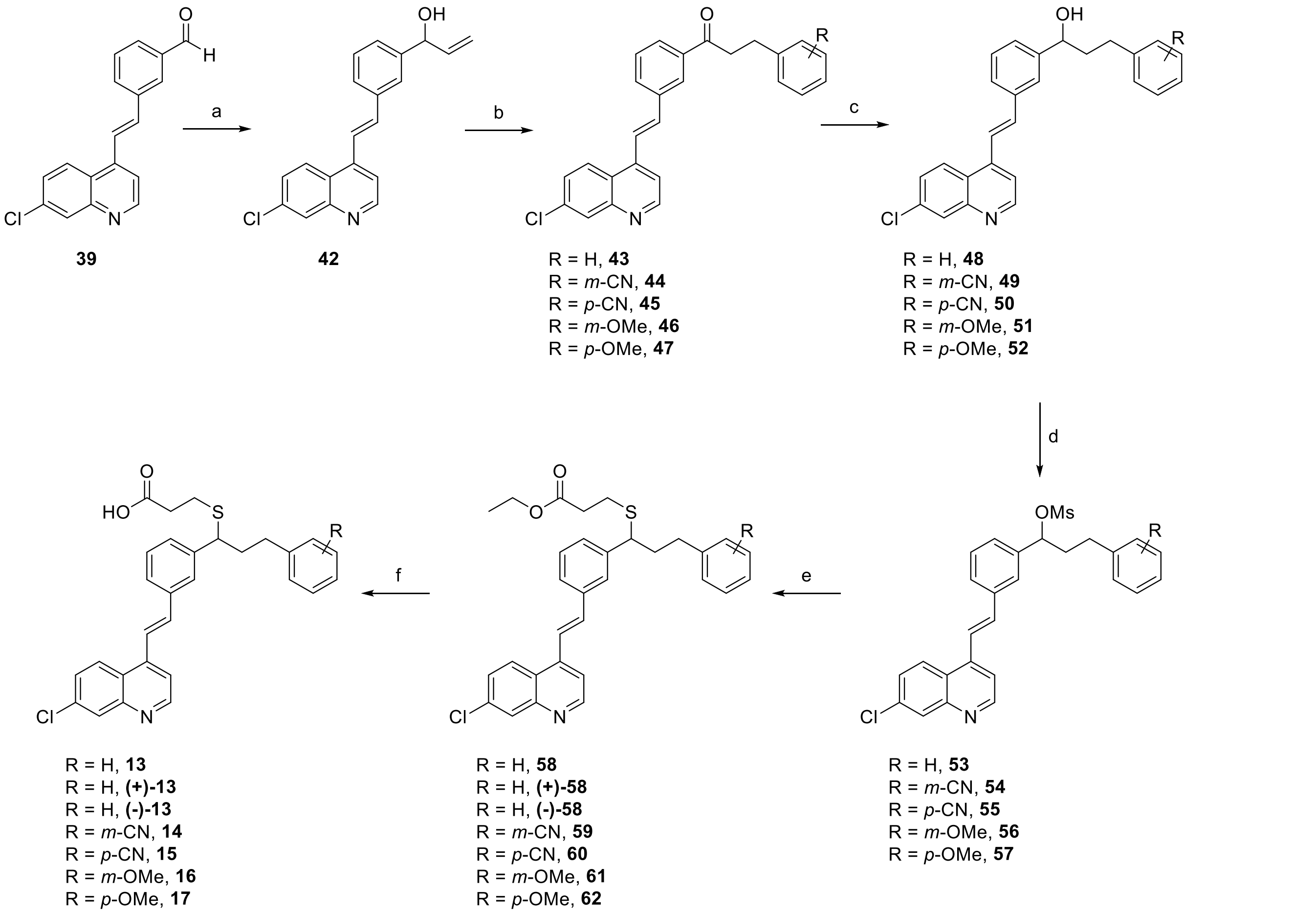

Reagent and conditions: (a) vinyl magnesium bromide, toluene, 5 °C, 2.5 h, 52%; (b) aryl iodide, Et_3N, CH_3CN, reflux, 16 h, yields ranging from 47% to 85%; (c) $NaBH_4$, THF/MeOH (1:1), rt, 16 h, yields ranging from 60% to Quant.; (d) MsCl, Et_3N, toluene, 0 °C, 16 h; (e) ethyl 3-mercaptopropionate, Cs_2CO_3, CH_3CN or DMF, rt, 3 h - 16 h, yields ranging from 46% to 55%; (f) $LiOH.H_2O$, CH_3CN, H_2O, rt, 16 h, yields ranging from 23% to 82%.

Scheme S9. Synthesis of analogs modulating the aromatic ring

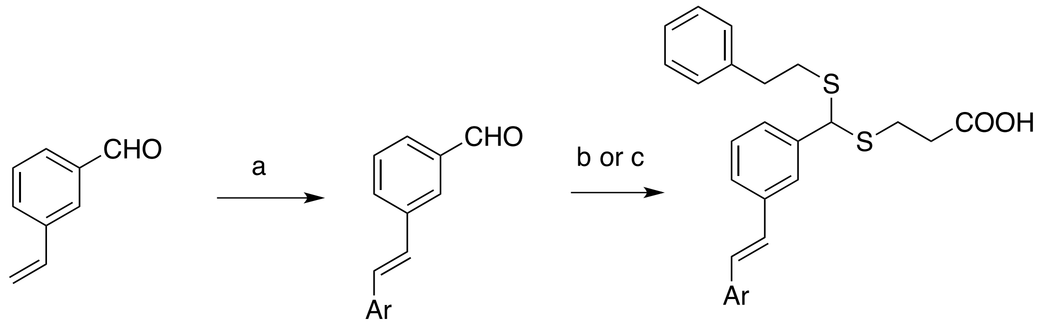

63: Ar = 4-pyridyl
64; Ar = 5-[1,3]pyrimidyl
65; Ar = 3-benzothiaphinyl
66; Ar = 1-naphthyl
67; Ar = 4-isoquinolinyl

21: Ar = 4-pyridyl
22; Ar = 5-[1,3]pyrimidyl
23; Ar = 3-benzothiaphinyl
24; Ar = 1-naphthyl
25; Ar = 4-isoquinolinyl

Reagents and conditions: (a) aryl bromide, NaOAc or Et_3N, $Pd(OAc)_2$, $P(O\text{-}tol)_3$, CH_3CN or DMA, reflux, 16 h, yields ranging from 10% - 74%; (b) 3-mercaptopropionic acid, 2-phenylethanethiol, TsOH.H_2O, CH_2Cl_2, reflux, 1 h, yields ranging from 17% - 23%.
(c) (I) 3-mercaptopropionic acid, 2-phenylethanethiol, TFA, DCE, 0 °C to rt, 16 h; (II) 2N NaOH, MeOH, H_2O, $^{-}15\,^{o}C$ - rt, 2 h, yields ranging from 5 to 42%.

General methods and materials

All reagents, solvents and starting materials were purchased from commercial suppliers and used without further purification. ^1H NMR spectra were recorded on a Bruker Avance II™ 600 MHz, Bruker Avance III™ 500 MHz or Bruker Avance III™ 400 MHz spectrometer with acetone-d_6, $CDCl_3$ or DMSO-d_6 as the solvent. ^{13}C NMR spectra are recorded at 101 or 151 MHz. All coupling constants are measured in hertz (Hz) and the chemical shifts (δ) are quoted in parts per million (ppm). Processing of the spectra was performed with MestRec™ software. The high-resolution mass spectra were recorded either in positive or negative ion-mode with an ESI or multimode ESI/APCI ion source on an Agilent™ 6210 Time-of-Flight LC/MS mass spectrometer. Purity for all final compounds was confirmed by analytical reverse-phase HPLC utilizing a Halo® C18 reverse-phase analytical column (4.6 × 50 mm, 5 mM), using UV detection (215 nM) and a gradient starting from 86 % H_2O / 14 % CH_3CN / 5 mM NH_4OAc and reaching 14 % H_2O / 86 % CH_3CN / 5 mM NH_4OAc within 6 min. Optical rotation was

measured on a Perkin-Elmer Polarimeter 341 at 589 nm. Analytical thin-layer chromatography (TLC) was performed on aluminum plates pre-coated with silica gel 60F-254 as the absorbent. The developed plates were air-dried, exposed to UV light and/or dipped in KMnO4 solution and heated. All automated flash chromatography were performed on Biotage Isolera Flash Purification Systems using commercial silica gel cartridges.

General procedures

Procedure ***A***: Heck coupling with 3-vinylbenzaldehyde and halogen-substituted aryl

Aldehyde (1.0 mmol) in CH_3CN (0.55 mL) was treated with halogen-aryl (1.1 mmol), triethylamine (1.5 – 2.5 mmol), and palladium acetate (0.02 mmol). The mixture was heated at reflux under nitrogen for 14 h. The reaction mixture was diluted with EtOAc and H_2O. The separated organic layer was washed with water and brine, dried over Na_2SO_4 and concentrated under reduced pressure. The crude product was purified by flash chromatography on silica gel column (hexanes:EtOAc; 10:0 to 25:75).

Procedure ***B***: Asymmetric dithioacetal formation with TFA

Syntheses generally followed procedures previously reported by Gauthier [3] and Zamboni. [4] The aldehyde (1.0 mmol) was solubilized in 1,2-DCE (10.0 mL) and TFA (5.0 mL) at 0 °C. The 3-mercaptopropionic acid (1.1 mmol) and 2-phenylethanethiol (1.1 mmol) were added and the reaction mixture was stirred at room temperature for 16 h. The crude reaction mixture was purified by reverse phase chromatography (water:MeOH; 1:0 to 0:1) to afford the dithioacetal.

Procedure ***C***: Asymmetric dithioacetal formation with $TsOH.H_2O$

Syntheses followed procedures previously reported by Gauthier [3] and Zamboni. [4] The aldehyde (1.0 mmol) and $TsOH.H_2O$ (1.1 mmol) were solubilized in CH_2Cl_2 (0.5 mL). Thiol 1(1.1 mmol) and Thiol 2 (1.1 mmol) were added and the reaction mixture was stirred at 50 °C for 1 h. The reaction mixture was diluted with 12.5 mL of a MeOH:water:HCl:DMF (4:4:1:4) solution. The crude reaction mixture was purified by reverse phase chromatography (H_2O:MeOH; 9:1 to 0:1) to afford the dithioacetal.

Procedure ***D***: Reduction of ketone with $NaBH_4$

A mixture of ketone (1.0 mmol), tetrahydrofuran (2.3 mL) and ethanol (2.3 mL) was added to sodium borohydride (0.5 mmol) at 5 °C. The colorless reaction mixture was stirred at room temperature for 1 h, and then acidified with a 10% aqueous solution of citric acid. The mixture was extracted with CH_2Cl_2, washed with brine, dried over Na_2SO_4 and concentrated under reduced pressure to afford the alcohol with a purification step if needed.

Procedure ***E***: Mesylation of alcohol

Alcohol (1.0 mmol) in toluene (6.4 mL) was cooled at -10 °C. Triethylamine (1.8 mmol) was added followed by the dropwise addition of methanesulfonyl chloride (2.5 mmol). The reaction mixture was stirred at 0 °C for 16 h. The reaction mixture was diluted in CH_2Cl_2 and 10% citric acid. The aqueous layer was washed with CH_2Cl_2. The collected organic layer was washed with 10% citric acid and saturated aqueous Na_2CO_3 solution, dried over Na_2SO_4 and concentrated to afford the mesylated alcohol, which was used without further purification in the next step.

Procedure ***F***: Thiolation of mesylated alcohol

The mesylate (1.0 mmol) in CH_3CN or DMF (10 mL) was degassed by purging three times with vacuum and nitrogen. Ethyl 3-mercaptopropionate (1.5 mmol) was added at room temperature followed by addition of Cs_2CO_3 (1.8 mmol). The reaction mixture was stirred at room temperature for 3 h – 16 h. The solids were filtered and washed with CH_3CN. The filtrate was evaporated to dryness to give a yellow oil. The crude product was purified by flash chromatography on silica gel (toluene/EtOAc 10:0 to 75:25 (v/v) in 30 min) to afford the thioether.

Procedure ***G***: Saponification of ethyl 3-(thioether)propanoate

To a solution (CH_3CN:H_2O; 3:2, 26 mL) of ester (1.0 mmol) at 0 °C was added lithium hydroxide monohydrate (3.0 mmol). The mixture was stirred for 16 h. The aqueous layer was acidified with acetic acid until pH 3 and extracted twice with ethyl acetate. The combined organic layers were dried over Na_2SO_4 and concentrated under reduced pressure. The crude product was purified by flash chromatography on silica gel column (hexanes to toluene/EtOAc:CH_3COOH; 1:0:0:0 to O:1:1:0.01) to afford the acid.

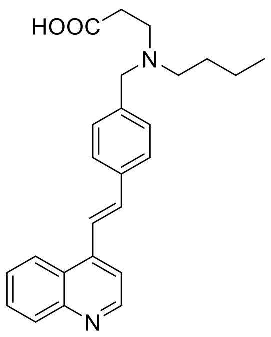

(E)-3-(butyl(4-(2-(quinolin-4-yl)vinyl)benzyl)amino)propanoic acid (**1**)

Compound **26** (259 mg, 1 mmol), *n*-butylamine (143 µL, 1.5 mmol) and $NaHB(OAc)_3$ (422 mg, 2 mmol) were combined under inert atmosphere. DMF (2 mL) was added and the mixture was stirred under microwave irradiation for 30 min. The reaction was quenched with 2M NaOH and extracted with EtOAc. The organic phase was washed with 2M NaOH, dried over Na_2SO_4, filtered and concentrated under reduced pressure. The residue was purified by flash chromatography on a silica gel column (CH_2Cl_2:MeOH:TEA; 99:1:1 to 90:10:1) to yield *(E)-N*-(4-(2-(quinolin-4-yl)vinyl)benzyl)butan-1-amine (**27**) as a brown oil (150 mg) which was used directly in the following reaction

To a solution of **27** (65 mg, 0.205 mmol) in (DMF, 0.5 mL) was added ethyl 3-bromopropionate (131 mg, 1.025 mmol) and K_2CO_3 (140 mg, 1.02 mmol). The mixture was stirred under microwave irradiation at 90 ^oC for 10 h. The mixture was filtered and the solvent removed under reduced pressure. The crude ester was purified by preparative reverse phase chromatography (CH_3CN:H_2O; 20:80 to 100:0). The ester was dissolved in dioxane (0.5 mL) and 2N NaOH solution (20 µL) was added. The reaction was stirred at 50 ^oC for 14 h. The solvent was removed under reduced pressure. The residue was dissolved in an acetonitrile/H_2O/formic acid solution (1:1:0.01 (v/v), 5 mL) and purified by preparative reverse phase chromatography (CH_3CN:H_2O; 20:80 to 100:0) to afford **1** as a yellow solid (40 mg, 50%). ^1H NMR (DMSO-d_6) δ (ppm): 8.89 (d, 1H, *J* = 3.8 Hz), 8.53 (d, 1H, *J* = 7.9 Hz), 8.08 (d, 1H, *J* = 16.2 Hz), 8.04 (d, 1H, *J* = 6.9 Hz), 7.85 (d, 1H, *J* = 4.6 Hz), 7.79 (ddd, 1H, *J* = 6.7, 5.0, 1.3 Hz), 7.79 (d, 2H, *J* = 4.9 Hz), 7.66 (ddd, 1H, *J* = 8.2, 6.8, 1.1 Hz), 7.59 (d, 1H, *J* = 16.1 Hz), 7.38 (d, 2H, *J* = 7.5 Hz), 3.60 (s, 2H), 2.71 (s, 2H), 2.39 (d, 4H, *J* = 8.2 Hz), 1.42 (s, 2H), 1.25 (dd, 2H, *J* = 14.4, 7.2 Hz), 0.83 (t, 3H, *J* = 7.3 Hz). ^{13}C NMR (DMSO-d_6) δ (ppm): 174.3, 150.8,

148.9, 142.8, 140.7, 135.7, 135.5, 130.1, 130.0, 129.6, 127.9, 127.1, 126.4, 124.7, 122.4, 117.1, 57.8, 53.0, 49.5, 32.4, 29.0, 20.5, 14.4.

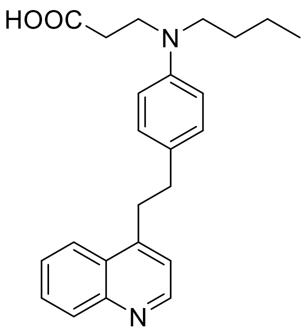

3-(butyl(4-(2-(quinolin-4-yl)ethyl)phenyl)amino)propanoic acid (**2**)

To a solution of **30** (40 mg, 0.131 mmol) in DIEA (90 µL, 0.353 mmol) was added ethyl 3-bromopropionate3-ethoxy-3-oxopropyl benzoate (168 µL, 1.31 mmol). The mixture was stirred under microwave irradiation at 100 °C for 2 h. The mixture was diluted with EtOAc and washed with water, saturated $NaHCO_3$ and brine, dried over Na_2SO_4, filtered and the filtrate was concentrated under reduced pressure. The crude ester was purified by flash chromatography on a silica gel column (MeOH:CH_2Cl_2; 1:99 to 10:90). The ester was dissolved in dioxane (7.5 mL) and 2N NaOH solution (300 µL) was added. The reaction was stirred at 50 °C for 14 h. The solvent was removed under reduced pressure. The residue was dissolved in an acetonitrile/H_2O/formic acid solution (1:1:0.01 (v/v), 5 mL) and purified purified by preparative reverse phase chromatography (CH_3CN:H_2O:formic acid; 30:70:0.1 to 50:50:0.1) to afford **2** as a yellow oil (21%). ^{1}H NMR (DMSO-d_6) δ (ppm): 8.79 (d, 1H, J = 4.4 Hz), 8.21 (d, 1H, J = 8.4 Hz), 8.03 (d, 1H, J = 8.4 Hz), 7.76 (t, 1H, J = 7.6 Hz), 7.64 (t, 1H, J = 7.6 Hz), 7.39 (d, 1H, J = 4.4 Hz), 7.09 (d, 2H, J = 8.1 Hz), 6.60 (d, 2H, J = 8.1 Hz), 3.51 (t, 2H, J = 7.1 Hz), 3.36 – 3.30 (m, 2H), 3.24 (t, 2H, J = 7.6 Hz), 2.88 (dd, 2H, J = 9.6, 6.5 Hz), 2.46 – 2.39 (m, 2H), 1.47 (p, 2H, J = 7.5 Hz), 1.31 (h, 2H, J = 7.4 Hz), 0.91 (t, 3H, J = 7.3 Hz).

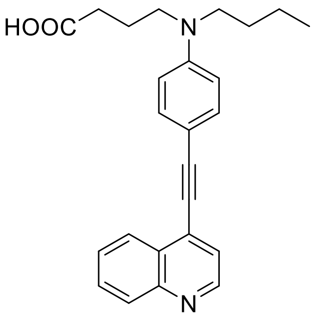

4-(butyl(4-(quinolin-4-ylethynyl)phenyl)amino)butanoic acid (**3**)

To a solution of **33** (17 mg, 0.041 mmol) in MeOH (0.4 mL) was added a 10N NaOH solution (50 μL). The reaction was stirred at rt for 6 h. The mixture was diluted with H_2O (10 mL) and purified by reverse phase chromatography (CH_3CN:H_2O:HOAc; 30:70:0.1 to 50:50:0.1) to afford **3** as yellow crystals (10 mg, 59%). ^1H NMR (DMSO-d_6) δ (ppm): 8.80 (d, 1H, *J* = 4.5 Hz), 8.32 (d, 1H, *J* = 8.2 Hz), 8.02 (d, 1H, *J* = 8.4 Hz), 7.82 (t, 1H, *J* = 7.6 Hz), 7.72 (t, 1H, *J* = 7.5 Hz), 7.59 (d, 1H, *J* = 4.5 Hz), 7.48 (d, 2H, *J* = 8.7 Hz), 6.73 (d, 2H, *J* = 8.8 Hz), 3.33 – 3.22 (m, 4H), 1.96 (t, 2H, *J* = 7.0 Hz), 1.67 (dd, 2H, *J* = 15.0, 7.5 Hz), 1.53 – 1.43 (m, 2H), 1.34 – 1.23 (m, 2H), 0.88 (t, 3H, *J* = 7.3 Hz).

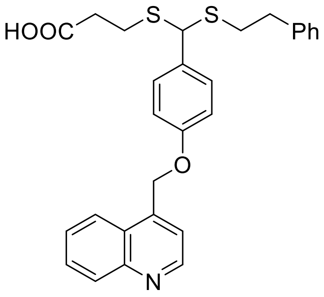

3-(((phenethylthio)(4-(quinolin-4-ylmethoxy)phenyl)methyl)thio)propanoic acid (**4**)

To a solution of **34** (30 mg, 0.114 mmol) and TsOH.H_2O (26 mg, 0.139 mmol) in toluene (1 mL) was added ethyl 3-mercaptopropionate (43 μL, 0.342 mmol) and 2-phenylethanethiol (45 μL, 0.342 mmol). The mixture was stirred at reflux for 5 h. The mixtue was diluted with EtOAc, washed with saturated $NaHCO_3$ and brine, dried over Na_2SO_4, filtered and the filtrate concentrated under reduced pressure. The crude dithioacetal was then purified by flash chromatography on a silica gel column (EtOAc:hexanes; 20:80 to 50:50). The dithioacetal was dissolved in MeOH (200 μL), 10N NaOH (50 μL) was added and the reaction was stirred at rt for 14 h. The mixture was diluted with H_2O and washed with Et_2O. The aqueous phase was

acidified to pH 5 and extracted with EtOAc. The organic phase was concentrated under reduced pressure and the residue was purified by reverse phase chromatography (H_2O:CH_3CN:HOAc; 100:0:0.1 to 50:50:0.1) to afford **4** as white crystals (17 mg, 53%). ^1H NMR (DMSO-d_6) δ 8.91 (d, 1H, *J* = 4.3 Hz), 8.17 (d, 1H, *J* = 8.3 Hz), 8.08 (d, 1H, *J* = 8.4 Hz), 7.80 (t, 1H, *J* = 7.6 Hz), 7.69 – 7.66 (m, 1H), 7.65 (d, 1H, *J* = 4.4 Hz), 7.38 (d, 1H, *J* = 8.5 Hz), 7.27 (t, 1H, *J* = 7.5 Hz), 7.26 (d, 1H, *J* = 7.2 Hz), 7.19 (t, 1H, *J* = 6.6 Hz), 7.18 (d, 2H, *J* = 7.6 Hz), 7.10 (d, 2H, *J* = 8.5 Hz), 5.66 (s, 2H), 5.12 (s, 1H), 2.85 – 2.74 (m, 3H), 2.72 – 2.63 (m, 2H), 2.62 – 2.55 (m, 1H), 2.08 (t, 2H, *J* = 7.8 Hz). ^{13}C NMR (DMSO-d_6) δ (ppm): 158.3, 151.4, 148.5, 143.2, 141.4, 134.7, 130.5, 130.4, 129.8, 129.4, 129.2, 127.7, 127.0, 126.5, 124.9, 120.4, 115.6, 67.1, 52.4, 36.2, 34.2, 30.4.

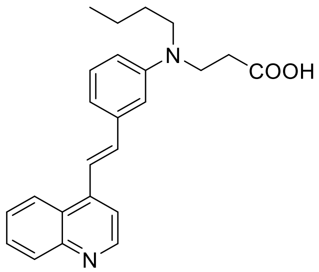

(E)-3-(butyl(3-(2-(quinolin-4-yl)vinyl)phenyl)amino)propanoic acid (**5**)

Compound **37** (33 mg, 0.109 mmol), K_2CO_3 (155 mg, 1.12 mmol) and KI (36 mg, 0.217 mmol) were suspended in DMF (0.2 mL) under an inert atmosphere. Ethyl 3-bromopropionate (155 µL, 01.12 mmol) was added and the reaction was stirred at 70 ^oC for 48 h. The mixture was diluted with MeOH (2 mL), 4M NaOH (0.2 mL) was added and the reaction was stirred at rt for 48 h. The solvent was removed under reduced pressure and the residue was purified by reverse phase chromatography (H_2O:MeOH:HOAc;95:5:0.1 to 5:95; :0.1) to afford **5** as a yellow powder (18 mg, 42%). ^1H NMR (D_2O) δ (ppm): 8.03 (d, 1H, *J* = 3.7 Hz), 7.52 (d, 1H, *J* = 8.2 Hz), 7.19 (dd, 2H, *J* = 15.5, 7.9 Hz), 6.82 (t, 1H, *J* = 7.0 Hz), 6.74 (d, 2H, *J* = 14.2 Hz), 6.64 (d, 1H, *J* = 3.8 Hz), 6.49 (d, 1H, *J* = 15.9 Hz), 6.34 (d, 1H, *J* = 7.4 Hz), 6.24 (s, 2H), 3.26 (s, 2H), 2.75 (s, 2H), 2.25 – 2.14 (m, 2H), 1.03 (s, 2H), 0.91 (dd, 2H, *J* = 13.9, 7.0 Hz), 0.53 (t, 3H, *J* = 7.1 Hz).

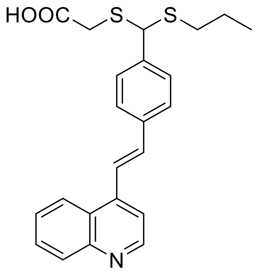

(E)-2-(((propylthio)(4-(2-(quinolin-4-yl)vinyl)phenyl)methyl)thio)acetic acid (**6**)

Compound **26** (129.5 mg, 0.5 mmol) and TsOH·H_2O (570 mg, 0.75 mmol) were dissolved in toluene (10 mL) and 4Å molecular sieves were added. Sodium propane-1-thiolate (196 mg, 2 mmol) and 2-mercaptoacetic acid (70 mg, 1 mmol) were added and the reaction mixture was stirred at reflux for 2.5 h under inert conditions. The reaction mixture was concentrated under reduced pressure. The residue was purified by flash chromatography on a silica gel column (CH_2Cl_2:MeOH:AcOH; 99:1:0.5 to 90:10:0.5) to afford **6** as a yellow solid (13 mg, 6%). ^1H NMR (DMSO-d_6) δ (ppm): 8.89 (d, 1H, *J* = 4.6 Hz), 8.52 (d, 1H, *J* = 8.4 Hz), 8.10 (d, 1H, *J* = 16.1 Hz), 8.04 (d, 1H, *J* = 8.4 Hz), 7.85 (d, 1H, *J* = 4.6 Hz), 7.83 (d, 2H, *J* = 8.1 Hz), 7.81 – 7.78 (t, 1H, *J* = 7.6 Hz), 7.66 (t, 1H, *J* = 7.6 Hz), 7.59 (d, 1H, *J* = 16.1 Hz), 7.48 (d, 2H, *J* = 8.0 Hz), 5.23 (s, 1H), 3.36 (d, 1H, *J* = 15.2 Hz), 3.21 (d, 1H, *J* = 15.2 Hz), 2.67 – 2.61 (m, 1H), 2.56 – 2.51 (m, 1H), 1.63 – 1.50 (m, 2H), 0.91 (t, 3H, *J* = 7.3 Hz). ^{13}C NMR (DMSO-d_6) δ (ppm): 171.7, 151.1, 149.2, 143.0, 141.4, 137.1, 135.4, 130.4, 130.3, 128.9, 128.6, 127.5, 126.7, 125.0, 123.6, 117.6, 53.0, 35.1, 34.7, 23.0, 14.2.

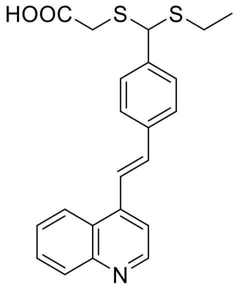

(E)-2-(((ethylthio)(4-(2-(quinolin-4-yl)vinyl)phenyl)methyl)thio)acetic acid (**7**)

Compound **26** (129.5 mg, 0.5 mmol) and TsOH.H_2O (570 mg, 0.75 mmol) were dissolved in toluene (10 mL) and 4Å molecular sieves were added. Sodium ethanethiolate (168 mg, 2 mmol) and 2-mercaptoacetic acid (53 mg, 0.75 mmol) were added and the reaction mixture was stirred at reflux for 2.5 h under inert conditions. The reaction mixture was concentrated

under reduced pressure. The residue was purified by flash chromatography on a silica gel column (CH_2Cl_2:MeOH:AcOH; 99:1:0.5 to 90:10:0.5) to afford **7** as a yellow solid (30 mg, 42%). ^{1}H NMR (DMSO-d_6) δ (ppm): 8.20 (d, 1H, *J* = 5.7 Hz), 7.91 (d, 1H, *J* = 8.6 Hz), 7.39 (d, 1H, *J* = 5.7 Hz), 7.35 (d, 1H, *J* = 8.5 Hz), 7.30 (d, 1H, *J* = 16.0 Hz), 7.13 (t, 1H, *J* = 7.7 Hz), 7.06 (d, 1H, *J* = 16.0 Hz), 6.98 (d, 2H, *J* = 8.1 Hz), 6.97 – 6.94 (t, 1H), 6.55 (d, 2H, *J* = 8.2 Hz), 4.30 (s, 1H), 2.38 (d, 1H, *J* = 15.2 Hz), 2.23 (d, 1H, *J* = 15.2 Hz), 1.68 (tt, 1H, *J* = 14.8, 7.4 Hz), 1.61 – 1.54 (m, 1H), 0.21 (t, 3H, *J* = 7.4 Hz). ^{13}C NMR ($CDCl_3$) δ (ppm): 175.7, 155.6, 149.3, 147.0, 145.3, 144.7, 140.3, 138.5, 133.8, 133.7, 133.0, 130.8, 130.3, 127.3, 125.8, 121.5, 56.6, 38.9, 30.8, 19.3.

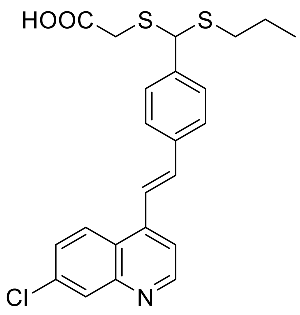

(E)-2-(((4-(2-(7-chloroquinolin-4-yl)vinyl)phenyl)(propylthio)methyl)thio)acetic acid (**8**)

Compound **38** (120 mg, 0.41 mmol) and TsOH.H_2O (467 mg, 2.46 mmol) were dissolved in toluene (10 mL) and 4Å molecular sieves were added. Sodium propane-1-thiolate (161 mg, 1.64 mmol) and 2-mercaptoacetic acid (43 mg, 0.615 mmol) were added and the reaction mixture was stirred at reflux for 2.5 h under inert conditions. The reaction mixture was concentrated under reduced pressure. The residue was purified by flash chromatography on a silica gel column (CH_2Cl_2:MeOH:AcOH; 99:1:0.5 to 90:10:0.5) to afford **8** as yellow crystals (25 mg, 39%). ^{1}H NMR (DMSO-d_6) δ (ppm): δ 9.07 (d, 1H, *J* = 5.2 Hz), 8.78 (d, 1H, *J* = 9.2 Hz), 8.20 (d, 1H, *J* = 1.8 Hz), 8.18 (d, 1H, *J* = 16.2 Hz), 8.14 (d, 1H, *J* = 5.0 Hz), 7.90 (d, 2H, *J* = 8.2 Hz), 7.85 (m, 2H,), 7.51 (d, 2H, *J* = 8.2 Hz), 5.24 (s, 1H), 3.37 (d, 1H, *J* = 15.2 Hz), 3.22 (d, 1H, *J* = 15.2 Hz), 2.69 – 2.61 (m, 1H), 2.57 – 2.52 (m, 1H), 1.62 – 1.50 (m, 2H), 0.91 (t, 3H, *J* = 7.3 Hz). ^{3}C NMR (DMSO-d_6) δ (ppm): ^{13}C NMR (600 MHz, DMSO) δ 171.3, 149.1, 142.0, 138.5, 136.6, 136.2, 128.8, 128.6, 128.5, 127.6, 125.3, 125.0, 122.0, 117.4, 52.6, 34.5, 34.3, 22.6, 13.7.

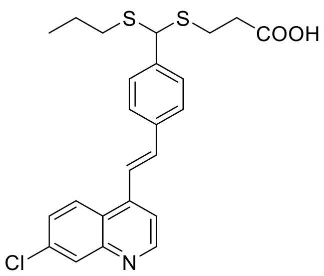

(E)-3-(((4-(2-(7-chloroquinolin-4-yl)vinyl)phenyl)(propylthio)methyl)thio)propanoic acid (**9**)

Compound **38** (100 mg, 0.34 mmol) and TsOH.H_2O (126 mg, 0.68 mmol) were dissolved in toluene (20 mL) and 4Å molecular sieves were added. Sodium propane-1-thiolate (161 mg, 1.64 mmol) and 2-mercaptoacetic acid (43 mg, 0.615 mmol) were added and the reaction mixture was stirred at reflux for 2.5 h under inert conditions. Upon cooling, the material crystalised and was filtered. The precipitate was purified by flash chromatography on a silica gel column (CH_2Cl_2:MeOH:AcOH 99:1:0.5 to 90:10:0.5). The fraction containing the product was then further purified by HPLC reverse phase with a gradient from H_2O:CH_3CN:HCOOH 40:60:0.1 to 60:40:0.1 to afford **9** as yellow crystals (10%). ^1H NMR (DMSO-d_6) δ (ppm): 9.04 (d, 1H, J = 5.0 Hz), 8.75 (d, 1H, J = 9.1 Hz), 8.21 – 8.14 (m, 2H), 8.09 (d, 1H, J = 4.9 Hz), 7.89 (d, 2H, J = 7.9 Hz), 7.85 – 7.77 (m, 2H), 7.54 (d, 2H, J = 8.0 Hz), 5.27 (s, 1H), 2.72 (ddt, 2H, J = 57.4, 13.6, 7.1 Hz), 2.62 – 2.48 (m, 4H), 1.55 (ddt, 2H, J = 14.2, 11.1, 7.0 Hz), 0.91 (t, 3H, J = 7.3 Hz).

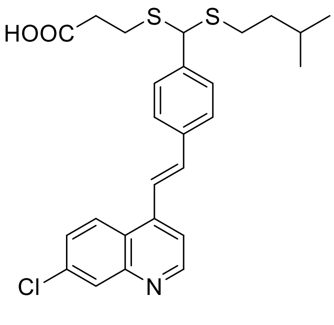

(E)-3-(((4-(2-(7-chloroquinolin-4-yl)vinyl)phenyl)(isopentylthio)methyl)thio)propanoic acid (**10**)

Compound **38** (100 mg, 0.34 mmol) and TsOH.H_2O (126 mg, 0.68 mmol) were dissolved in toluene (25 mL) and 4Å molecular sieves were added. 3-methylbutane-1-thiol (106 mg, 0.85 mmol) and ethyl 3-mercaptopropanoate (107 mg, 0.85 mmol) were added and the reaction mixture was stirred at reflux for 2.5 h under inert conditions. The mixture was diluted with

EtOAc and the organic phase was washed with saturated $NaHCO_{3(aq)}$ and brine, dried over Na_2SO_4, filtered and concentrated under reduced pressure. The residue was purified by flash chromatography on a silica gel column (EtOAc/hexanes). The ester was dissolved in methanol (0.5 mL) and 2N NaOH solution (1 mL) was added. The reaction was stirred at -15 ^{o}C for 40 h. The mixture was diluted with H_2O and purified on a reverse phase Sep-pak column (20 g) (MeOH:H_2O; 60:40) to afford **10** as a white solid (10 mg, 7%). ^{1}H NMR (DMSO-d$_6$) δ (ppm): 8.77 (d, 1H, *J* = 4.7 Hz), 8.38 (d, 1H, *J* = 9.1 Hz), 7.97 (d, 1H, *J* = 2.0 Hz), 7.88 (d, 1H, *J* = 16.2 Hz), 7.78 (d, 1H, *J* = 4.7 Hz), 7.69 (d, 2H, *J* = 8.1 Hz), 7.61 (dd, 1H, *J* = 9.0, 2.0 Hz), 7.49 (d, 1H, *J* = 16.0 Hz), 7.44 (d, 2H, *J* = 8.1 Hz), 5.03 (s, 1H), 2.65 (dt, 1H, *J* = 13.0, 7.7 Hz), 2.59 – 2.54 (m, 1H), 2.45 (tt, 2H, *J* = 12.7, 6.4 Hz), 2.18 (t, 2H, *J* = 7.7 Hz), 1.52 – 1.44 (m, 1H), 1.30 (q, 2H, *J* = 7.4 Hz), 0.74 (d, 3H, *J* = 6.6 Hz), 0.72 (d, 3H, *J* = 6.6 Hz). ^{13}C NMR (DMSO-d$_6$) δ (ppm): 178.0, 152.8, 149.5, 144.7, 143.4, 137.1, 137.0, 136.3, 129.7, 129.2, 129.0, 128.7, 127.6, 125.9, 123.2, 118.7, 53.3, 39.2, 39.0, 31.4, 30.4, 28.3, 23.5, 23.5.

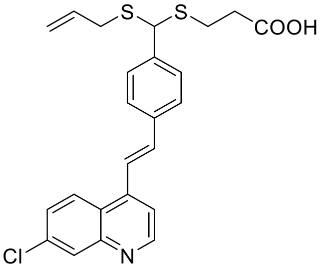

(E)-3-(((allylthio)(4-(2-(7-chloroquinolin-4-yl)vinyl)phenyl)methyl)thio)propanoic acid (**11**)

Compound **38** (100 mg, 0.34 mmol) and TsOH.H_2O (126 mg, 0.68 mmol) were dissolved in toluene (15 mL) and 4Å molecular sieves were added. Allyl thiol (84 µL, 1.02 mmol) and ethyl 3-mercaptopropanoate (129 mg, 1.02 mmol) were added and the reaction mixture was stirred at reflux for 2.5 h under inert conditions. The mixture was diluted with EtOAc and the organic phase was washed with saturated $NaHCO_{3(aq)}$ and brine, dried over Na_2SO_4, filtered and concentrated under reduced pressure. The residue was purified by flash chromatography on a silica gel column (EtOAc:hexanes; 20:80 to 60:40). The ester was dissolved in a solution of H_2O/dioxane (1:1 v/v, 5 mL) and 2N NaOH solution (0.3 mL) was added. The reaction was stirred at 0 ^{o}C for 1 h. The mixture was quenched with 1M HCl and extracted with EtOAc. The solvent was removed under reduced pressure and the residue was purified by flash chromatography on silica gel column (MeOH/H_2O) to afford **11** as yellow crystals (29%).^{1}H

NMR (DMSO-d_6) δ (ppm): 9.10 (d, 1H, *J* = 5.3 Hz), 8.81 (d, 1H, *J* = 9.1 Hz), 8.26 – 8.16 (m, 3H), 7.92 (d, 2H, *J* = 8.0 Hz), 7.90 – 7.85 (m, 2H), 7.54 (d, 2H, *J* = 8.1 Hz), 5.82 (ddt, 1H, *J* = 17.1, 10.1, 7.1 Hz), 5.20 – 5.10 (m, 3H), 3.32 (dd, 1H, *J* = 13.6, 7.3 Hz), 3.18 (dd, 1H, *J* = 13.6, 7.0 Hz), 2.77 (dt, 1H, *J* = 13.8, 7.2 Hz), 2.67 (dt, 1H, *J* = 13.5, 7.1 Hz), 2.57 – 2.48 (m, 2H).

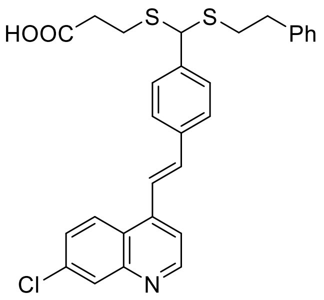

(E)-3-(((4-(2-(7-chloroquinolin-4-yl)vinyl)phenyl)(phenethylthio)methyl)thio)propanoic acid (**LV-320**)

Compound **38** (1.21 g, 4.10 mmol) and TsOH.H_2O (0.86 g, 4.51 mmol) were suspended in CH_2Cl_2 (2.5 mL). 3-mercaptopropionic acid (0.393 mL, 4.51 mmol) and 2-phenylethanethiol (0.604 mL, 4.51 mmol) were added and the reaction was stirred at 50 °C for 1 h. The mixture was diluted with 1:1 water-methanol (4 mL, acidified with a drop of 1 M HCl; add THF drop-wise until the system became homogeneous). The solution was directly loaded onto a C18 cartridge (BioTage KP-C18, 50g), and purified with a 50-100% MeOH-water linear gradient. The pure fractions were combined and concentrated by rotary evaporation to remove MeOH. The resulting precipitate was filtered and dried to give **LV-320** as a white solid (0.75 g, 35%). Purity = 100%; tr = 2.94 min; MS (ESI+): m/z = 520 $[M+H]^+$; HRMS m/z calculated for $C_{29}H_{27}ClNO_2S_2^+$ $[M+H]^+$ 520.1166, found 520.1147. ^1H NMR (DMSO-d_6) δ (ppm): 9.13 (d, 1H, *J* = 5.4 Hz), 8.86 (d, 1H, *J* = 9.2 Hz), 8.31 (d, 1H, *J* = 1.9 Hz), 8.25 (d, 1H, *J* = 5.5 Hz), 8.22 (d, 1H, *J* = 16.1 Hz), 7.95 (d, 1H, *J* = 14.1 Hz), 7.93 (d, 2H, *J* = 8.1 Hz), 7.90 (dd, 1H, *J* = 9.2, 2.0 Hz), 7.54 (d, 2H, *J* = 8.2 Hz), 7.28 (dd, 2H, *J* = 9.7, 5.4 Hz), 7.20 (d, 2H, *J* = 7.2 Hz), 7.22 – 7.18 (t, 1H), 5.29 (s, 1H), 2.88 – 2.81 (m, 2H), 2.79 – 2.72 (m, 2H), 2.69 – 2.63 (m, 3H), 2.53 (t, 1H, *J* = 7.4 Hz). ^{13}C NMR (DMSO-d_6) δ (ppm): 173.4, 147.5, 143.1, 140.8, 140.0, 137.6, 135.9, 129.2, 129.1, 129.0, 128.8, 128.6, 127.9, 126.7, 125.1, 121.5, 117.4, 52.1, 35.6, 34.7, 33.8, 27.6. HRMS: calcd for $C_{29}H_{26}ClNO_2S_2$ 519.1093, observed 520.1204 (ESI+, $[M+H]^+$), 518.1009 (ESI-, $[M-H]^-$); Elemental analysis: calcd for $C_{29}H_{26}ClNO_2S_2$: C 66.97, H 5.04, N 2.69, S 12.33, Cl 6.82; found C 66.74, H 5.00, N 2.79, S 12.10, Cl 6.89.

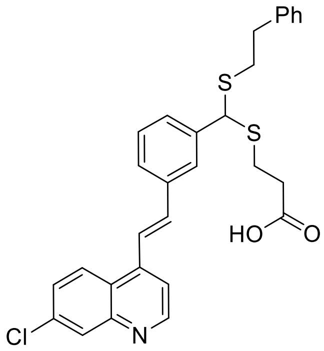

(E)-3-(((3-(2-(7-chloroquinolin-4-yl)vinyl)phenyl)(phenethylthio)methyl)thio)propanoic acid (**12**)
Compound **39** (40 mg, 0.136 mmol) and TsOH.H_2O (5.2 mg, 0.2 mmol) were dissolved in toluene (1 mL) and 4Å molecular sieves were added. 2-phenylethane-1-thiol (12 mg, 0.09 mmol) and ethyl 3-mercaptopropanoate (11.4 mg, 0.09 mmol) were added and the reaction mixture was stirred at reflux for 2.5 h under inert conditions. The mixture was diluted with EtOAc and the organic phase was washed with saturated $NaHCO_{3(aq)}$ and brine, dried over Na_2SO_4, filtered and concentrated under reduced pressure. The residue was purified by flash chromatography on a silica gel column (EtOAc/hexanes). The ester was dissolved in a solution of H_2O/dioxane (1:1 v/v, 5 mL) and 2N NaOH solution (0.3 mL) was added. The reaction was stirred at -15 ^oC for 24 h. The mixture was quenched with 1M HCl and extracted with EtOAc. The solvent was removed under reduced pressure and the residue was purified by flash chromatography on a C18 reverse phase column (CH_3CN:H_2O;0:100 to 50:50) to afford **12** as white crystals (5 mg, 7%). ^1H NMR (DMSO-d_6) δ (ppm): 8.92 (d, 1H, *J* = 4.6 Hz), 8.61 (d, 1H, *J* = 9.0 Hz), 8.09 (t, 2H, *J* = 8.0 Hz), 7.93 (d, 1H, *J* = 4.6 Hz), 7.85 (s, 1H), 7.78 (d, 1H, *J* = 6.1 Hz), 7.69 (d, 1H, *J* = 9.0 Hz), 7.64 (d, 1H, *J* = 16.0 Hz), 7.47 – 7.41 (m, 2H), 7.25 (t, 2H, *J* = 7.4 Hz), 7.20 (d, 2H, *J* = 7.5 Hz), 7.17 (t, 1H, *J* = 7.2 Hz), 5.22 (s, 1H), 2.83 (t, 3H, *J* = 10.2 Hz), 2.76 – 2.68 (m, 2H), 2.68 – 2.60 (m, 2H), 2.08 (t, 2H, *J* = 7.9 Hz). ^{13}C NMR (DMSO-d_6) δ (ppm): 173.6, 152.1, 149.3, 143.0, 142.5, 140.9, 136.9, 136.0, 134.6, 129.4, 129.0, 128.8, 128.6, 128.5, 127.5, 127.1, 127.1, 126.7, 125.0, 122.7, 117.6, 52.5, 39.3, 35.8, 33.9, 30.3.

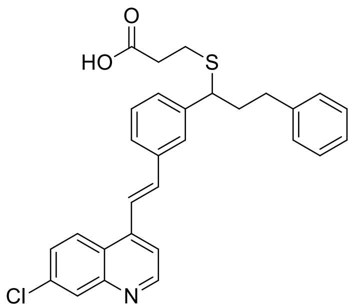

(E)-3-((1-(3-(2-(7-chloroquinolin-4-yl)vinyl)phenyl)-3-phenylpropyl)thio)propanoic acid (**13**)

To a solution (THF/EtOH 2/1, 0.22 mL) of **58** (0.051 g, 0.10 mmol, 1.0 eq.) was added aqueous NaOH (2M, 0.15 mL). After stirring 72 h at room temperature, the mixture was neutralized with aqueous 1M HCl until pH = 5 and extracted two times with CH_2Cl_2. The organic layers were pooled, dried over Na_2SO_4 and concentrated under reduced pressure to give a yellow oil (0.031 g). The crude product was purified by chromatography on 4 g C18 column (H_2O:MeOH; 95:5 to 0:10) to afford after lyophilization **13** as a white amorphous solid (0.011 g, 23%). Purity = 98%; tr = 3.00 min; MS (ESI+): m/z = 488 $[M+H]^+$; HRMS m/z calculated for $C_{29}H_{27}ClNO_2S^+$ $[M+H]^+$ 488.1451, found 488.1470. ^1H NMR (DMSO-d_6) δ (ppm): 8.92 (d, 1H, *J* = 5.0 Hz), 8.64 (d, 1H, *J* = 9.0 Hz), 8.10 (d, 1H, *J* = 16.0 Hz), 8.09 (d, 1H, *J* = 2.0 Hz), 7.92 (d, 1H, *J* = 5.0 Hz), 7.78 (s, 1H), 7.76 (d, 1H, *J* = 7.5 Hz), 7.69 (dd, 1H, *J* = 2.0 and 9.0 Hz), 7.66 (d, 1H, *J* = 16.0 Hz), 7.43 (t, 1H, *J* = 7.5 Hz), 7.34 (d, 1H, *J* = 7.5 Hz), 7.30-7.27 (m, 2H), 7.19-7.09 (m, 3H), 3.88 (t, 1H, *J* = 7.5 Hz), 2.68-2.62 (m, 1H), 2.57-2.53 (m, 1H), 2.45 (t, 2H, *J* = 8.0 Hz), 2.20-2.14 (m, 2H), 2.10-1.99 (m, 2H). ^{13}C NMR (DMSO-d_6) δ (ppm): 174.8, 152.0, 149.3, 144.2, 143.1, 141.8, 136.8, 136.3, 134.5, 129.3, 128.8, 128.7, 128.6, 128.4, 127.6, 127.4, 127.1, 126.3, 124.9, 122.4, 117.5, 48.6, 38.9, 38.0, 33.7, 28.5.

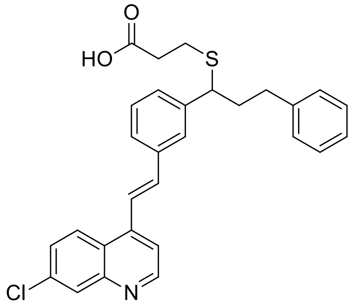

(+)-(E)-3-((1-(3-(2-(7-chloroquinolin-4-yl)vinyl)phenyl)-3-phenylpropyl)thio)propanoic acid ((**+**)-**13**)

Obtained according to general procedure G from **(+)-58**. Yellow oil (0.077 g, 52%). Purity = 98%; tr = 2.99 min; MS (ESI+): m/z = 488 $[M+H]^+$; HRMS m/z calculated for $C_{29}H_{27}ClNO_2S^+$ $[M+H]^+$ 488.1451, found 488.1464. ^1H NMR ($CDCl_3$) δ (ppm): 8.71 (d, 1H, *J* = 4.5 Hz), 8.00-7.97 (m, 2H), 7.72 (s, 1Hz), 7.63 (d, 1H, *J* = 16.5 Hz), 7.51-7.45 (m, 2H), 7.40-7.28 (m, 6H + $CDCl_3$), 7.23-7.20 (m, 3H), 3.94 (t, 1H, *J* = 7.5 Hz), 2.78-2.70 (m, 4H), 2.60-2.48 (m, 2H), 2.34-2.24 (m, 2H). ^{13}C NMR ($CDCl_3$) δ (ppm): 175.4, 149.6, 147.1, 144.0, 143.4, 141.2, 136.3, 136.3, 135.8, 129.4, 128.6, 128.5, 127.6, 127.1, 127.0, 126.5, 126.1, 125.1, 124.4, 121.7, 116.5, 49.3, 37.7, 34.8, 33.7, 26.2. $[\alpha]_{20}^{D}$ = + 0.56° (c = 0.5 g/100 mL in MeOH/DMSO (1:1)).

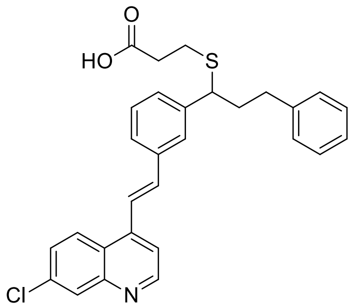

(-)-(E)-3-((1-(3-(2-(7-chloroquinolin-4-yl)vinyl)phenyl)-3-phenylpropyl)thio)propanoic acid ((-)-**13**)

Obtained according to general procedure G from **(-)-58**. Yellow oil (0.077 g, 52%). Purity = 99%; tr = 3.00 min; MS (ESI+): m/z = 488 $[M+H]^+$; HRMS m/z calculated for $C_{29}H_{27}ClNO_2S^+$ $[M+H]^+$ 488.1451, found 488.1461. ^1H NMR ($CDCl_3$) δ (ppm): 8.71 (br s, 1H), 8.00-7.98 (m, 2H), 7.72 (s, 1Hz), 7.63 (d, 1H, *J* = 16.0 Hz), 7.51-7.45 (m, 2H), 7.40-7.29 (m, 6H + $CDCl_3$), 7.23-7.20 (m, 3H), 3.94 (t, 1H, *J* = 7.5 Hz), 2.80-2.70 (m, 4H), 2.60-2.47 (m, 2H), 2.34-2.22 (m, 2H). ^{13}C NMR ($CDCl_3$) δ (ppm): 175.4, 149.6, 147.1, 144.0, 143.4, 141.2, 136.3, 136.3, 135.8, 129.4, 128.6, 128.5, 127.6, 127.1, 127.0, 126.5, 126.1, 125.1, 124.4, 121.7, 116.5, 49.3, 37.7, 34.8, 33.7, 26.2. $[\alpha]_{20}^{D}$ = - 0.41° (c = 0.5 g/100 mL in MeOH/DMSO (1:1)).

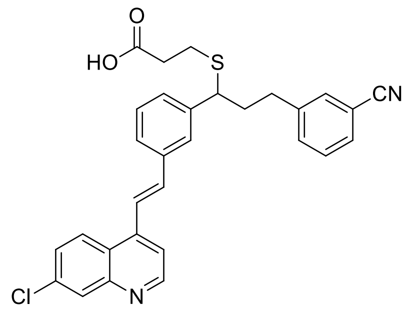

(E)-3-((1-(3-(2-(7-chloroquinolin-4-yl)vinyl)phenyl)-3-(3-cyanophenyl)propyl)thio)propanoic acid (**14**)

Obtained according to general procedure G from **59**. Yellow oil (0.171 g, 82%). Purity = 92%; tr = 2.88 min; MS (ESI+): m/z = 513 $[M+H]^+$; HRMS m/z calculated for $C_{30}H_{26}ClN_2O_2S^+$ $[M+H]^+$ 513.1398, found 513.1409. ^1H NMR ($CDCl_3$) δ (ppm): 8.71 (d, 1H, *J* = 4.4 Hz), 8.00 (d, 1H, *J* = 2.0 Hz), 7.98 (d, 1H, *J* = 9.0 Hz), 7.71 (s, 1H), 7.64 (d, 1H, *J* = 16.1 Hz), 7.56-7.30 (m, 10H), 3.92 (t, 1H, *J* = 7.4 Hz), 2.87-2.66 (m, 4H), 2.63-2.45 (m, 2H), 2.30-2.25 (m, 2H). ^{13}C NMR ($CDCl_3$) δ (ppm): 175.2, 149.5, 147.0, 144.2, 143.0, 142.6, 136.5, 136.2, 135.9, 133.0, 132.0, 129.9, 129.5, 129.3, 128.5, 127.6, 127.2, 126.9, 126.5, 125.1, 124.4, 121.9, 118.9, 116.6, 112.5, 49.2, 37.5, 34.8, 33.3, 26.2.

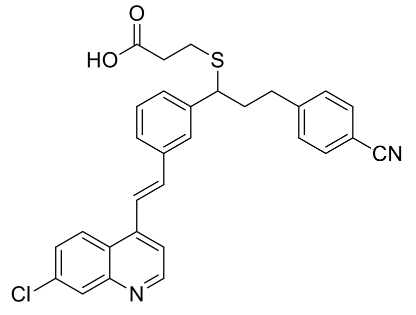

(E)-3-((1-(3-(2-(7-chloroquinolin-4-yl)vinyl)phenyl)-3-(4-cyanophenyl)propyl)thio)propanoic acid (**15**)

Obtained according to general procedure G from **60**. White solid (0.054 g, 25%). Purity = 92%; tr = 3.17 min; MS (ESI+): m/z = 513 $[M+H]^+$; HRMS m/z calculated for $C_{30}H_{26}ClN_2O_2S^+$ $[M+H]^+$ 513.1398, found 513.1384. ^1H NMR ($CDCl_3$) δ (ppm): 8.75 (d, 1H, *J* = 3.9 Hz), 8.14 (s, 1H), 8.05 (d, 1H, *J* = 9.4 Hz), 7.73 (s, 1H), 7.69 (d, 1H, *J* = 15.9 Hz), 7.60 (d, 2H, *J* = 8.1 Hz), 7.54 (d, 1H, *J* = 8.0 Hz), 7.49-7.47 (m, 2H), 7.43 (d, 1H, *J* = 8.8 Hz), 7.41–7.35 (m, 2H), 7.31 (d, 2H, *J* = 8.1 Hz), 3.92 (t, 1H, *J* = 7.4 Hz), 2.85–2.72 (m, 4H), 2.59-2.50 (m, 2H), 2.31–2.24

(m, 2H). ^{13}C NMR ($CDCl_3$) δ (ppm): 174.0, 146.3, 145.5, 142.6, 137.0, 135.7, 134.4, 131.9, 131.8, 129.6, 129.1, 128.8, 128.8, 128.3, 127.7, 127.0, 126.1, 124.7, 123.9, 121.1, 118.5, 115.9, 109.6, 48.7, 36.8, 34.1, 33.4, 25.7.

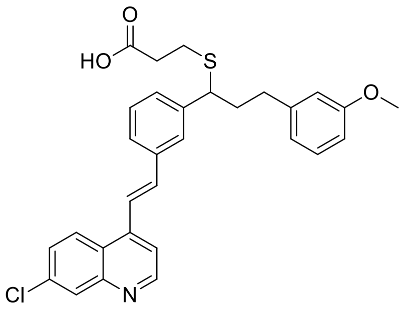

(E)-3-((1-(3-(2-(7-chloroquinolin-4-yl)vinyl)phenyl)-3-(3-methoxyphenyl)propyl)thio)propanoic acid (**16**)

Obtained according to general procedure G from **61**. Yellow oil (0.162 g, 69%). Purity = 91%; tr = 3.03 min; MS (ESI+): m/z = 518 $[M+H]^+$; HRMS m/z calculated for $C_{30}H_{29}ClNO_3S^+$ $[M+H]^+$ 518.1551, found 518.1559. ^{1}H NMR ($CDCl_3$) δ (ppm): 8.70 (d, 1H, *J* = 4.8 Hz), 8.02 (d, 1H, *J* = 1.5 Hz), 8.00 (d, 1H, *J* = 9.0 Hz),7.73 (s, 1H), 7.64 (d, 1H, *J* = 16.1 Hz), 7.55-7.43 (m, 2H), 7.43-7.18 (m, 6H + $CDCl_3$), 6.82-6.74 (m, 3H), 3.94 (t, 1H, *J* = 7.4 Hz), 3.81 (s, 3H), 2.78-2.53 (m, 5H), 2.53-2.45 (m, 1H), 2.28 (m, 2H). ^{13}C NMR ($CDCl_3$) δ (ppm): 175.3, 159.7, 149.4, 146.9, 144.3, 143.4, 142.8, 136.5, 136.3, 135.9, 129.4, 129.4, 128.7, 127.7, 127.1, 126.9, 126.5, 125.1, 124.4, 121.7, 120.9, 116.5, 114.3, 111.3, 55.2, 49.3, 37.6, 34.8, 33.7, 26.2.

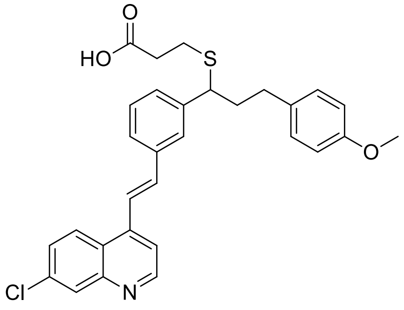

(E)-3-((1-(3-(2-(7-chloroquinolin-4-yl)vinyl)phenyl)-3-(4-methoxyphenyl)propyl)thio)propanoic acid (**17**)

Obtained according to general procedure G from **62** as a white solid (0.086 g, 44%). Purity = 92%; tr = 3.17 min; MS (ESI+): m/z = 518 $[M+H]^+$; HRMS m/z calculated for $C_{30}H_{29}ClNO_3S^+$

$[M+H]^+$ 518.1551, found 518.1536. ^1H NMR ($CDCl_3$) δ (ppm): 8.70 (d, 1H, *J* = 4.5 Hz), 8.01-7.98 (m, 2H), 7.72 (s, 1H), 7.63 (d, 1H, *J* = 16.0 Hz), 7.51-7.45 (m, 2H), 7.40-7.34 (m, 3H), 7.31 (d, 1H, *J* = 16.0 Hz), 7.12 (d, 2H, *J* = 8.6 Hz), 6.86 (d, 2H, *J* = 8.6 Hz), 3.93 (s, 1H), 3.81 (s, 3H), 2.82-2.62 (m, 4H), 2.59-2.44 (m, 2H), 2.33-2.20 (m, 2H). ^{13}C NMR ($CDCl_3$) δ (ppm): 174.4, 157.5, 148.4, 145.9, 144.4, 143.0, 136.4, 135.7, 132.7, 128.9, 128.9, 128.8, 128.7, 128.3, 127.4, 126.7, 126.1, 124.7, 123.9, 121.1, 115.9, 113.4, 54.8, 48.7, 37.4, 34.2, 32.2, 25.6.

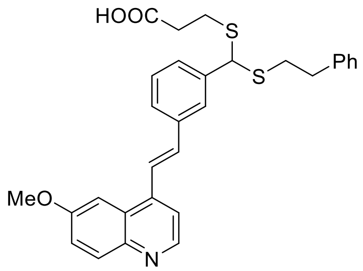

(E)-3-(((3-(2-(6-methoxyquinolin-4-yl)vinyl)phenyl)(phenethylthio)methyl)thio)propanoic acid (**18**)

Compound **41** (100 mg, 0.271 mmol) and 6-methoxy-4-methylquinoline (93 mg, 0.542 mmol) were dissolved in acetic anhydride (0.35 mL). The reaction mixture was stirred under microwave irraditation at 180 °C for 4 h. The mixture was diluted with EtOAc and the organic phase was washed with saturated $NaHCO_{3(aq)}$ and brine, dried over Na_2SO_4, filtered and concentrated under reduced pressure. The residue was purified by flash chromatography on a silica gel column (EtOAc:hexane; 10:90 to 50:50). The ester was dissolved in MeOH (0.5 mL) and 30N NaOH solution (25 µL) was added. The reaction was stirred at -15 °C for 48 h. The mixture was quenched with 1M HCl and extracted with EtOAc. The solvent was removed under reduced pressure and the residue was purified by reverse phase chromatography to afford **18** as pale yellow crystals (14 mg, 10%). ^1H NMR (DMSO-d_6) δ (ppm): 8.73 (d, 1H, *J* = 4.6 Hz), 8.06 (d, 1H, *J* = 16.1 Hz), 7.96 (d, 1H, *J* = 9.2 Hz), 7.84 (t, 2H, *J* = 4.5 Hz), 7.80 (s, 1H), 7.73 (d, 1H, *J* = 2.7 Hz), 7.58 (d, 1H, *J* = 16.1 Hz), 7.48 – 7.42 (m, 3H), 7.28 – 7.16 (m, 5H), 5.24 (s, 1H), 3.99 (s, 3H), 2.88 – 2.79 (m, 3H), 2.77 – 2.62 (m, 3H), 2.10 (dd, 2H, *J* = 15.6, 7.8 Hz).

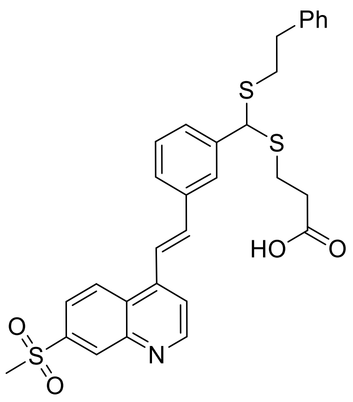

(E)-3-(((3-(2-(7-(methylsulfonyl)quinolin-4-yl)vinyl)phenyl)(phenethylthio)methyl)thio)propanoic acid (**19**)

4-methyl-7-(methylsulfonyl)quinoline (35 mg, 0.158 mmol, 1 eq.), isophtaldehyde (61.7 mg, 0.158 mmol) and zinc chloride (6.4 mg, 0.047 mmol) were dissolved in NMP (0.2 mL) and the mixture was stirred under microwave irradiation at 150 °C for 3 h. The mixture was diluted with EtOAc and the organic phase was washed with saturated $NaHCO_{3(aq)}$ and brine, dried over Na_2SO_4, filtered and concentrated under reduced pressure. The residue was purified by flash chromatography on a silica gel column (MeOH:CH_2Cl_2; 1:99 to 5:95) to afford *(E)*-3-(2-(7-(methylsulfonyl)quinolin-4-yl)vinyl)benzaldehyde (**40**) as yellow crystals (20 mg, 38%) which was used directly in the next reaction.

Compound **40** (15 mg, 0.045 mmol) was dissolved in 1,1,2,2-tetrachloroethane (0.5 mL) and TFA (100 μL). 2-phenylethane-1-thiol (6 mg, 0.045 mmol) and ethyl 3-mercaptopropanoate (6 mg, 0.045 mmol) were added and the reaction mixture was stirred at rt for 6 h. The mixture was diluted with EtOAc and the organic phase was washed with saturated $NaHCO_{3(aq)}$ and brine, dried over Na_2SO_4, filtered and concentrated under reduced pressure. The residue was purified by flash chromatography on a silica gel column (EtOAc:hexane; 15:85 to 70:30). The ester was dissolved in MeOH (0.5 mL) and 30N NaOH solution (80 μL) was added. The reaction was stirred at -15 ^{o}C for 16 h. The mixture was quenched with 1M HCl and extracted with EtOAc. The solvent was removed under reduced pressure and the residue was purified by reverse phase chromatography (CH_3CN:H_2O:formic acid; 40:60:0.1 to 80:20:0.1) to afford **19** as white crystals (20%). ^{1}H NMR (DMSO-d_6) δ (ppm): 9.06 (d, 1H, *J* = 4.6 Hz), 8.86 (d, 1H, *J* = 8.8 Hz), 8.55 (d, 1H, *J* = 1.8 Hz), 8.14 (d, 1H, *J* = 16.1 Hz), 8.09 (m, 2H), 7.86 (s, 1H), 7.81 (d, 1H, *J* = 6.6 Hz), 7.70 (d, 1H, *J* = 16.0 Hz), 7.45 (s, 1H), 7.45 (d, 1H, *J* = 3.0 Hz), 7.25 (t, 2H, *J* = 7.4 Hz), 7.21 (d, 2H, *J* = 7.1 Hz), 7.17 (t, 1H, *J* = 7.3 Hz), 5.24 (s, 1H), 3.38 (s, 3H), 2.83 (t,

2H, *J* = 9.7 Hz), 2.76 – 2.69 (m, 1H), 2.67 (dd, 1H, *J* = 7.7, 4.5 Hz), 2.62 (dt, 2H, *J* = 12.7, 7.8 Hz), 2.07 (t, 2H, *J* = 7.8 Hz). ^{13}C NMR (DMSO-d_6) δ (ppm): 173.2, 152.7, 147.7, 143.1, 142.5, 141.7, 140.9, 136.8, 136.5, 129.6, 129.4, 129.0, 128.8, 128.7, 128.7, 128.7, 127.6, 127.1, 126.6, 123.1, 122.5, 119.5, 52.4, 43.8, 35.7, 33.9, 30.3.

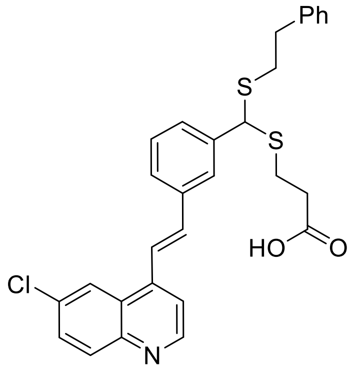

(E)-3-(((3-(2-(6-chloroquinolin-4-yl)vinyl)phenyl)(phenethylthio)methyl)thio)propanoic acid (**20**) Compound **41** (65 mg, 0.16 mmol) and 6-methoxy-4-methylquinoline (57 mg, 0.32 mmol) were dissolved in acetic anhydride (1 mL). The reaction mixture was stirred under microwave irraditation at 150 °C for 6 h. The mixture was diluted with EtOAc and the organic phase was washed with saturated $NaHCO_{3(aq)}$ and brine, dried over Na_2SO_4, filtered and concentrated under reduced pressure. The residue was purified by flash chromatography on a silica gel column (EtOAc:hexane; 25:75 to 55:45). The ester was dissolved in MeOH (0.5 mL) and 30N NaOH solution (50 µL) was added. The reaction was stirred at -15 °C for 24 h. The mixture was quenched with 1M HCl and extracted with EtOAc. The solvent was removed under reduced pressure and the residue was purified by reverse phase C18 chromatography to ($CH_3CN:H_2O$; 50:50) afford **20** as white crystals (3 mg, 4%). ^1H NMR (DMSO-d_6) δ (ppm): 8.90 (d, 1H, *J* = 4.6 Hz), 8.66 (s, 1H), 8.10 (d, 1H, *J* = 16.0 Hz), 8.05 (d, 1H, *J* = 9.0 Hz), 7.96 (d, 1H, *J* = 4.6 Hz), 7.86 (d, 1H, *J* = 5.8 Hz), 7.83 (s, 1H), 7.80 (d, 1H, *J* = 9.1 Hz), 7.63 (d, 1H, *J* = 15.9 Hz), 7.44 (s, 1H), 7.43 (d, 1H, *J* = 6.1 Hz), 7.25 (t, 2H, *J* = 7.4 Hz), 7.20 (d, 2H, *J* = 7.7 Hz), 7.17 (t, 1H, *J* = 7.2 Hz), 5.22 (s, 1H), 2.82 (t, 4H, *J* = 7.8 Hz), 2.68 (m, 2H), 2.05 (t, 2H, *J* = 7.9 Hz). ^{13}C NMR (DMSO-d_6) δ (ppm): 151.2, 147.3, 142.5, 142.3, 141.0, 137.0, 136.0, 132.2, 132.0, 130.5, 129.4, 129.0, 128.8, 128.5, 127.9, 126.7, 123.8, 122.7, 118.1, 52.5, 35.8, 33.9, 30.4.

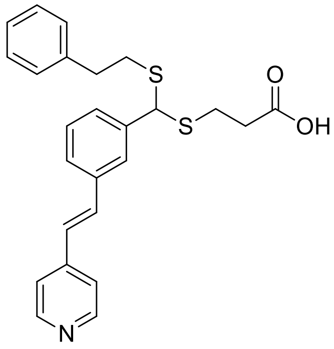

(E)-3-(((phenethylthio)(3-(2-(pyridin-4-yl)vinyl)phenyl)methyl)thio)propanoic acid (**21**)

Obtained according to general procedure B from **63**. White solid (0.057 g, 33%). Purity = 97%; tr = 2.62 min; MS (ESI+): m/z = 436 $[M+H]^+$; HRMS m/z calculated for $C_{25}H_{26}NO_2S_2^+$ $[M+H]^+$ 436.1399, found 436.1412. ^1H NMR (DMSO-d_6) δ (ppm): 8.56 (dd, 2H, *J* = 1.6 and 4.6 Hz), 7.71 (s, 1H), 7.65–7.53 (m, 4H), 7.41 (dd, 2H, *J* = 1.6 and 4.6 Hz), 7.31–7.13 (m, 6H), 5.25 (s, 1H), 2.90–2.60 (m, 6H), 2.58–2.45 (m, 2H + DMSO-d_6). ^{13}C NMR (DMSO-d_6) δ (ppm): 173.4, 150.5, 144.6, 141.9, 140.7, 136.9, 133.1, 129.5, 129.0, 128.8, 128.3, 126.9, 126.7, 121.4, 52.3, 35.6, 34.7, 33.8, 27.6.

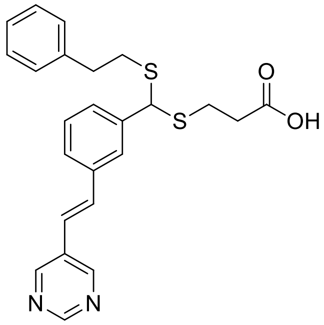

(E)-3-(((phenethylthio)(3-(2-(pyrimidin-5-yl)vinyl)phenyl)methyl)thio)propanoic acid (**22**)

Obtained according to general procedure B from **64**. White solid (0.070 g, 42%). Purity = 99%; tr = 2.57 min; MS (ESI+): m/z = 437 $[M+H]^+$; HRMS m/z calculated for $C_{24}H_{25}N_2O_2S_2^+$ $[M+H]^+$ 437.1352, found 437.1348. ^1H NMR (DMSO-d_6) δ (ppm): 12.33 (s, 1H), 9.08 (d, 3H, *J* = 2.3 Hz), 7.69 (s, 1H), 7.59-7.56 (m, 2H), 7.44-7.39 (m, 2H), 7.29-7.24 (m, 3H), 7.22–7.17 (m, 3H), 5.25 (s, 1H), 2.89–2.72 (m, 5H), 2.69–2.63 (m, 1H), 2.57–2.46 (m, 2H + DMSO-d_6). ^{13}C NMR (DMSO-d_6) δ (ppm): 173.4, 157.4, 154.9, 141.8, 137.1, 132.4, 131.2, 129.5, 128.2, 126.7, 126.4, 122.5, 52.4, 34.6, 27.6.

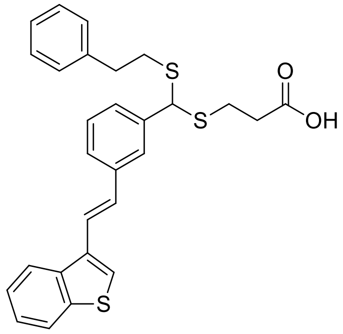

(E)-3-(((3-(2-(benzo[b]thiophen-3-yl)vinyl)phenyl)(phenethylthio)methyl)thio)propanoic acid (**23**)

Obtained according to general procedure C from **65**. White solid (0.054 g, 23%). Purity = 90%; tr = 3.13 min; MS (ESI-): m/z = 489 [M-H]$^-$; HRMS m/z calculated for $C_{28}H_{25}O_2S_3^-$ [M-H]$^-$ 489.1022, found 489.1036. ^{1}H NMR (DMSO-*d$_6$*) δ (ppm): 8.24 (d, 1H, *J* = 7.6 Hz), 8.09 (s, 1H), 8.04 (d, 1H, *J* = 7.4 Hz), 7.71 (s, 1H), 7.67 (d, 1H, *J* = 7.4 Hz), 7.61 (d, 1H, *J* = 16.5 Hz), 7.50-7.34 (m, 6H), 7.29-7.25 (m, 2H), 7.23-7.17 (m, 2H), 5.25 (s, 1H), 2.89–2.81 (m, 2H), 2.80-2.62 (m, 4H), 2.59–2.42 (m, 2H + DMSO). ^{13}C NMR (DMSO-*d$_6$*) δ (ppm): 173.4, 141.7, 140.8, 140.3, 137.9, 137.8, 133.9, 129.9, 129.4, 129.0, 128.8, 127.3, 126.7, 126.3, 126.2, 125.2, 125.0, 123.6, 123.5, 122.7, 121.3, 52.4, 35.6, 34.9, 33.8, 27.7.

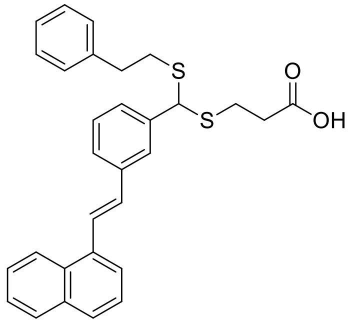

(E)-3-(((3-(2-(naphthalen-1-yl)vinyl)phenyl)(phenethylthio)methyl)thio)propanoic acid (**24**)

Obtained according to general procedure B from **66**. White solid (0.018 g, 5%). Purity = 97%; tr = 3.12 min; MS (ESI-): m/z = 483 [M-H]$^-$; HRMS m/z calculated for $C_{30}H_{27}O_2S_2^-$ [M-H]$^-$ 483.1458, found 483.1452. ^{1}H NMR (CD_3CN) δ (ppm): 8.35 (d, 1H, *J* = 8.2 Hz), 8.04 (d, 1H, *J* = 16.1 Hz), 7.98–7.94 (m, 1H), 7.89 (t, 2H, *J* = 8.4 Hz), 7.73 (s, 1H), 7.68–7.54 (m, 4H), 7.46–7.39 (m, 2H), 7.38-7.21 (m, 6H), 5.06 (s, 1H), 2.96–2.70 (m, 6H), 2.69–2.49 (m, 2H). ^{13}C NMR (CD_3CN) δ (ppm): 173.7, 142.9, 142.0, 139.4, 136.1, 135.2, 132.6, 132.5, 130.5, 130.0, 129.9,

129.8, 129.5, 128.5, 127.7, 127.7, 127.6, 127.4, 127.3, 127.2, 125.1, 124.9, 54.0, 36.9, 35.2, 35.1, 28.7.

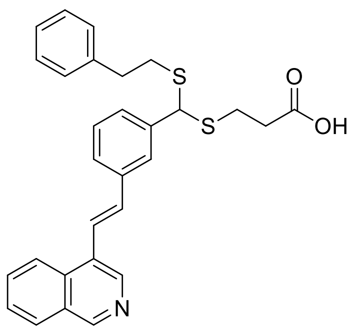

(E)-3-(((3-(2-(isoquinolin-4-yl)vinyl)phenyl)(phenethylthio)methyl)thio)propanoic acid (**25**)
Obtained according to general procedure B from **67**. White solid (0.055 g, 21%). Purity = 98%; tr = 2.71 min; MS (ESI+): m/z = 486 $[M+H]^+$; HRMS m/z calculated for $C_{29}H_{28}NO_2S_2^+$ $[M+H]^+$ 486.1556, found 486.1548. ^1H NMR (DMSO-*d*$_6$) δ (ppm): 9.27 (s, 1H), 8.89 (s, 1H), 8.43 (d, 1H, *J* = 8.1 Hz), 8.18 (d, 1H, *J* = 8.1 Hz), 7.98 (d, 1H, *J* = 16.3 Hz), 7.88 (ddd, 1H, *J* = 8.3, 6.9 and 1.2 Hz), 7.80–7.77 (m, 3H), 7.48–7.39 (m, 3H), 7.36–7.07 (m, 5H), 5.27 (s, 1H), 2.90–2.64 (m, 6H), 2.56 (t, 2H, *J* = 6.9 Hz). ^{13}C NMR (DMSO-*d*$_6$) δ (ppm): 172.9, 151.7, 141.3, 140.3, 140.0, 137.2, 132.9, 132.2, 130.8, 129.0, 128.5, 128.3, 128.1, 127.8, 127.7, 127.5, 127.2, 126.3, 126.2, 123.0, 122.6, 51.9, 35.2, 34.2, 33.3, 27.2.

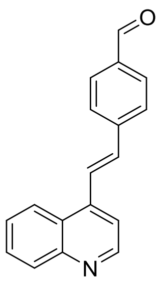

(E)-4-(2-(quinolin-4-yl)vinyl)benzaldehyde (**26**)
Lepidine (1 mL, 7.54 mmol), terephthalaldehyde (3.04 g, 22.7 mmol) and $ZnCl_2$ (463 mg, 3.41 mmol) were combined and stirred under microwave irradiation at 150 ^oC for 20 min. The mixture was then dissolved in DMF, diluted with EtOAc and the organic phase was washed with saturated $NaHCO_3$ and brine, dried over Na_2SO_4, filtered and concentrated under reduced pressure. The residue was purified by flash chromatography on a silica gel column (EtOAc:hexane; 20:80 to 75:25) to afford **26** as a white solid (1 g, 51%). ^1H NMR (DMSO-d$_6$) δ

(ppm): 10.04 (s, 1H), 8.92 (d, 1H, *J* = 4.6 Hz), 8.56 (d, 1H, *J* = 8.4 Hz), 8.30 (d, 1H, *J* = 16.2 Hz), 8.06 (m, 3H), 7.97 (m, 2H,), 7.89 (d, 1H, *J* = 4.6 Hz), 7.80 (m, 1H), 7.69 (d, 1H, *J* = 16.1 Hz), 7.68 (t, 1H, *J* = 7.5 Hz).

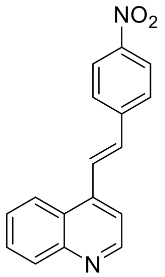

(E)-4-(4-nitrostyryl)quinoline (**28**)

Lepidine (0.5 mL, 3.79 mmol), 4-nitrobenzaldehyde (1.14 g, 7.57 mmol) and $ZnCl_2$ (231 mg, 1.70 mmol) were combined and stirred under microwave irradiation at 150 ^oC for 20 min. The resultant solid was triturated in boiling MeOH and filtered to give **28** as a yellow powder (1.04 g, quant). ^1H NMR (DMSO-d_6) δ (ppm): 8.94 (d, 1H, *J* = 4.6 Hz), 8.58 (d, 1H, *J* = 8.3 Hz), 8.36 (d, 1H, *J* = 16.2 Hz), 8.29 (d, 2H, *J* = 8.8 Hz), 8.13 (t, 2H, *J* = 9.3 Hz), 8.07 (d, 1H, *J* = 8.3 Hz), 7.91 (d, 1H, *J* = 4.6 Hz), 7.84 – 7.80 (m, 1H), 7.75 (d, 1H, *J* = 16.2 Hz), 7.70 (dd, 1H, *J* = 11.2, 4.0 Hz). ^{13}C NMR (DMSO-d_6) δ (ppm): 150.8, 148.8, 147.5, 143.6, 141.9, 133.4, 130.1, 130.1, 129.0, 127.6, 127.3, 126.3, 124.7, 124.5, 117.7.

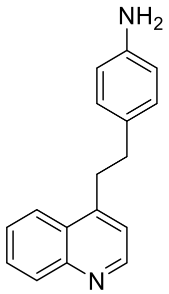

4-(2-(quinolin-4-yl)ethyl)aniline (**29**)

Compound **28** (0.8 g, 2.85 mmol) was dissolved in DMF (20 mL) and AcOH (150 µL). Pd/C (10%, 80 mg, 0.28 mmol) was added, the reaction was put under an atmosphere of H_2 and stirred at rt for 24 h. The mixture was diluted in EtOAc and washed with saturated $NaHCO_3$ and brine. The organic phase was dried over Na_2SO_4, filtered and concentrated under

reduced pressure. The residue was purified by flash chromatography on a silica gel column (EtOAc:hexane; 50:50 to 100:0) to afford **29** (400 mg, 57%). ^{1}H NMR (DMSO-d_6) δ (ppm): 8.76 (d, 1H, *J* = 4.3 Hz), 8.19 (d, 1H, *J* = 8.3 Hz), 8.02 (d, 1H, *J* = 8.3 Hz), 7.75 (t, 1H, *J* = 7.5 Hz), 7.63 (t, 1H, *J* = 7.5 Hz), 7.32 (d, 1H, *J* = 4.2 Hz), 6.92 (d, 2H, *J* = 8.0 Hz), 6.49 (d, 2H, *J* = 8.1 Hz), 4.86 (s, 2H), 3.32 – 3.24 (m, 2H), 2.87 – 2.79 (m, 2H). ^{13}C NMR (DMSO-d_6) δ (ppm): 150.6, 148.3, 148.0, 147.2, 130.2, 129.5, 129.3, 128.5, 127.5, 126.9, 124.4, 121.6, 114.5, 35.5, 34.2.

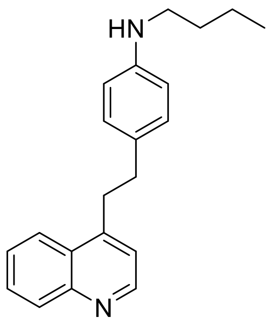

N-butyl-4-(2-(quinolin-4-yl)ethyl)aniline (**30**)

Compound **29** (100 mg, 0.405 mmol) and butyraldehyde (0.38 g, 0.448 mmol) were dissolved in THF (5 mL) under inert conditions. NaBH(OAc)$_3$ (141 mg, 0.672 mmol) was added and the reaction was stirred at rt for 2 h. The reaction was quenched with saturated $NaHCO_3$ and extracted with EtOAc. The organic phase was washed with saturated $NaHCO_3$ and brine, dried over Na_2SO_4, filtered and concentrated under reduced pressure. The resultant oil was purified by flash chromatography on a silica gel column (EtOAc:hexane; 40:60 top 100:0) to afford **30** as white crystals (75 mg, 30%). ^{1}H NMR (DMSO-d_6) δ (ppm): 8.76 (d, 1H, *J* = 4.4 Hz), 8.20 (d, 1H, *J* = 7.9 Hz), 8.02 (d, 1H, *J* = 7.8 Hz), 7.82 – 7.71 (m, 1H), 7.63 (ddd, 1H, *J* = 8.2, 6.9, 1.2 Hz), 7.34 (d, 1H, *J* = 4.4 Hz), 6.97 (d, 2H, *J* = 8.4 Hz), 6.51 (dd, 2H, *J* = 31.7, 8.3 Hz), 5.33 (s, 1H), 3.31 – 3.26 (m, 2H), 2.95 (d, 2H, *J* = 3.2 Hz), 2.89 – 2.81 (m, 2H), 1.51 (dt, 2H, *J* = 14.7, 7.2 Hz), 1.37 (dq, 2H, *J* = 14.4, 7.3 Hz), 0.91 (t, 3H, *J* = 7.3 Hz).

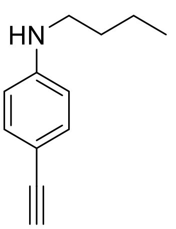

N-butyl-4-ethynylaniline (**31**)

4-ethynylaniline (250 mg, 2.14 mmol) and butyraldehyde (192 mg, 2.14 mmol) were dissolved in THF (2 mL) under inert conditions and the reaction was stirred at rt for 3 h. $NaBH(OAc)_3$ (901 mg, 4.27 mmol) was added and the reaction was stirred at rt for 1 h. The reaction was quenched with saturated $NaHCO_3$ and extracted with EtOAc. The organic phase was washed with saturated $NaHCO_3$ and brine, dried over Na_2SO_4, filtered and concentrated under reduced pressure. The resultant oil was purified by flash chromatography on a silica gel column (EtOAc:hexane; 0:100 to 10:90) to afford **31** as a yellow oil (150 mg, 41%). ^{1}H NMR (DMSO-d_6) δ (ppm): 7.16 (d, 2H, *J* = 8.7 Hz), 6.51 (d, 2H, *J* = 8.8 Hz), 3.79 (s, 1H), 3.00 (dd, 2H, *J* = 12.5, 7.0 Hz), 1.52 (m, 2H), 1.37 (dq, 2H, *J* = 14.1, 7.1 Hz), 0.91 (t, 3H, *J* = 7.3 Hz).

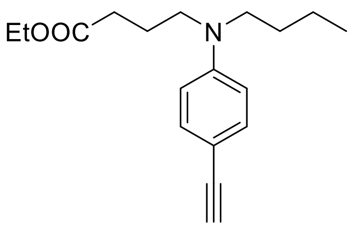

Ethyl 4-(butyl(4-ethynyl)phenyl)amino)butanoate (**32**)

Compound **31** (55 mg, 0.32 mmol) and ethyl 4-bromobutanoate (227 mg, 1.6 mmol) were dissolved in DMF (0.5 mL). K_2CO_3 (130 mg, 0.95 mmol) was added and the reaction was stirred under microwave irradiation at 90 ^oC for 8 h. The mixture was diluted with EtOAc and the organic phase was washed with saturated $NaHCO_3$ and brine, dried over Na_2SO_4, filtered and concentrated under reduced pressure. The residue was purified by flash chromatography on a silica gel column (EtOAc:hexane; 0:100 to 15:85) to afford **32** as a yellow oil (25 mg, 28%). ^{1}H NMR (500 MHz, DMSO) δ 7.24 (d, 2H, *J* = 8.9 Hz), 6.64 (d, 2H, *J* = 9.0 Hz), 4.07 (q, 2H, *J* = 7.1 Hz), 3.32 – 3.23 (m, 4H), 2.40 – 2.33 (m, 2H), 1.79 – 1.70 (m, 2H), 1.48 (dt, 2H, *J* = 15.0, 7.5 Hz), 1.36 – 1.27 (m, 2H), 1.19 (t, 3H, *J* = 7.1 Hz), 0.92 (t, 3h, *J* = 7.3 Hz).

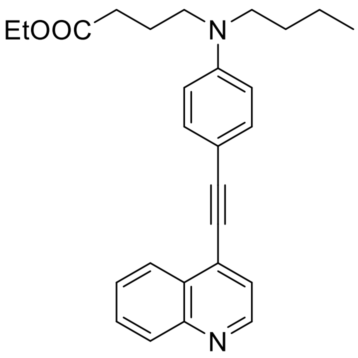

Ethyl 4-(butyl(4-(quinolin-4-ylethynyl)phenyl)amino)butanoate (**33**)

Compound **32** (8 mg, 0.03 mmol), 4-bromoquinoline (6.5 mg, 0.031 mmol) and triethylamine (7.8 µL, 0.06 mmol) were dissolved in DMF (0.4 mL) under inert conditions. CuI (1.8 mg, 0.005 mmol) and $PdCl_2(PPh_3)_2$ (2 mg, 0.003 mmol) were added and the reaction was stirred under microwave irradiation at 60 ^oC for 1.5 h. The mixture was diluted with EtOAc and the organic phase was washed with saturated $NaHCO_3$ and brine, dried over Na_2SO_4, filtered and concentrated under reduced pressure. The residue was purified by flash chromatography on a silica gel column (EtOAc:hexane; 25:75 to 55:45) to afford **33** (9.3 mg, 80%). ^1H NMR (DMSO-d_6) δ (ppm): 8.87 (d, 1H, *J* = 4.5 Hz), 8.39 – 8.33 (m, 1H), 8.06 (d, 1H, *J* = 8.3 Hz), 7.86 – 7.81 (t, 1H), 7.75 – 7.71 (t, 1H), 7.62 (d, 1H, *J* = 4.5 Hz), 7.53 (d, 2H, *J* = 8.9 Hz), 6.77 (d, 2H, *J* = 9.0 Hz), 4.08 (q, 2H, *J* = 7.1 Hz), 3.39 – 3.34 (m, 4H), 2.38 (t, 2H, *J* = 6.7 Hz), 1.84 – 1.74 (m, 2H), 1.57 – 1.48 (m, 2H), 1.38 – 1.29 (m, 2H), 1.19 (t, 3H, *J* = 7.1 Hz), 0.93 (t, 3H, *J* = 7.4 Hz).

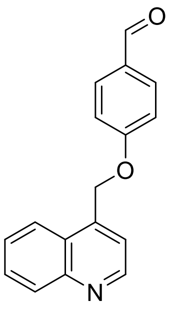

4-(quinolin-4-ylmethoxy)benzaldehyde (**34**)

4-hydroxybenzaldehyde (80.5 mg, 0.66 mmol), 4-(bromomethyl)quinoline (50 mg, 0.22 mmol) and K_2CO_3 (120 mg, 0.88 mmol) were dissolved in THF (2 mL) and H2O (150 µL). KI (73 mg, 0.44 mmol) was added and the reaction was stirred at rt for 48 h. The mixture was diluted with EtOAc and the organic phase was washed with saturated $NaHCO_3$ and brine, dried over

Na_2SO_4, filtered and concentrated under reduced pressure. The residue was purified by flash chromatography on a silica gel column (EtOAc:hexane; 35:65 to 65:35) to afford **34** as a white powder (23.2 mg, 40%). ^{1}H NMR (DMSO-d$_6$) δ (ppm): 9.92 (s, 1H), 8.95 (d, J = 4.3 Hz, 1H), 8.21 (d, J = 8.1 Hz, 1H), 8.11 (d, J = 8.3 Hz, 1H), 7.94 (d, J = 8.7 Hz, 2H), 7.84 (dd, J = 11.2, 4.1 Hz, 1H), 7.71 (t, J = 8.0 Hz, 1H), 7.69 (d, J = 4.3 Hz, 1H), 7.37 (d, J = 8.7 Hz, 2H), 5.84 (s, 2H).^{13}C NMR (DMSO-d$_6$) δ (ppm): 192.26, 163.83 , 151.3 , 148.51, 142.54, 132.74, 131.05, 130.54, 130.47, 127.79, 126.34, 124.84, 120.31, 116.36, 67.47

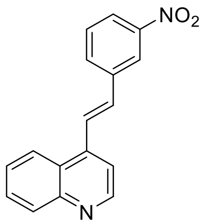

(E)-4-(3-nitrostyryl)quinoline (**35**)

Lepidine (143 mg, 1.0 mmol) and 3-nitrobenzaldehyde (166 mg, 1.1 mmol) were dissolved in NMP (0.47 mL). $ZnCl_2$ (136 mg, 1.0 mmol) was added and the reaction was stirred under microwave irradiation at 150 °C for 20 min. The mixture was diluted with a solution of NH_3-H_2O/NH_4Cl/H_2O (1:1:10, v/v/v) and extracted with CH_2Cl_2. The organic phase was washed with a solution of saturated NH_4Cl/NH_3-H_2O (10:1, v/v), saturated $NaHCO_{3(aq)}$ and then concentrated under reduced pressure. The residue was purified by flash chromatography (hexane:EtOAc; 90:10 to 10:90) then triturated in MTBE/hexanes (1:1) to afford **35** as a yellow solid (92 mg, 34%). MS (ESI+): m/z = 277.

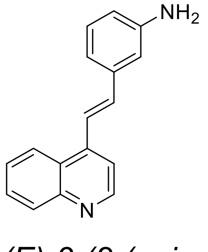

(E)-3-(2-(quinolin-4-yl)vinyl)aniline (**36**)

Compound **35** (22 mg, 0.08 mmol) and $SnCl_2 \cdot 2H_2O$ (230 mg, 0.93 mmol) were suspended in EtOH (1 mL) and the reactions was stirred at reflux for 14 h. The solvent was removed under reduced pressure. The residue was dissolved in 0.1M HCl and purified by reverse phase

chromatography (H_2O, MeOH) to afford **36** as an amorphous solid (8.9 mg, 45%). ^{1}H NMR (MeOD-d_4) δ (ppm): 8.89 (d, 1H, *J* = 5.6 Hz), 8.60 (dd, 1H, *J* = 8.7, 1.2 Hz), 8.19 – 8.08 (m, 2H), 8.07 – 7.96 (m, 2H), 7.86 (ddd, 1H, *J* = 8.4, 6.9, 1.3 Hz), 7.72 (d, 1H, *J* = 16.0 Hz), 7.36 – 7.23 (m, 3H), 6.95 (dt, 1H, *J* = 6.2, 2.4 Hz). ^{13}C NMR (MeOD-d_4) δ (ppm): 152.4, 145.9, 145.3, 142.4, 142.2, 138.2, 134.5, 131.0, 130.0, 127.7, 126.0, 124.2, 122.0, 121.6, 120.3, 117.8, 117.3, 49.5, 49.5, 49.3, 49.3, 49.2, 49.0, 48.8, 48.7, 48.5.

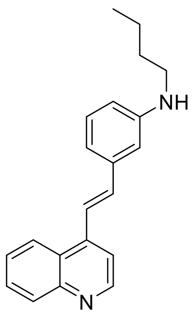

(E)-N-butyl-3-(2-(quinolin-4-yl)vinyl)aniline (**37**)

Compound **36** (20 mg, 0.081 mmol) and $NaBH(OAc)_3$ (20.7 mg, 0.097 mmol) were dissolved in CH_2Cl_2 (0.8 mL). AcOH (10 μL, 0.162 mmol) and butyraldehyde (5.6 mg, 0.077 mmol) were added and the reaction was stirred at rt for 1 h. The reaction was quenched with 1M HCl and the organic phase was separated. The aqueous phase was extracted with CH_2Cl_2, and the combined organic phases were concentrated under reduced pressure. The residue was purified by flash chromatography on a silica gel column (hexane:EtOAc; 90:10 to 10:90)) to afford **37** as a yellow oil (9.8 mg, 40%). ^{1}H NMR ($CDCl_3$) δ (ppm): 8.89 (d, 1H, *J* = 4.6 Hz), 8.22 (dd, 1H, *J* = 8.5, 0.9 Hz), 8.15 (dd, 1H, *J* = 8.4, 0.6 Hz), 7.74 (ddd, 2H, *J* = 12.3, 8.8, 8.3 Hz), 7.59 (ddd, 2H, *J* = 8.4, 6.8, 1.7 Hz), 7.28 (d, 1H, *J* = 16.0 Hz), 7.23 (t, 1H, *J* = 7.8 Hz), 6.98 (d, 1H, *J* = 7.6 Hz), 6.86 – 6.80 (m, 1H), 6.62 (ddd, 1H, *J* = 8.1, 2.3, 0.7 Hz), 3.26 – 3.13 (m, 2H), 1.65 (ddd, 2H, *J* = 14.6, 8.3, 6.5 Hz), 1.47 (dq, 2H, *J* = 14.7, 7.4 Hz), 0.99 (t, 3H, *J* = 7.4 Hz). ^{13}C NMR ($CDCl_3$) δ (ppm): 150.1, 149.1, 148.5, 143.6, 137.6, 136.2, 130.0, 129.8, 129.5, 126.6, 126.6, 123.7, 122.5, 117.2, 116.3, 113.6, 111.2, 43.8, 31.8, 20.5, 14.1.

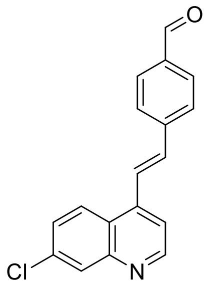

(E)-4-(2-(7-chloroquinolin-4-yl)vinyl)benzaldehyde (**38**)

7-chloro-4-methylquinoline (150 mg, 0.85 mmol), terephthalaldehyde (340 mg, 2.54 mmol) and $ZnCl_2$ (51.8 mg, 0.381 mmol) were combined and stirred under microwave irradiation at 150 °C for 20 min. The mixture was then dissolved in DMF, diluted with EtOAc and the organic phase was washed with saturated $NaHCO_3$ and brine, dried over Na_2SO_4, filtered and concentrated under reduced pressure. The residue was purified by flash chromatography on a silica gel column (EtOAc/hexane; 20:80 to 100:0) to afford **38** as yellow crystals (150 mg, 60%). ^{1}H NMR (DMSO-d_6) δ (ppm): 10.04 (s, 1H), 8.96 (d, 1H, *J* = 4.7 Hz), 8.64 (d, 1H, *J* = 9.1 Hz), 8.30 (d, 1H, *J* = 16.2 Hz), 8.11 (d, 1H, *J* = 2.2 Hz), 8.08 (d, 2H, *J* = 8.3 Hz), 7.98 (d, 2H, *J* = 8.3 Hz), 7.95 (d, 1H, *J* = 4.7 Hz), 7.73 (d, 1H, *J* = 16.1 Hz), 7.71 (dd, 1H, *J* = 9.0, 2.3 Hz).

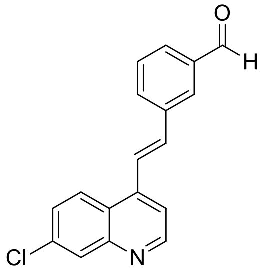

(E)-3-(2-(7-chloroquinolin-4-yl)vinyl)benzaldehyde (**39**)

7-Chloro-4-methylquinoline (4.01 g, 22.6 mmol, 1 eq.), isophthaldehyde (5.75 g, 42.9 mmol, 1.9 eq.) and zinc chloride (1.38 g, 10.15 mmol, 0.45 eq.) were added neat to a 10-20 mL microwave vessel. The compounds were heated in the sealed microwave vessel until dissolution. The dark brown reaction mixture was heated under microwave irradiation at 150 °C for 20 min. The obtained solid product was dissolved in DMF and poured in EtOAc. This solution was washed twice with brine and saturated aqueous $NaHCO_3$ solution (1:1) and one time with brine to remove the zinc chloride salt. The organic phase was dried over

Na_2SO_4 and concentrated under reduced pressure to give a yellowish residue. The crude product was purified by flash chromatography on 120 g silica gel column (hexane/EtOAc 80:20 to 0:10 (v/v) in 30 min) to afford **39** as a yellowish amorphous solid (5.35 g, 81%). MS (ESI+): m/z = 294 $[M+H]^+$; ^1H NMR ($CDCl_3$) δ (ppm): 10.08 (s, 1H), 8.90 (d, 1H, *J* = 4.5 Hz), 8.15-8.13 (m, 3H), 7.86-7.81 (m, 3H), 7.62-7.58 (m, 2H), 7.54 (dd, 1H, *J* = 2.0 and 9.0 Hz), 7.36 (d, 1H, *J* = 16 Hz). ^{13}C NMR ($CDCl_3$) δ (ppm): 192.2, 151.4, 149.3, 142.9, 137.5, 137.2, 135.7, 134.5, 133.2, 130.6, 129.9, 129.2, 127.9, 127.7, 125.1, 124.9, 124.5, 117.6.

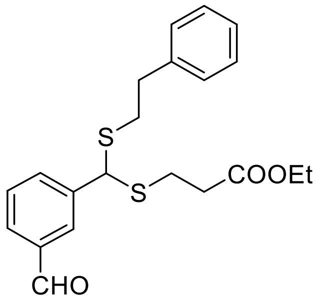

Ethyl 3-(((3-formylphenyl)(phenethylthio)methyl)thio)propanoate (**41**)

Isophthalaldehyde (500 mg, 3.73 mmol) and TsOH.H_2O (212 mg, 2.24 mmol) were dissolved in toluene (15 mL) and 4Å molecular sieves were added. 2-phenylethane-1-thiol (1.19 mL, 8.95 mmol) and ethyl 3-mercaptopropanoate (1.13 mL, 8.95 mmol) were added and the reaction mixture was stirred at reflux for 1.5 h under inert conditions. The mixture was diluted with EtOAc and the organic phase was washed with saturated $NaHCO_{3(aq)}$ and brine, dried over Na_2SO_4, filtered and concentrated under reduced pressure. The residue was purified by flash chromatography on a silica gel column (EtOAc:hexanes; 0:100 to 20:80) to afford **41** as an amorphous solid. ^1H NMR (DMSO-d_6) δ (ppm): 10.02 (s, 1H), 7.96 (s, 1H), 7.85 (d, 1H, *J* = 7.6 Hz), 7.76 (d, 1H, *J* = 7.8 Hz), 7.61 (t, 1H, *J* = 7.7 Hz), 7.27 (t, 2H, *J* = 7.4 Hz), 7.19 (t, 1H, *J* = 8.6 Hz), 7.18 (d, 2H, *J* = 7.5 Hz), 5.37 (s, 1H), 4.04 (q, 2H, *J* = 7.1 Hz), 2.87 – 2.79 (m, 3H), 2.79 – 2.71 (m, 2H), 2.65 (dt, 1H, *J* = 18.5, 6.3 Hz), 2.60 (dd, 2H, *J* = 11.9, 6.8 Hz), 1.15 (t, 3H, *J* = 7.1 Hz).

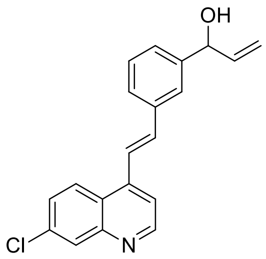

(E)-1-(3-(2-(7-chloroquinolin-4-yl)vinyl)phenyl)prop-2-en-1-ol (**42**)

A suspension of the aldehyde **39** (3.00 g, 10.20 mmol, 1 eq.) in toluene (24 mL) at 0 °C was degassed by purging three times with vacuum and nitrogen. Vinylmagnesium bromide (1.0 M in THF, 10.8 mL, 10.8 mmol, 1.2 eq.) was added dropwise over 20 min while the internal temperature was maintained at < 10 °C. The brown reaction mixture was stirred at 0–5 °C for 2.5 h and quenched by slowly adding 10% aqueous ammonium acetate (25 mL). This two-phase mixture was stirred for 1 h to ensure the solvolysis of the magnesium salts. The separated organic layer was washed with water (2 X 25 mL), dried over Na_2SO_4 and concentrated in vacuo to give an orange residue. The crude product was purified by flash chromatography on 80 g silica gel column (hexane:EtOAc; 10:0 to 50:50) to afford the **42** as a colorless oil (1.71 g, 52%). MS (ESI+): m/z = 322 $[M+H]^+$. ^{1}H NMR ($CDCl_3$) δ (ppm): 8.87 (d, 1H, *J* = 4.5 Hz), 8.16-8.12 (m, 2H), 7.74 (d, 1H, *J* = 16.0 Hz), 7.67 (s, 1H), 7.58 (d, 1H, *J* = 4.5 Hz), 7.56-7.53 (m, 2H), 7.46-7.40 (m, 2H), 7.35 (d, 1H, *J* = 16.0 Hz), 6.17-6.10 (m, 1 H), 5.45 (td, 1H, *J* = 1.5 and 17.0 Hz), 5.33 (d, 1H, *J* = 6.0 Hz), 5.29 (td, 1H, *J* = 1.5 and 10.0 Hz). ^{13}C NMR ($CDCl_3$) δ (ppm): 151.1, 149.0, 147.5, 143.4, 143.2, 140.1, 136.6, 135.7, 135.4, 129.2, 128.9, 127.5, 127.1, 126.6, 125.0, 125.0, 122.5, 117.2, 115.6, 75.2.

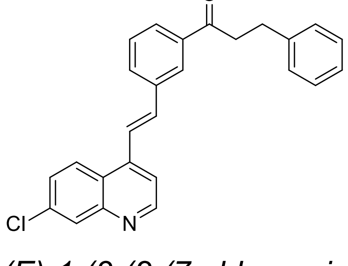

(E)-1-(3-(2-(7-chloroquinolin-4-yl)vinyl)phenyl)-3-phenylpropan-1-one (**43**)

Obtained according to general procedure A from **42** and 4-iodobenzene. Yellow oil (0.270 g, 73%). MS (ESI+): m/z = 398 $[M+H]^+$; ^{1}H NMR ($CDCl_3$) δ (ppm): 8.92 (d, 1H, *J* = 4.5 Hz), 8.22 (s, 1H), 8.17-8.15 (m, 2H), 7.95 (d, 1H, *J* = 7.5 Hz), 7.83-7.80 (m, 2H), 7.60-7.53 (m, 3H),

7.39-7.25 (m, 6H), 3.39 (t, 2H, *J* = 7.8 Hz), 3.14 (t, 2H, *J* = 7.8 Hz). ^{13}C NMR ($CDCl_3$) δ (ppm): 198.9, 151.2, 149.1, 142.7, 141.1, 137.5, 136.8, 135.4, 134.7, 131.5, 129.2, 129.0, 128.6, 128.5, 128.4, 127.6, 126.5, 126.3, 124.9, 124.7, 123.7, 117.3, 40.7, 30.1.

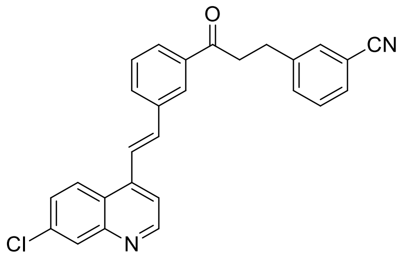

(E)-3-(3-(3-(2-(7-chloroquinolin-4-yl)vinyl)phenyl)-3-oxopropyl)benzonitrile (**44**)

Obtained according to general procedure A from **42** and 3-iodobenzonitrile. Colorless oil (0.079 g, 69%). MS (ESI+): m/z = 423 $[M+H]^+$; ^{1}H NMR ($CDCl_3$) δ (ppm): 8.91 (d, 1H, *J* = 4.0 Hz), 8.21 (s, 1H), 8.17-8.15 (m, 2H), 7.95 (d, 1H, *J* = 7.5 Hz), 7.84-7.80 (m, 2H), 7.60-7.52 (m, 6H), 7.43 (t, 1H, *J* = 8.0 Hz), 7.37 (d, 1H, *J* = 16.0 Hz), 3.40 (t, 2H, *J* = 7.5 Hz), 3.17 (t, 2H, *J* = 7.5 Hz). ^{13}C NMR ($CDCl_3$) δ (ppm): 198.0, 151.2, 149.1, 142.6, 142.6, 137.2, 137.0, 135.4, 134.6, 133.2, 132.0, 131.6, 130.0, 129.3, 129.0, 128.3, 127.6, 126.5, 124.9, 124.7, 123.9, 118.9, 117.4, 112.6, 39.8, 29.4.

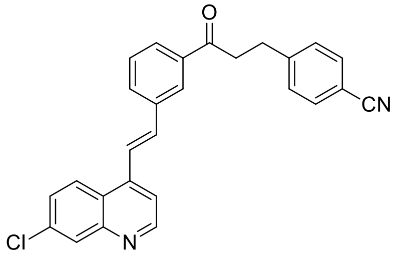

(*E*)-4-(3-(3-(2-(7-chloroquinolin-4-yl)vinyl)phenyl)-3-oxopropyl)benzonitrile (**45**)

Obtained according to general procedure A from **42** and 4-iodobenzonitrile. White solid (0.347 g, 59%). MS (ESI+): m/z = 423 $[M+H]^+$; ^{1}H NMR ($CDCl_3$) δ (ppm): 8.92 (d, 1H, *J* = 4.5 Hz), 8.21 (s, 1H), 8.17-8.15 (m, 2H), 7.94 (d, 1H, *J* = 7.5 Hz), 7.85-7.82 (m, 2H), 7.63-7.55 (m, 5H), 7.42 (d, 2H, *J* = 7.0 Hz), 7.38 (d, 1H, *J* = 16.0 Hz), 3.41 (t, 2H, *J* = 7.5 Hz), 3.20 (t, 2H, *J* = 7.5 Hz). ^{13}C NMR ($CDCl_3$) δ (ppm): 197.9, 151.2, 149.1, 146.8, 142.7, 137.2, 137.0, 135.5, 134.6, 132.4, 131.7, 129.4, 129.3, 129.0, 128.3, 127.7, 126.5, 124.9, 124.7, 124.0, 118.9, 117.4, 110.2, 39.6, 29.9.

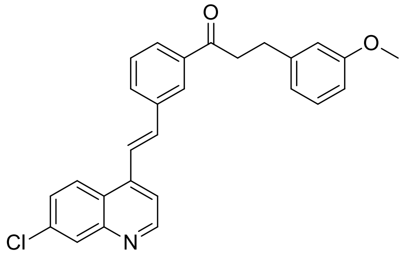

(E)-1-(3-(2-(7-chloroquinolin-4-yl)vinyl)phenyl)-3-(3-methoxyphenyl)propan-1-one (**46**)

Obtained according to general procedure A from **42** and 3-iodoanisole. Colorless oil (0.090 g, 85%). MS (ESI+): m/z = 428 $[M+H]^+$; ^1H NMR ($CDCl_3$) δ (ppm): 8.92 (d, 1H, *J* = 4.5 Hz), 8.22 (s, 1H), 8.18-8.15 (m, 2H), 7.95 (d, 1H, *J* = 8.0 Hz), 7.84-7.81 (m, 2H), 7.61-7.55 (m, 3H), 7.37 (d, 1H, *J* = 16.0 Hz), 7.26 (t, 1H, *J* = 8.0 Hz), 6.89 (d, 1H, *J* = 8.0 Hz), 6.87-6.86 (m, 1 H), 6.80 (dd, 1H, *J* = 2.5 and 8.0 Hz), 3.83 (s, 3H), 3.39 (t, 2H, *J* = 7.5 Hz), 3.11 (t, 2H, *J* = 7.5 Hz). ^{13}C NMR ($CDCl_3$) δ (ppm): 198.9, 159.8, 151.2, 149.2, 142.8, 142.7, 137.5, 136.9, 135.4, 134.7, 131.5, 129.6, 129.2, 129.0, 128.4, 127.6, 126.5, 124.9, 124.7, 123.8, 120.8, 117.4, 114.4, 111.4, 55.2, 40.6, 30.2.

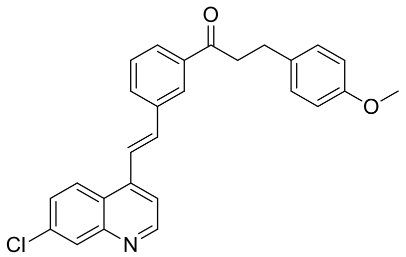

(E)-1-(3-(2-(7-chloroquinolin-4-yl)vinyl)phenyl)-3-(4-methoxyphenyl)propan-1-one (**47**)

Obtained according to general procedure A from **42** and 4-iodoanisole. White solid (0.501 g, 47%). MS (ESI+): m/z = 428 $[M+H]^+$; ^1H NMR ($CDCl_3$) δ (ppm): 8.90 (d, 1H, *J* = 4.0 Hz), 8.20 (s, 1H), 8.16-8.13 (m, 2H), 7.94 (d, 1H, *J* = 7.6 Hz), 7.82-7.78 (m, 2H), 7.59-7.51 (m, 3H), 7.36 (d, 1H, *J* = 16.4 Hz), 7.22 (d, 2H, *J* = 8.2 Hz), 6.87 (d, 2H, *J* = 8.2 Hz), 3.81 (s, 3H), 3.35 (t, 2H, *J* = 7.6 Hz), 3.07 (t, 2H, *J* = 7.6 Hz). ^{13}C NMR ($CDCl_3$) δ (ppm): 199.1, 158.1, 151.2, 149.1, 142.7, 137.5, 136.8, 135.4, 134.7, 133.1, 131.4, 129.4, 129.4, 129.2, 129.0, 128.4, 127.6, 126.5, 124.9, 123.7, 117.3, 114.0, 55.3, 40.9, 29.3.

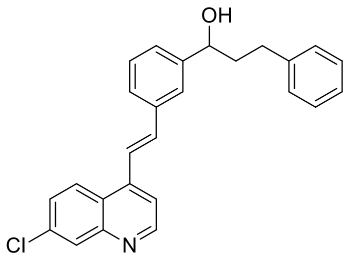

(E)-1-(3-(2-(7-chloroquinolin-4-yl)vinyl)phenyl)-3-phenylpropan-1-ol (**48**)

Obtained from **43** according to general procedure D with no purification step. Yellow oil (0.121 g, 81%). MS (ESI+): m/z = 400 $[M+H]^+$; ^{1}H NMR ($CDCl_3$) δ (ppm): 8.90 (d, 1H, *J* = 4.5 Hz), 8.19-8.15 (m, 2H), 7.77 (d, 1H, *J* = 16.5 Hz), 7.65 (s, 1H), 7.61 (d, 1H, *J* = 4.5 Hz), 7.56-7.55 (m, 2H), 7.44 (t, 1H, *J* = 4.5 Hz), 7.38-7.22 (m, 7H + $CDCl_3$), 4.80 (dd, 1H, *J* = 5.5 and 8.0 Hz), 2.84-2.77 (m, 2H), 2.26-2.19 (m, 1H), 2.08-2.15 (m, 1H).

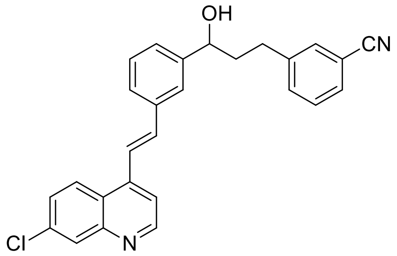

(E)-3-(3-(3-(2-(7-chloroquinolin-4-yl)vinyl)phenyl)-3-hydroxypropyl)benzonitrile (**49**)

Obtained from **44** according to general procedure D with no purification step. Yellow oil (0.021 g, quantitative). MS (ESI+): m/z = 425 $[M+H]^+$; ^{1}H NMR ($CDCl_3$) δ (ppm): 8.87 (br s, 1H), 8.17-8.12 (m, 2H), 7.75 (d, 1H, *J* = 16.0 Hz), 7.62-7.59 (m, 2H), 7.56-7.33 (m, 9 H), 4.78 (dd, 1H, J = 5.5 and 8.0 Hz), 2.89-2.80 (m, 2H), 2.24-2.16 (m, 1H), 2.12-2.05 (m, 1H). ^{13}C NMR ($CDCl_3$) δ (ppm): 151.0, 148.9, 145.2, 143.2, 136.6, 135.6, 135.4, 133.1, 132.0, 129.8, 129.2, 129.2, 128.8, 127.6, 126.7, 126.6, 125.0, 124.5, 122.6, 119.0, 117.2, 112.4, 73.3, 40.2, 31.6.

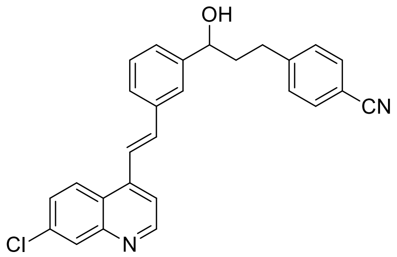

(E)-4-(3-(3-(2-(7-chloroquinolin-4-yl)vinyl)phenyl)-3-hydroxypropyl)benzonitrile (**50**)

Obtained according to general procedure D from **45**. The crude product was purified by flash chromatography on a silica gel column (hexanes:EtOAc; 90:10 to 30:70) to afford **50** as a yellow oil (0.307 g, 88%). MS (ESI+): m/z = 425 $[M+H]^+$; ^{1}H NMR ($CDCl_3$) δ (ppm): 8.84 (d, 1H, *J* = 3.6 Hz), 8.13 (d, 1H, *J* = 9.2 Hz), 8.09 (d, 1H, *J* = 2.0 Hz), 7.73 (d, 1H, *J* = 16.0 Hz), 7.60-7.53 (m, 6H), 7.44 (t, 1H, *J* = 7.6 Hz), 7.37-7.30 (m, 4H), 4.79 (dd, 1H, *J* = 5.6 and 8.0 Hz), 2.94-2.79 (m, 2H), 2.26-2.19 (m, 1H), 2.10-2.06 (m, 1H). ^{13}C NMR ($CDCl_3$) δ (ppm): 151.0, 149.0, 147.5, 145.3, 143.1, 136.6, 135.6, 135.4, 132.2, 129.3, 129.2, 129.2, 128.8, 127.5, 126.6, 126.6, 124.9, 124.5, 122.5, 119.0, 117.2, 109.8, 73.3, 40.0, 32.3.

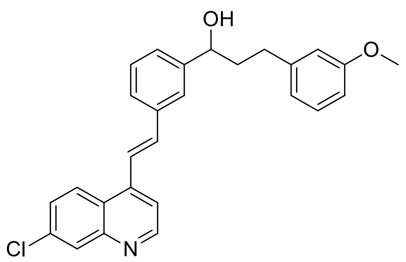

(E)-1-(3-(2-(7-chloroquinolin-4-yl)vinyl)phenyl)-3-(3-methoxyphenyl)propan-1-ol (**51**)

Obtained from **46** according to general procedure D with no purification step. Colorless oil (0.006 g, 60%). MS (ESI+): m/z = 430 $[M+H]^+$; ^{1}H NMR ($CDCl_3$) δ (ppm): 8.89 (d, 1H, *J* = 4.8 Hz), 8.17 (d, 1H, *J* = 8.8 Hz), 8.13 (d, 1H, *J* = 2.0 Hz), 7.76 (d, 1H, *J* = 16.4 Hz), 7.60 (s, 1H), 7.59-7.46 (m, 3H), 7.44 (t, 1H, *J* = 8.0 Hz), 7.39-7.18 (m, 3H), 6.86-6.76 (m, 3H), 4.80-4.77 (dd, 1H, J = 5.6 and 7.2 Hz), 3.82 (s, 3H), 2.86-2.67 (m, 2H), 2.27-2.17 (m, 1H), 2.15-2.13 (m, 1H).

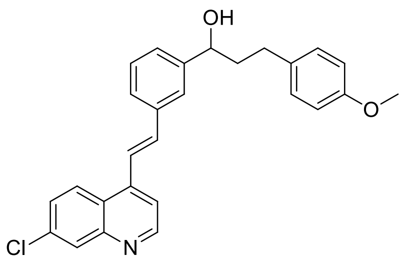

(E)-1-(3-(2-(7-chloroquinolin-4-yl)vinyl)phenyl)-3-(4-methoxyphenyl)propan-1-ol (**52**)

Obtained according to general procedure D from **47**. The crude product was purified by flash chromatography on a silica gel column (hexanes:EtOAc; 90:10 to 50:50) to afford **52** as a colorless oil (0.180 g, 75%).. MS (ESI+): m/z = 430 $[M+H]^+$; ^{1}H NMR ($CDCl_3$) δ (ppm): 8.86 (s,

1H), 8.15 (d, 1H, *J* = 9.2 Hz), 8.11 (d, 1H, *J* = 2.0 Hz), 7.74 (d, 1H, *J* = 16.0 Hz), 7.63 (s, 1H), 7.59-7.52 (m, 3H), 7.43 (t, 1H, *J* = 7.6 Hz), 7.37 (d, 1H, *J* = 7.6 Hz), 7.34 (d, 1H, *J* = 16.0 Hz), 7.16 (d, 2H, *J* = 8.4 Hz), 6.87 (d, 2H, *J* = 8.4 Hz), 4.80-4.77 (m, 1H), 3.81 (s, 3H), 2.78-2.69 (m, 2H), 2.24-2.14 (m, 1H), 2.12-2.03 (m, 1H). ^{13}C NMR ($CDCl_3$) δ (ppm): 157.9, 151.1, 149.0, 145.7, 143.2, 136.4, 135.7, 135.3, 133.6, 129.3, 129.3, 129.1, 128.8, 127.5, 126.7, 126.5, 125.0, 124.5, 122.3, 117.2, 113.9, 73.5, 55.3, 40.8, 31.2.

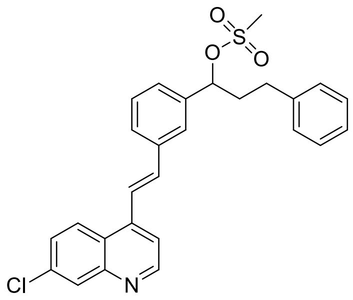

(E)-1-(3-(2-(7-chloroquinolin-4-yl)vinyl)phenyl)-3-phenylpropyl methanesulfonate (**53**)

Obtained according to general procedure E from **48**. Yellow oil (0.096 g, quantitative). MS (ESI+): m/z = 478 $[M+H]^+$.

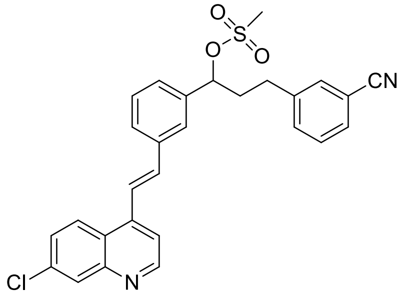

(E)-1-(3-(2-(7-chloroquinolin-4-yl)vinyl)phenyl)-3-(3-cyanophenyl)propyl methanesulfonate (**54**)

Obtained according to general procedure E from **49**. Yellow oil (0.482 g, quantitative). MS (ESI+): m/z = 503 $[M+H]^+$.

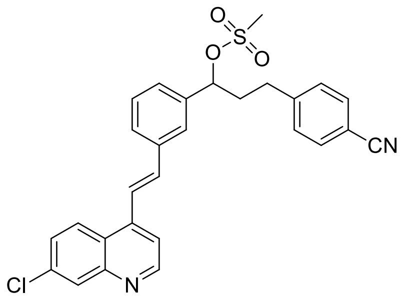

(E)-1-(3-(2-(7-chloroquinolin-4-yl)vinyl)phenyl)-3-(4-cyanophenyl)propyl methanesulfonate (**55**)

Obtained according to general procedure E from **50**. Yellow oil (0.494 g, quantitative). MS (ESI+): m/z = 503 $[M+H]^+$.

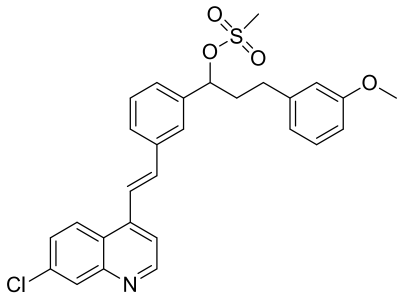

(E)-1-(3-(2-(7-chloroquinolin-4-yl)vinyl)phenyl)-3-(3-methoxyphenyl)propyl methanesulfonate (**56**)

Obtained according to general procedure E from **51**. Yellow oil (0.496 g, quantitative). MS (ESI+): m/z = 508 $[M+H]^+$.

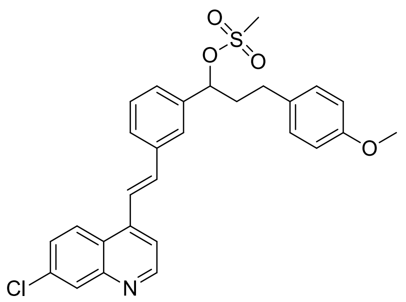

(E)-1-(3-(2-(7-chloroquinolin-4-yl)vinyl)phenyl)-3-(4-methoxyphenyl)propyl methanesulfonate (**57**)

Obtained according to general procedure E from **52**. Yellow oil (0.370 g, quantitative). MS (ESI+): m/z = 508 $[M+H]^+$.

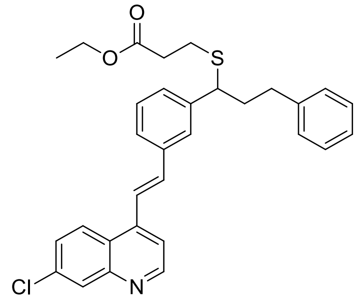

(E)-ethyl 3-((1-(3-(2-(7-chloroquinolin-4-yl)vinyl)phenyl)-3-phenylpropyl)thio)propanoate (**58**)

Obtained according to general procedure F from **53**. Colorless oil (0.491 g, 47%). A fraction was collected and the two enantiomers of **58** were separated on chiral column (Chiralpak IA 10 x 250mm, 5µm) with Hexanes (0.1% Et_2NH)/iPrOH (0.1% Et_2NH) 80:20 to obtain **(+)-58** (0.177 g, ee = 100%) and **(-)-58** (0.171 g, ee = 100%) as colorless oils. MS (ESI+): m/z = 516 $[M+H]^+$; ^1H NMR ($CDCl_3$) δ (ppm): 8.93 (d, 1H, *J* = 4.4 Hz), 8.22 (d, 1H, *J* = 9.2 Hz), 8.17 (d, 1H, *J* = 2.2 Hz), 7.79 (d, 1H, *J* = 16.0 Hz), 7.63 (d, 1H, *J* = 4.4 Hz), 7.60-7.55 (m, 3H), 7.44 (t, 1H, *J* = 7.8 Hz), 7.39-7.18 (m, 7H + $CDCl_3$), 4.14 (q, 2H, *J* = 7.2 Hz), 3.86 (t, 1H, *J* = 8.0 Hz), 2.70 (t, 2H, *J* = 7.6 Hz), 2.62 (t, 2H, *J* = 7.5 Hz), 2.62 (t, 2H, *J* = 8.0 Hz), 2.76 (quintuplet, 1H, *J* = 7.5 Hz), 2.22 (quintuplet, 1H, *J* = 7.5 Hz), 1.26 (t, 3H, *J* = 7.2 Hz). ^{13}C NMR ($CDCl_3$) δ (ppm): 171.9, 151.2, 149.1, 143.3, 143.1, 141.1, 136.7, 135.7, 135.3, 129.2, 129.0, 128.6, 128.5, 128.4, 127.5, 126.7, 126.1, 126.1, 125.1, 122.6, 117.2, 60.7, 49.1, 37.9, 34.6, 33.6, 26.1, 14.2.

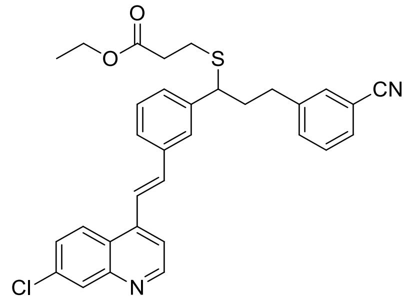

(E)-ethyl 3-((1-(3-(2-(7-chloroquinolin-4-yl)vinyl)phenyl)-3-(3-cyanophenyl)propyl)thio)propanoate (**59**)

Obtained according to general procedure F from **54**. Colorless oil (0.219 g, 48%). MS (ESI+): m/z = 541 $[M+H]^+$; ^1H NMR ($CDCl_3$) δ (ppm): 8.93 (d, 1H, *J* = 4.6 Hz), 8.21 (d, 1H, *J* = 9.0 Hz), 8.17 (d, 1H, *J* = 2.1 Hz), 7.79 (d, 1H, *J* = 16.1 Hz), 7.63 (d, 1H, *J* = 4.6 Hz), 7.60-7.55 (m, 3H),

7.54-7.50 (m, 1H), 7.48-7.43 (m, 2H), 7.41 (m, 3H), 7.35-7.31 (m, 1H), 4.15 (q, 2H, *J* = 7.1 Hz), 3.84 (t, 1H, *J* = 7.5 Hz), 2.83-2.66 (m, 2H), 2.63 (t, 2H, J = 6.8 Hz), 2.49 (t, 2H, J = 6.8 Hz), 2.33-2.15 (m, 2H), 1.26 (t, 3H, J = 7.1 Hz).

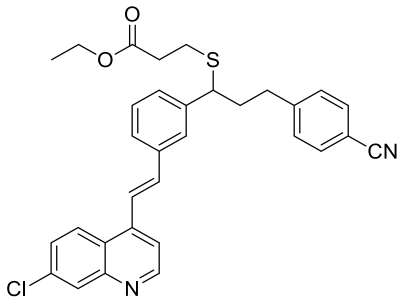

(E)-ethyl-3-((1-(3-(2-(7-chloroquinolin-4-yl)vinyl)phenyl)-3-(4-cyanophenyl)propyl)thio)propanoate (**60**)

Obtained according to general procedure F from **55**. Colorless oil (0.227 g, 46%). MS (ESI+): m/z = 541 $[M+H]^+$; ^1H NMR ($CDCl_3$) δ (ppm): 8.93 (d, 1H, *J* = 4.7 Hz), 8.21 (m, 2H), 7.79 (d, 1H, *J* = 16.1 Hz), 7.64 (d, 1H, *J* = 4.7 Hz), 7.62-54 (m, 5H), 7.44 (t, 1H, *J* = 7.6 Hz), 7.38 (d, 1H, *J* = 16.1 Hz), 7.33 (d, 1H, *J* = 7.7 Hz), 7.29 (m, 2H+$CDCl_3$), 4.15 (q, 2H, *J* = 7.2 Hz), 3.84 (t, 1H, *J* = 7.5 Hz), 2.81-2.74 (m, 2H), 2.65-2.61 (m, 2H), 2.51-2.47 (m, 2H), 2.29 (m, 2H), 1.26 (t, 3H, *J* = 7.2 Hz).

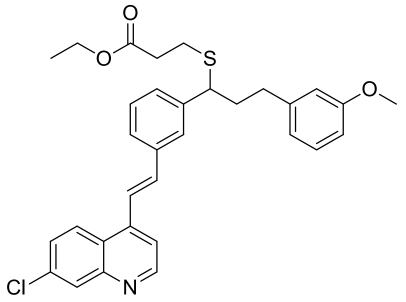

(E)-ethyl 3-((1-(3-(2-(7-chloroquinolin-4-yl)vinyl)phenyl)-3-(3-methoxyphenyl)propyl)thio)propanoate (**61**)

Obtained according to general procedure F from **56**. Colorless oil (0.249 g, 47%). MS (ESI+): m/z = 546 $[M+H]^+$; ^1H NMR ($CDCl_3$) δ (ppm): 8.93 (d, 1H, *J* = 4.6 Hz), 8.28-8.17 (m, 2H), 7.79 (d, 1H, *J* = 16.1 Hz), 7.65 (d, 1H, *J* = 4.6 Hz), 7.62-7.54 (m, 3H), 7.46-7.35 (m, 3H), 7.23 (t, 1H, *J* = 7.9 Hz), 6.80-6.71 (m, 3H), 4.14 (q, 2H, *J* = 7.1 Hz), 3.88-3.84 (m, 1H), 3.81 (s, 3H), 2.69-2.61 (m, 4H), 2.51-2.47 (m, 2H), 2.29-2.22 (m, 2H), 1.26 (t, 3H, *J* = 7.1 Hz).

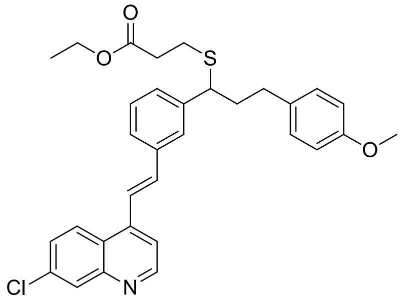

(E)-ethyl 3-((1-(3-(2-(7-chloroquinolin-4-yl)vinyl)phenyl)-3-(4-methoxyphenyl)propyl)thio)propanoate (**62**)

Obtained according to general procedure F from **57**. Colorless oil (0.207 g, 55%). MS (ESI+): m/z = 546 $[M+H]^+$; ^1H NMR ($CDCl_3$) δ (ppm): 8.93 (d, 1H, *J* = 4.6 Hz), 8.22 (d, 1H, *J* = 16.0 Hz), 8.21 (d, 1H, *J* = 4.8 Hz), 7.79 (d, 1H, *J* = 16.0 Hz), 7.64 (d, 1H, *J* = 4.7 Hz), 7.59 (dd, 1H, *J* = 9.0 and 2.2 Hz), 7.57-7.55 (m, 2H), 7.48-7.31 (m, 3H), 7.10 (d, 2H, *J* = 8.6 Hz), 6.86 (d, 2H, *J* = 8.6 Hz), 4.14 (q, 2H, *J* = 7.2 Hz), 3.86-3.79 (m, 4H), 2.66-2.60 (m, 4H), 2.51-2.47 (m, 2H), 2.28-2.13 (m, 2H), 1.26 (t, 3H, *J* = 7.2 Hz).

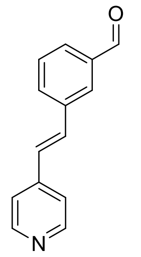

(E)-3-(2-(pyridin-4-yl)vinyl)benzaldehyde (**63**)

Obtained according to general procedure A from 3-vinylbenzaldehyde and 4-bromopyridine. White solid (0.032 g, 10%). MS (ESI+): m/z = 210 $[M+H]^+$; ^1H NMR ($CDCl_3$) δ (ppm): 10.08 (s, 1H), 8.63 (d, 2H, *J* = 6.0 Hz), 8.08 (s, 1H), 7.85 (d, 1H, *J* = 7.5 Hz), 7.80 (d, 1H, *J* = 7.5 Hz), 7.59 (t, 1H, *J* = 7.5 Hz), 7.40 (d, 2H, *J* = 6.0 Hz), 7.35 (d, 1H, *J* = 16.5 Hz), 7.14 (d, 1H, *J* = 16.5 Hz). ^{13}C NMR ($CDCl_3$) δ (ppm): 192.0, 150.3, 144.0, 137.2, 136.9, 132.7, 131.6, 130.0, 129.6, 127.9, 127.5, 121.0.

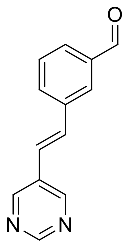

(E)-3-(2-(pyrimidin-5-yl)vinyl)benzaldehyde (**64**)

Obtained according to general procedure A from 3-vinylbenzaldehyde and 5-bromopyrimidine. White solid (0.061 g, 18%). MS (ESI+): m/z = 211 $[M+H]^+$; ^1H NMR ($CDCl_3$) δ (ppm): 10.08 (s, 1H), 9.14 (s, 1H), 8.91 (s, 2H), 8.08 (s, 1H), 7.86 (td, 1H, *J* = 1.2 and 7.6 Hz), 7.80 (d, 1H, *J* = 7.6 Hz), 7.60 (t, 1H, *J* = 7.6 Hz), 7.31 (d, 1H, *J* = 16.8 Hz), 7.13 (d, 1H, *J* = 16.8 Hz). ^{13}C NMR ($CDCl_3$) δ (ppm): 191.9, 157.6, 154.4, 137.0, 136.9, 132.6, 131.2, 130.5, 130.1, 129.6, 127.3, 123.1.

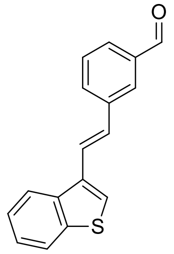

(E)-3-(2-(benzo[b]thiophen-3-yl)vinyl)benzaldehyde (**65**)

Obtained according to general procedure A from 3-vinylbenzaldehyde and 3-bromobenzo[b]thiophene. Colorless oil (0.128 g, 48%). Purity = 81%; tr = 3.40 min; MS (ESI+): m/z = 265 $[M+H]^+$; ^1H NMR ($CDCl_3$) δ (ppm): 10.10 (s, 1H), 8.10 (s, 1H), 8.06 (d, 1H, *J* = 6.4 Hz), 7.93 (d, 1H, *J* = 8.0 Hz), 7.83-7.81 (m, 2H), 7.64 (s, 1H), 7.58 (t, 1H, *J* = 7.6 Hz), 7.53-7.48 (m, 2H), 7.44 (t, 1H, *J* = 7.4 Hz), 7.26 (d, 1H, *J* = 13.2 Hz). ^{13}C NMR ($CDCl_3$) δ (ppm): 192.3, 140.5, 138.4, 137.6, 136.9, 133.6, 132.2, 129.4, 129.0, 128.6, 127.0, 124.7, 124.5, 123.0, 122.8, 122.5, 121.9.

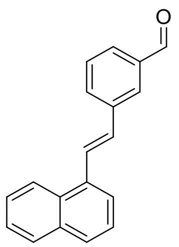

(E)-3-(2-(naphthalen-1-yl)vinyl)benzaldehyde (**66**)

Obtained according to general procedure A from 3-vinylbenzaldehyde and 1-bromonaphtalene. Colorless oil (0.192 g, 74%). MS (ESI+): m/z = 259 $[M+H]^+$; ^1H NMR ($CDCl_3$) δ (ppm): 10.11 (s, 1H), 8.26 (d, 1H, *J* = 8.2 Hz), 8.13 (t, 1H, *J* = 1.7 Hz), 8.01 (d, 1H, *J* = 16.0 Hz), 7.95–7.77 (m, 5H), 7.64–7.50 (m, 4H), 7.21 (d, 1H, *J* = 16.0 Hz). ^{13}C NMR ($CDCl_3$) δ (ppm): 192.3, 138.6, 136.9, 134.4, 133.8, 132.5, 131.4, 130.2, 129.4, 129.0, 128.7, 128.6, 127.6, 127.3, 126.3, 126.0, 125.7, 123.8, 123.7.

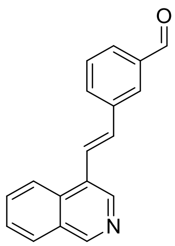

(E)-3-(2-(isoquinolin-4-yl)vinyl)benzaldehyde (**67**)

Obtained according to general procedure A from 3-vinylbenzaldehyde and 4-bromoisoquinoline. Colorless oil (0.139 g, 74%). MS (ESI+): m/z = 260 $[M+H]^+$; ^1H NMR ($CDCl_3$) δ (ppm): 10.11 (s, 1H), 9.23 (s, 1H), 8.80 (s, 1H), 8.20 (d, 1H, *J* = 8.5 Hz), 8.15 (s, 1H), 8.04 (d, 1H, *J* = 7.8 Hz), 7.88–7.77 (m, 4H), 7.68 (t, 1H, *J* = 7.8 Hz), 7.61 (t, 1H, *J* = 7.6 Hz), 7.31–7.24 (m, 1H + $CDCl_3$). ^{13}C NMR ($CDCl_3$) δ (ppm): 192.2, 152.3, 140.5, 138.1, 136.9, 133.8, 132.8, 131.6, 130.7, 129.5, 129.5, 128.3, 128.2, 127.4, 127.3, 124.4, 122.8.

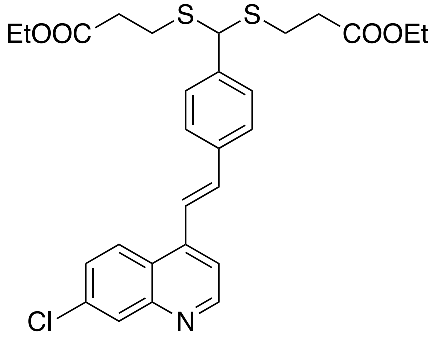

Diethyl 3,3'-(((4-(2-(7-chloroquinolin-4-yl)vinyl)phenyl)methylene)bis(sulfanediyl))(E)-dipropionate (**68**)

Compound **38** (100 mg, 0.34 mmol) and $TsOH{\cdot}H_2O$ (126 mg, 0.68 mmol) were dissolved in toluene (15 mL) and 4Å molecular sieves were added. Allyl thiol (84 µL, 1.02 mmol) and ethyl

3-mercaptopropanoate (129 mg, 1.02 mmol) were added and the reaction mixture was stirred at reflux for 2.5 h under inert conditions. The mixture was diluted with EtOAc and the organic phase was washed with saturated $NaHCO_3$ and brine, dried over Na_2SO_4, filtered and concentrated under reduced pressure. The residue was purified by flash chromatography on a silica gel column (EtOAc:hexanes; 98:2 to 40:60) to afford **68** as yellow solid (17%).

1H NMR (500 MHz, DMSO-d6) δ (ppm): 8.93 (d, 1H, *J* = 4.7 Hz), 8.61 (d, 1H, *J* = 9.1 Hz), 8.10 (d, 1H, *J* = 16.1 Hz), 8.09 (d, 1H, *J* = 2.3 Hz), 7.91 (d, 1H, *J* = 4.7 Hz), 7.85 (d, 2H, *J* = 8.3 Hz), 7.70 (dd, 1H, *J* = 9.1, 2.3 Hz), 7.63 (d, 1H, *J* = 16.1 Hz), 7.51 (d, 2H, *J* = 8.3 Hz), 5.34 (s, 1H), 4.07 (q, 4H, *J* = 7.1 Hz), 2.84 – 2.80 (m, 2H), 2.75 – 2.56 (m, 6H), 1.18 (t, 6H, *J* = 7.1 Hz).

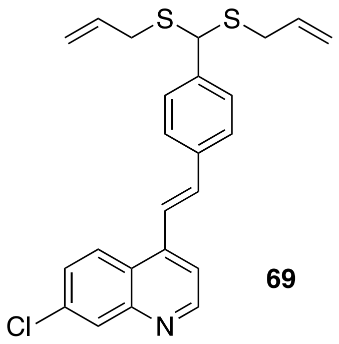

69

*(E)-4-(4-(bis(allylthio)methyl)styryl)-7-chloroquinoline (***69***)*

Further processing of chromatographic fractions from the reaction affording **11** yielded compound **69** (ca 80% pure) as a yellow solid (11%).

1H NMR (500 MHz, DMSO-*d*6) δ 9.17 (d, *J* = 5.6 Hz, 1H), 8.90 (d, *J* = 9.2 Hz, 1H), 8.36 (d, *J* = 2.2 Hz,1H), 8.30 (d, *J* = 5.6 Hz, 1H), 8.25 (d, *J* = 16.0 Hz, 1H), 8.03 – 7.90 (m, 4H), 7.52 (d, *J* = 8.2 Hz, 2H), 5.81 (ddt, *J* = 17.0, 9.9, 7.0 Hz, 2H), 5.19 – 5.10 (m, 4H), 4.96 (s, 1H), 3.31 (dd, *J* = 13.6, 7.1 Hz, 2H), 3.14 (dd, *J* = 13.6, 7.0 Hz, 2H).

SUPPLEMENTAL INFORMATION

REFERENCES:

1. Abagyan RA, Totrov MM, Kuznetsov DA. Icm: A New Method For Protein Modeling and Design: Applications To Docking and Structure Prediction From The Distorted Native Conformation*. J Comp Chem* **15**, 488–506 (1994).
2. An J, Totrov M, Abagyan R. Pocketome via comprehensive identification and classification of ligand binding envelopes*. Mol Cell Proteomics* **4(6),** 752-61 (2005)
3. Totrov M, Abagyan R. Flexible protein-ligand docking by global energy optimization in internal coordinates*. Proteins.* **1997; Suppl 1**, 215-20.
4. Schapira M, Totrov M, Abagyan R. Prediction of the binding energy for small molecules, peptides and proteins. *J Mol Recognit* **12(3),**177-90 (1999).
5. Nguyen, T. G. et al. Development of fluorescent substrates and assays for the key autophagy-related cysteine protease enzyme, ATG4B. *Assay Drug Dev Technol* **12**, 176-189 (2014).
6. Vezenkov, L. et al. Development of fluorescent peptide substrates and assays for the key autophagy-initiating cysteine protease enzyme, ATG4B. *Bioorg Med Chem* **23**, 3237-3247 (2015).
7. Gauthier, Y. G., Henien, T., Lo, L, Thérien, M., & Young, R.N. A Novel and Efficient Method for the Preparation of Asymmetric Dithioacetals, *Tetrahedron Letts*, **29**, 6729-6732 (1988).
8. Zamboni, R. et al. Development of a Novel Series of Styryl Quinoline Compounds as High Affinity Leukotriene D_4 Receptor Antagonists: Synthetic and Structure-Activity Studies Leading to the Discovery of (±)-(3-(3-(2-(7-Chloro-2-quinolinyl)-(E)-ethenyl)phenyl))-3-dimethylamino-3-oxopropylthio)methyl)thio)propionic acid)(MK-571), *J Med Chem*, **35**, 3832-3844 (1992).
